# Modeling Flows and Concentrations of Nine Engineered Nanomaterials in the Danish Environment

**DOI:** 10.3390/ijerph120505581

**Published:** 2015-05-22

**Authors:** Fadri Gottschalk, Carsten Lassen, Jesper Kjoelholt, Frans Christensen, Bernd Nowack

**Affiliations:** 1 Environmental, technical and scientific services-ETSS, CH-7558 Strada, Switzerland; E-Mail: fadri.gottschalk@etss.ch; 2 COWI A/S, Parallelvej 2, Kongens Lyngby, DK 2800, Denmark; E-Mails: CRL@cowi.dk (C.L.); JEK@cowi.dk (J.K.); FMCH@cowi.dk (F.C.); 3 Swiss Federal Laboratories for Materials Science and Technology, EMPA, CH-9014 St. Gallen, Switzerland

**Keywords:** engineered nanomaterials, material flow modeling, environmental concentrations, Denmark

## Abstract

Predictions of environmental concentrations of engineered nanomaterials (ENM) are needed for their environmental risk assessment. Because analytical data on ENM-concentrations in the environment are not yet available, exposure modeling represents the only source of information on ENM exposure in the environment. This work provides material flow data and environmental concentrations of nine ENM in Denmark. It represents the first study that distinguishes between photostable TiO_2_ (as used in sunscreens) and photocatalytic TiO_2_ (as used in self-cleaning surfaces). It also provides first exposure estimates for quantum dots, carbon black and CuCO_3_. Other ENM that are covered are ZnO, Ag, CNT and CeO_2_. The modeling is based for all ENM on probability distributions of production, use, environmental release and transfer between compartments, always considering the complete life-cycle of products containing the ENM. The magnitude of flows and concentrations of the various ENM depends on the one hand on the production volume but also on the type of products they are used in and the life-cycles of these products and their potential for release. The results reveal that in aquatic systems the highest concentrations are expected for carbon black and photostable TiO_2_, followed by CuCO_3_ (under the assumption that the use as wood preservative becomes important). In sludge-treated soil highest concentrations are expected for CeO_2_ and TiO_2_. Transformation during water treatments results in extremely low concentrations of ZnO and Ag in the environment. The results of this study provide valuable environmental exposure information for future risk assessments of these ENM.

## 1. Introduction

Release of engineered nanomaterials (ENM) into the natural environment is expected to happen both unintentionally and intentionally throughout the lifecycle of nanomaterial manufacturing, use and disposal [1,2]. This release can originate from discharges from wastewater treatment plants, landfills and waste incineration plants, all of which are likely to receive ENM from nano-enabled products disposed at the end of their life phase, from accidental spills during production or transport of nanomaterials or from releases during use [3]. Presently, the knowledge on the environmental release and exposure of ENM in the environment is limited. A recent study [4] collected and critically reviewed a dozen studies that modeled environmental concentrations for several ENM (*i.e.*, TiO_2_, Ag, ZnO, CNT, fullerenes and CeO_2_). Significant knowledge gaps are related to difficulties in estimating ENM production and distribution to products, resulting in an uncertain assessment of release of ENM. Mueller and Nowack [5] published the first ENM material flow modeling study, providing scenario-based results for nano-Ag, nano-TiO_2_ and CNT in natural waters, soils and air. The lifecycle-based methodology was later improved by a stochastic and probabilistic methodology [6] that was used to predict probability distributions of mass flows and exposure concentrations for several ENMs (Ag, ZnO, TiO_2_, fullerenes and CNT) in all important environmental compartments such as surface waters, sediments, soils, sewage sludge, sludge-treated soils and air [7]. An updated modeling using newest data on production, use and fate was recently published [8].

Also other models have been applied to model ENM concentrations, some based on simple algorithms [9], on particle flow analysis [10], some just considering a few nano-applications [11,12]. Keller et al. [13] have modeled the flows of ten nanomaterials on a global scale. First attempts have also been made to couple the material flow models with a description of the environmental fate of the particles, obtaining a more accurate estimate of environmental concentrations in surface waters [14,15,16].

The models mentioned above provide average flows and concentrations in standard environmental compartments on a large scale. Some regionalization has been considered, for example by comparing flows in the EU, the US and Switzerland [7], or by single assessments in specific countries, e.g., Ireland [11]. However, due to different model assumptions on production, use and fate, results in different regions using different approaches should not be compared. [17] provide estimates for different regions, based on their global evaluation [13]. The considered regions span the range from very large, e.g., Europe, Asia, to State-wide results in the U.S. to local assessment in one water body. However, the main variable that was used is a *per capita* use and release of ENM. A similar approach has previously been used to provide concentrations at high spatial resolution in a river network [18]. An important aspect in the regionalization of exposure data is that wastewater and solid waste handling is very different in different countries. Whereas some countries landfill all their waste, others use almost exclusively incineration. Also the connection rate to centralized wastewater treatment varies considerably from country to country. Because ENM are known to end up mostly in wastewater and solid waste, a detailed evaluation of these systems in a region is important for an accurate prediction of flows and concentrations.

The current paper provides such a detailed evaluation of ENM flows in Denmark. The modeling considered nine nanomaterials. The model for Switzerland [8] served as main data basis for nano-TiO_2_, Ag, ZnO and CNT by expanding and adjusting input parameter data to the specific situation in Denmark. This involved mainly the handling of wastewater and waste, the per-capita release of ENM but also the geography and volumes of all environmental compartments. The modeling however distinguishes the first time TiO_2_, separately in photostable as well as in photocatalytic form and follows recent nanomaterial fate analysis in waste incineration processes [19]. In addition, this study presents for the first time marine water and sediment exposure results. Additionally, this work provides the first environmental flows and concentrations for quantum dots, carbon black and CuCO_3_ used in wood coatings and provides the first probability distributions for CeO_2_. This paper is based on a detailed report published by the Danish Environmental Protection Agency [20].

## 2. Experimental Section

### 2.1. Environmental Exposure Model

The model used in this work is based on the modeling framework developed to calculate the flows of ENM in different regions [7,8]. In this modeling the release of nanomaterials into the technosphere and different environmental compartments was predicted by considering the complete lifecycle of products containing ENM, including production of ENMs, manufacturing of nano-products, use of nano-products and solid waste handling and wastewater treatment.

For four of the materials covered in this work, a detailed modeling for Switzerland and the EU was published in 2014 [8]. The ENM addressed in this work were nano-TiO_2_, nano-ZnO, nano-Ag and carbon nanotubes (CNT). These data on use (on per capita basis) and the transfer coefficients developed by Sun et al. [8] were applied to Denmark in this study, considering the differences between the regions with respect to waste treatment processes and sizes of environmental compartments. For the other ENM covered in this study (nano-CuCO_3_, nano-CeO_2_, quantum dots (QD) and carbon black (CB)), new input data on production, use and transfer coefficients in technical and environmental compartments were collected.

### 2.2. The Probabilistic Flow Modeling

The probabilistic material flow model used calculates probability distributions of ENM flows and concentrations as done in [7]. The model is based on probabilistic production and use estimates, given in tons or kilograms of use or release of nanomaterial per year, as well as transfer coefficients for release and mass transfer at all stages of the lifecycle of nanoproducts, indicating the fraction of the ENM flowing into another compartment or being eliminated or deposited in final sinks. The lifecycle-based mass transport model tracked the mass of the ENM through all technical compartments, as well as their flows between technical and natural compartments.

The description of the model geometry, the production amounts, product distribution, release quantification as well as all transfer coefficients for the nine ENM are given in the Supporting Information. Sewage treatment plants (STPs) are for most studied ENM the main sources of release to the environment. Transfer coefficients were estimated for the wastewater treatment processes and were obtained for the quantities discharged directly to surface water from rain water overflows and from separate storm water sewer systems. The detailed evaluation of the fate of ENM in STPs and the resulting transfer factors are given in the Supporting Information. The same applies for the complete release spectrum of the investigated materials that considers the complete life cycle of ENM and its products (production of the nanomaterial, manufacturing, nano-product use and consumption as well as landfilling, recycling and waste treatment). The environmental processes sedimentation from water and settling from air were included as follows: sedimentation from water to sediment was modeled by considering the whole probability spectrum from zero to complete sedimentation, settling from atmosphere on soil and water was based on the average residence time of ultrafine particles in air, resulting in almost 100% removal of particles from air over the modeling timescale.

The mathematical modeling is based on matrix algebra, nestled inside iterative computing that connects the ENM flows from production, use, release, transformation and deposition [6,19]. A constant mass transfer for one-year periods is modeled. The lifetime release from the products on the market is allocated to a steady state model for the release period of 2014. The algorithms compute values from the complete model input as probability distributions. These probability distributions are based on measured or experimental data. If these are not available, expert opinions are preferred over assumptions. However, when pure assumptions had to be made to obtain input parameters, we aimed to cover the widest possible parameter space. In cases of total data lack, assumptions were made to derive the maximum and minimum values that form the basis of uniform distributions.

In the computational procedure, the Monte Carlo (MC) algorithms randomly combine all model input values and iteratively solve 100,000 mass balance scenarios for the whole system. This high number of scenarios is indispensable for a robust statistical evaluation of the model outcome. This especially allows a more reliable evaluation for extreme events on both sides of the model output spectrum.

**Figure 1:**
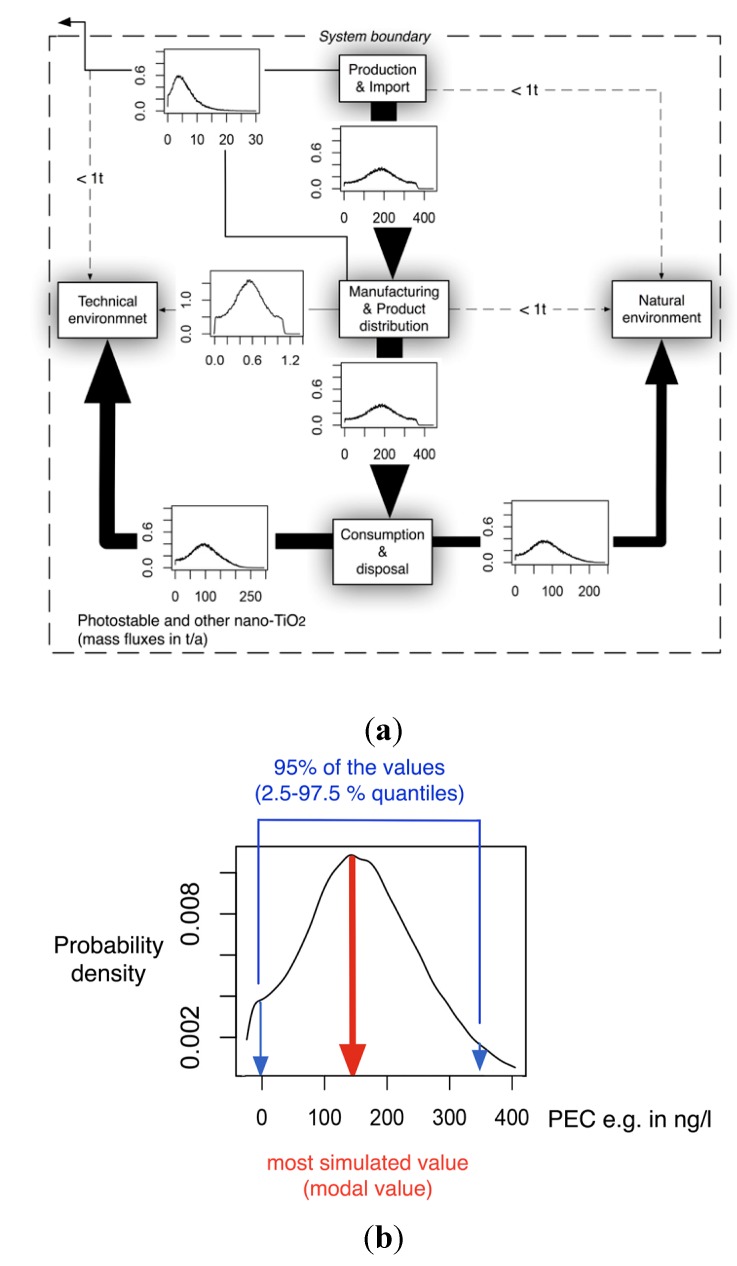
(**a**) Exemplary mass transfer flows in Denmark for nano-TiO_2_, showing the total ENM transport between natural and technical systems. **(b)**: probability distribution with the modal value (red) and the 95% interval (blue) that is given in the results section.

Figure 1 shows an exemplary diagram of the mass transfer flows for nano-TiO_2_ between natural and technical systems. All flows are treated as probability distributions. The right panel shows one exemplary probability distribution of the resulting concentrations in a compartment. The 2.5% and 97.5% quantiles frame the 95% range that provides a 95% probability of the modeled concentrations falling within this range. The modal value, the most frequent value, reflects the most probable exposure concentration. Both the mode value and the 95% range will be given in the Results section.

### 2.3. Nanomaterial Sources

The transfer coefficients for production/manufacturing were modeled by using information of Danish manufacturers as well as sector-specific transfer factors derived from the OECD Emission Scenario Document [21]. According to information obtained in other ongoing surveys [22], sunscreens for the Danish market do in general not contain nano-TiO_2_ as UV-filter, but nano-TiO_2_ may be added to other cosmetic products for sun protection. There is no data available about the use of nano-TiO_2_ in Danish production. Information on potential quantities and the handling of the substances during production could be obtained from the previous survey. Nano-ZnO does presently not belong to the list of approved UV filters for cosmetics in Denmark and the EU, but pigment-grade ZnO may be used.

The production amounts and the distribution to products used in this work are given in detail in the Supporting Information for each ENM. Table 1 gives an overview of main uses of the considered ENM and the amounts used in Denmark. Consumption data for the ENM not covered by [8] were derived from a survey of the use of nanomaterials in the EU [23].

The use of nano-TiO_2_ was divided into two groups of applications in our model: photostable TiO_2_ and photocatalytic TiO_2_. The anatase form of TiO_2_ is a more efficient photocatalyst than the rutile form. As a consequence, most photocatalytic consumer products contain nanosized anatase. The modeling studies published so far did not differentiate between these two groups but just modeled a “generic TiO_2_”. For this study the product groups of [8] were allocated to the two different nano-TiO_2_ forms based on what was considered to be the main application. The most important uses of photostable TiO_2_ are in cosmetics, cleaning agents, plastics and consumer electronics. Cosmetics represent more than half of the total consumption. When taking into account that the major part of this TiO_2_ is released directly to the environment or wastewater, this application is likely the main source of nano-TiO_2_ to the environment. Its application amounts in sunscreens in Denmark may likely be smaller than the EU average, since the Nordic ecolabel (the Swan) forbids the use of nano-TiO_2_ in sunscreens. As quantitative data on the consumption were not available, the data derived from the model of Sun *et al*. [8] were used as a worst-case estimate. The main applications of photocatalytic TiO_2_ considered in [8] were paints, filters for water and air treatment and construction materials (self-cleaning surfaces) as likely smaller application.

**Table 1:** Overview about the considered nanomaterials, their main uses and the amount used per year in Denmark. The curves represent probability distributions in the form of probability density functions.

ENM	Main Uses	Used (t)
Photostable TiO_2_	Plastics, cosmetics	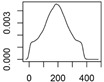
Photocatalytic TiO_2_	Paints, coating, construction materials, filters	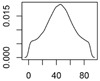
ZnO	Cosmetics, paints	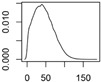
Ag	Textiles, paints, cleaning agents electronics, cosmetics	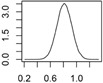
CuCO_3_	Wood preservation	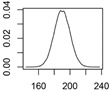
CNT	Polymer composites	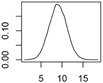
CeO_2_	Catalysts, fuel additive, polishing, paints	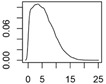
Quantum dots (QD)	LED, imaging	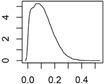
Carbon black (CB)	Tires, rubber, paints	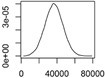

The major application for nano-ZnO considered in [8] accounting for more than half of the consumption was in cosmetics. However, nano-ZnO is not included in the list of UV filters allowed in cosmetic products in the EU and Denmark. Pigment ZnO is allowed, but it is not considered a nanomaterial. For the modeling, the consumption data from [8] were used as worst-case estimate. The only other potential major application from [8] was the use of nano-ZnO in paint. Nano-Ag has various applications, including as antimicrobial compound in textiles, cleaning agents, paints, and cosmetics. Danish use of nanosilver in production processes was currently not identified, and those model parameters were all derived from the previous study for the EU. The main applications of CNTs are as a composite and polymer additive and in electronics. The consumption data were derived from [8].

Nano-CuCO_3_ was not included in the study of [8]. The use of micronized particles of copper carbonate (CuCO_3_) for wood preservatives is considered to potentially account for the major portion of the use of this material [24]. Micronized CuCO_3_ is not intentionally a nanomaterial, but would likely fall within the definition with the current particle size distribution [24]. Micronized copper carbonate is not currently marketed in Denmark, but in the United States the market penetration of micronized copper technology is about 75%–80% [24]. It could be expected that also in the EU micronized CuCO_3_ may substitute for a significant part of the CuCO_3_ currently used for wood treatment. Copper carbonate is applied for the production of pressure-impregnated wood in Denmark, and micronized CuCO_3_ may already be added to imported wood. The use and release of CuCO_3_ was therefore modeled for a scenario of a future situation where micronized CuCO_3_ has substituted the CuCO_3_ used today. For details about the input parameters we refer to the Supporting Information.

The main uses of nano-CeO_2_ in the EU were estimated to be diesel fuel additives, automotive catalyst converters, glass polishing and paint and coatings [23]. For each of these applications, transfer coefficients were estimated (the details are given in the Supporting Information).

For quantum dots (QD), data on production and use were derived from a survey of the use of nanomaterials in the EU [23]. Quantum dots are semiconductor materials of different metals such as Cd and Zn and are considered here as a group and not as an element. Applications of quantum dots in products include medical imaging devices, semiconductors transistors, solar cells, light-emitting devices (e.g., LEDs) and other. Information on the quantum dots manufacturing in Denmark has not been found. On a global scale, 90% of the carbon black (CB) is used in the rubber industry as a filler in a variety of products, with tires as the major application field. In Denmark, the use of carbon black as a black pigment for the manufacturing of inks, paints, cosmetics, plastics and textiles has proved to be relevant. Again, all details about production and use of QD and CB are given in the Supporting Information.

### 2.4. From Mass Flows to Predicted Environmental Concentrations

This work derives the predicted environmental concentrations (PEC) by dividing the flows into or the ENM masses in the compartments by the volumes or masses of the corresponding compartments (given in the Supporting Information). For flow-through compartments such as waste incineration plants (WIP) or freshwater, the ENM inflows into the compartment are transformed into an ENM mass fraction in that compartment. The ENM mass inflows to sinks such as soils or sediments contribute to a continuous, periodic increase of ENM into the compartment. The main model output for these compartments are therefore yearly increases in mass or concentration, respectively.

For the sink compartments, we also modeled concentration scenarios following a nanomaterial deposition period in sediments/soils that began in 2000 and ended at the end of 2014. No use and release as well as environmental accumulation for ENMs were assumed for the time period prior to the year 2000. Starting in 2000, a linear increase in the ENM depositions was simulated by scaling the yearly release calculated for the year 2014. Such an approach has previously been used by [7] to model the increase in ENM concentrations over time.

## 3. Results

### 3.1. Material Flows

**Figure 2:**
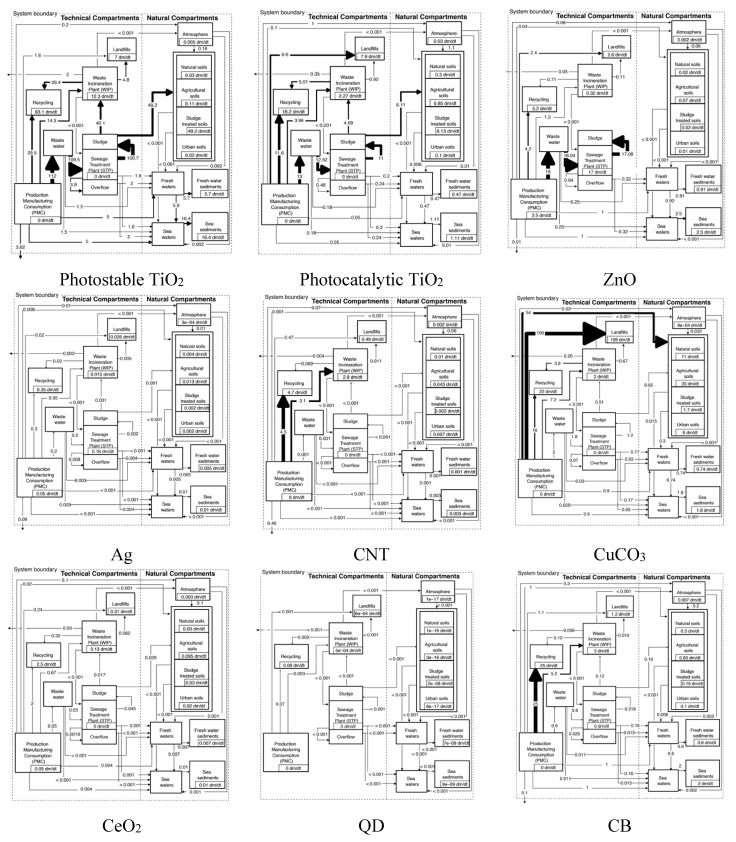
Mass flow diagrams for nine nanomaterials in Denmark. Rounded modal values are shown in tons/year (except CB where the unit is kt/y). Boxes show accumulation or transformation. The modes, combining all the Monte Carlo simulations show what has to be most likely expected at each place of the figure without necessarily reflecting in detail and holistically mass balance of the flow system. CNT: Carbon nanotubes; QD: Quantum dots; CB: Carbon Black.

The material flow diagrams of the nine ENM are shown in Figure 2. In all schemes the flows go from the production/manufacturing/consumption compartment in the bottom left corner to the technical compartments in the middle and then to the environmental compartments on the right. The most important photostable nano-TiO_2_ and nano-ZnO flows go to wastewater due to the prevalent use in cosmetics. Most photocatalytic nano-TiO_2_ finally ends up in recycling and landfills. CNTs show a high mass fraction (up to 90%–95%) ending up in recycling, waste incineration and landfilling processes. Only a marginal environmental release of CNT to both aquatic and terrestrial environments is predicted. Also for nano-CuCO_3_ the main flow is finally to landfills but also direct emission to soils is relevant. The use in wood impregnation strongly leads to a high mass transfer into waste flows with discarded wood (waste incineration, recycling and landfilling). The greatest portion of nano-CuCO_3_ environmental release (approx. 98%) is direct release from the impregnated wood into soils. This discharge occurs via wood-soil contact of those woods under the ground, as well as from leaching processes. The results from the material flow modeling for nano-CeO_2_ gave mass flows into the natural environment which do not exceed half a ton per year. The environmental release mostly occurred via STP sludge application on soils and by limited air emissions. For both nano-Ag and QD only very small flows are predicted, with almost no environmental release for QD and some limited transfer to wastewater for nano-Ag. The carbon black model shows the highest exposure results in this work for all natural compartments. We modeled much higher use volumes (kt per year levels instead of t per year as for the other materials). Because CB is mainly used in rubber, significant release due to material degradation processes (wear and tear) is possible, especially in uses in the environment (tires). However, our model assumed that the whole released fraction of CB would occur as nanoscaled material. These model conditions certainly represent a conservative exposure assessment.

**Figure 3:**
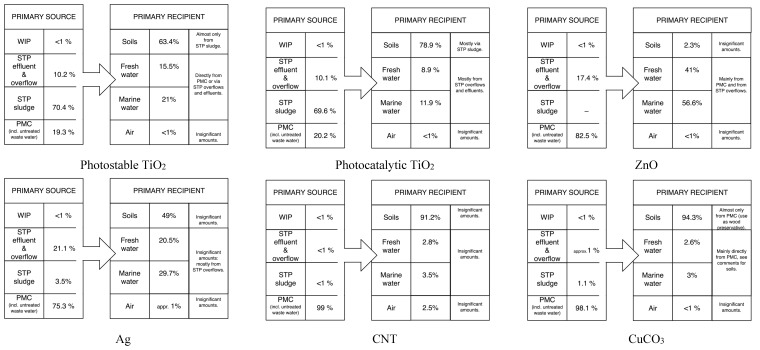

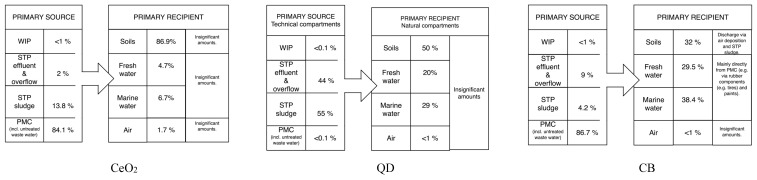
Overview of the most important ENM sources and receivers. The percentages show the most frequently modeled results (modal values).

Figure 3 presents an overview of the most important sources for environmental release and the primary recipients for the nine covered ENMs. For nano-TiO_2_ STP sludge is the main source, with the wastewater effluent as secondary. For other ENM such as ZnO, Ag, CeO_2_ and especially CNT and CuCO_3_, the main source for environmental release is direct release from production/ manufacturing/use. This is because for these materials the major flows after use end up in landfill and WIP where either no release was modeled (landfill) or release is considered to be very low (WIP) [19,25].

The main primary receiving environmental compartment is for most ENM the soil, mainly via application of sludge. Only for ZnO and Ag freshwater is also important because of the almost complete transformation of both compounds during wastewater treatment almost no release occurs with wastewater. Also for CB the aqueous compartments are important due to a completely different release mechanism, the abrasion of tires and direct transfer to the environment.

### 3.2. Concentrations in the Technical System

The concentration values for all technical compartments that were modeled in this work are given in Table 2. These are on the one hand sewage treatment effluent and sewage sludge, on the other hand the solid waste streams solid waste, bottom ash and fly ash. The STP effluents showed the highest concentrations of photostable TiO_2_ of a few to almost 100 µg/L (modal value around 13 µg/L). For photocatalytic nano-TiO_2_ the concentrations are about a factor of 10 smaller. CuCO_3_ is modeled to be present in wastewater at a similar level than photocatalytic TiO_2_ whereas CB has potential concentrations in the mg/L range. Ag, CNT and CeO_2_ have only concentrations in wastewater in the low ng/L range, QD have as maximal value a pg/L. In the solid waste materials concentrations in the mg/kg range can be expected for TiO_2_, ZnO, CuCO_3_ and CB. The other materials are present in the low µg/kg range.

**Table 2:** Concentration of the nine nanomaterials in natural and technical compartments. Mode value and 95% interval are shown, all rounded to two significant numbers. The values for environmental sinks (soils, sediment) represent masses in 2014 accumulated since the year 2000.

Compartment	Unit	Photostable TiO_2_		Photocatalytic TiO_2_		ZnO		CuCO_3_	
		Mode	Range	Mode	Range	Mode	Range	Mode	Range
**Technical compartments**									
Sewage treatment effluent	µg/L	13	3.4–92	1.6	0.4–14	0		1.3	0.3–4.1
Sewage treatment sludge	mg/kg	770	69–1500	85	9.3–230	0		9.1	5.2–17
Waste mass incinerated	mg/kg	15	1.4–32	2.8	0.3–6.8	0.3	0.04–1.5	2	1.3–3
Bottom ash	mg/kg	33	3.4–88	6	0.7–18	0.7	0.1–3.9	4.4	2.7–8.5
Fly ash	mg/kg	170	17–430	30	3.3–90	3.6	0.5–19	22	13–42
**Natural compartments**									
Surface water (fresh water)	ng/L	3	0.6–100	0.27	0.05–7	0.45	0.09–13	2	0.1–6
Sea water	ng/L	0.30	0.04–1	0.02	0.004–0.099	0.04	0.006–0.4	0.04	0.02–0.07
Sediments (fresh water)	µg/kg	1200	200–28,000	92	17–2600	160	30–4800	880	43–2100
Sediments (sea water)	µg/kg	390	49–1300	27	4.3–120	49	6–220	42	25–83
Agricultural soils	µg/kg	0.085	0.01–0.39	0.7	0.1–1.7	0.052	0.008–0.35	28	18–41
Natural soils	µg/kg	0.18	0.024–1.1	1.5	0.2–4.9	0.12	0.018–0.9	60	39–130
Urban soils	µg/kg	0.33	0.039–1.5	2.7	0.3–6.7	0.2	0.03–1.3	110	70–160
Sludge treated soils	µg/kg	1300	130–3100	170	17–480	0	0	48	32–70
Air	ng/m^3^	0.10	0.01–0.5	0.70	0.08–2	0.04	0.005–0.2	0.02	0.005–0.04
	**Unit**	**Ag**		**CNT**		**CeO_2_**		**QD**	
		**Mode**	**Range**	**Mode**	**Range**	**Mode**	**Range**	**Mode**	**Range**
**Technical compartments**									
Sewage treatment effluent	ng/L	0.5	0.012–59	0.3	0.1–3.5	9.3	1.1–60	3.00E−05	5E−6–0.001
Sewage treatment sludge	µg/kg	82	4.2–250	7.6	2.7–62	350	44–2300	2.40E−04	4E−5–0.003
Waste mass incinerated	µg/kg	15	10–23	800	440–1300	180	21–930	0.9	0.1–4.4
Bottom ash	µg/kg	35	21–66	76	27–710	360	50–2500	2.2	0.2–11
Fly ash	µg/kg	170	100–330	330	88–4800	2200	240–12,000	10	1–57
**Natural compartments**									
Surface water (fresh water)	pg/L	15	0–44	1	0.2–15	4	0.6–100	below fg/L	
Sea water	pg/L	0.25	0–0.6	0.05	0.02–0.2	0.3	0.03–2	below fg/L	
Sediments (fresh water)	µg/kg	5.4	0–16	0.5	0.1–5.6	1.6	0.2–45	1.6	0.2–45
Sediments (sea water)	µg/kg	0.3	0–0.7	0.1	0–0.2	0.3	0.04–2	0.3	0.04–2
Agricultural soils	ng/kg	10	6–21	35	18–75	76	10–530	nq	
Natural soils	ng/kg	24	13–61	83	41–220	170	24–1500	nq	
Urban soils	ng/kg	40	23–81	130	71–290	300	39–2100	nq	
Sludge treated soils	ng/kg	170	20–530	60	30–180	1500	94–5100	0.001	1E−4–0.013
Air	ng/m^3^	0.007	0.004–0.011	0.042	0.022–0.091	0.1	0.01–0.6	nq	
**Compartment**	**Unit**	**CB**							
		**Mode**	**Range**						
**Technical compartments**									
Sewage treatment effluent	mg/L	1.2	0.29–3.9						
Sewage treatment sludge	mg/kg	2500	580–7700						
Waste mass incinerated	mg/kg	1400	660–2500						
Bottom ash	mg/kg	140	44–1300						
Fly ash	mg/kg	540	150–8600						
**Natural compartments**									
Surface water (fresh water)	µg/L	0.5	0.1–6						
Sea water	µg/L	0.034	0.015–0.08						
Sediments (fresh water)	mg/kg	730	36–2200						
Sediments (sea water)	mg/kg	41	18–97						
Agricultural soils	mg/kg	0.7	0.3–1.3						
Natural soils	mg/kg	1.5	0.7–3.9						
Urban soils	mg/kg	2.6	1.2–5.2						
Sludge treated soils	mg/kg	5	1.6–17						
Air	µg/m^3^	0.2	0.1–0.3						

### 3.3. Concentrations in Environmental Compartments

All air concentrations were marginal for the studied ENMs, and showed values in the range of pg/m^3^. This mostly reflected very small direct emissions from nanoproduct uses and almost complete removal during waste incineration.

The fresh water photostable nano-TiO_2_ concentrations reached at most 0.1 µg/L while those for seawater were at pg/L concentrations. Soils and sediments were the most significant nano-TiO_2_ sinks, with most likely concentrations for 2014 of 1.3 mg/kg in sludge treated soils and a few tenths of µg/kg in non-sludge-based fertilized soils. In sediments (fresh water and marine water), 1.2 and 0.39 mg/kg were modeled.

The photocatalytic nano-TiO_2_ flow to surface water did only add about one-tenth to the total nano-TiO_2_ flow. The concentrations in surface waters were at most in the ng/L range. Therefore, depending on the various types of ENM applications, the nano-TiO_2_ aquatic relevancy may vary considerably. In the context of risk assessment and toxicology studies, it will be crucial to distinguish these two material categories in the future. Relatively small PECs have been modeled for sediments, and concentrations of approximately 90 µg/kg (freshwater sediment) and 30 µg/kg (seawater sediment) are predicted for 2014. In soils receiving STP sludge, we modeled about 170 µg/kg for 2014.

For nano-ZnO we did not model any significant concentrations, neither in waters nor in soils. The freshwater PECs were mainly in the range of pg/L to a few ng/L, originating from direct release or untreated wastewater; the marine water PECs were by a factor 10 smaller, and all were at pg/L concentrations. Soils and sediments represented the final sinks for nano-ZnO; however, they receive very low quantities. In 2014, around 200 ng/kg are expected in soils, and a few hundred µg/kg in sediments.

The nano-Ag concentrations in freshwater and seawater are at the level of pg/L. Because nano-Ag was almost completely transformed into other chemical forms during wastewater treatment, the soil concentrations were also very low.

CNT concentrations in natural waters only reached pg/L levels; in marine water some fg/L concentrations. We do not expect more than a few µg/kg in freshwater sediments in 2014, also the concentrations in soils is not be above a few dozen ng/kg in that year.

The most likely concentrations of CuCO_3_ are a few ng/L in freshwater and pg/L levels in marine waters. In soils values up to approximately 100 µg/kg of soil could be expected. However, these values reflect our worst-case exposure model, which assumed a significant use of nano-CuCO_3_ in wood impregnation.

The nano-CeO_2_ freshwater and marine water concentrations were at pg/L levels. Concentrations of a few hundred ng/kg are expected in soils, a few µg/kg in STP-sludge-treated soils.

The modeled environmental concentrations of QD in surface waters were so small that a detailed evaluation of their probability distributions was not conducted. The results mostly reflected numbers lower than some fg/L. A few µg/kg are expected in freshwater sediments in 2014 and even lower values for marine sediments.

The CB results in this study are the highest for all natural compartments caused by the much higher use and release amounts (kt per year levels instead of t per year). Since CB is mainly used in rubber, significant release due to wear and tear can be expected. As a conservative approach our model assumed that all the released CB is present as nanosized material. Insofar, the actual nanoparticulate CB exposure concentrations would be expected to be much lower. Less than a µg/L are expected in natural waters, a few mg/kg in soils, mainly due to diffuse input.

## 4. Discussion

Most of the modeled ENM have only a small annual discharge into the natural environment. In most cases (e.g., nano-Ag, CNT, nano-CeO_2_, QD) the flows do not even reach ton per year levels. These results about the total flows to the environment are in line with other European results [7,8]. Notable environmental release and exposure has been found for photostable nano-TiO_2_ and CB in the aquatic and terrestrial environment as well as for nano-CuCO_3_ in soils. This is on the one hand caused by relatively high production amounts for these materials but on the other hand also by their use in products with significant release. In addition there is a lack of transformation reactions for these materials. Transformation reactions are on the other hand very important for ZnO and Ag. For both materials it has been shown that wastewater treatment can efficiently remove them and also transforms them into the respective sulfide forms [26,27,28]. This results in almost zero flows of the parent ENM into the environment through wastewater. Because our modeling is specific for the ENM and does not track the total mass, loss of the nano-form or the parent material constitutes an elimination in our modeling. The flows and concentrations of the ENMs therefore need to be compared to the total flows of the respective metal, e.g., total Zn or Ag [4]. These flows are many orders of magnitude larger and a transformation of a nanoparticle into another form is therefore not significantly increasing the total flow of the respective metal. Also for nano-CuCO_3_ we need to consider transformation reactions because it is relatively soluble depending on the pH and the presence of ions such as carbonate. However, due to the lack of data for this specific material we did not take into account any transformation reactions in our model. The nano-CuCO_3_ concentrations therefore represent a worst-case scenario that is very likely an overestimate. However, the model for nano-CuCO_3_ is also different to the others in that it represents a future scenario under the assumption of wide-spread use of nano-CuCO_3_ in wood protection and does not constitute realistic current flows and concentrations as for all the other materials.

Photocatalytic nano-TiO_2_ has about a factor of 10 smaller concentrations that photostable nano-TiO_2_, a fact that is very important for the environmental risk assessment of nano-TiO_2_. A recent study that quantified the environmental risks of ENM found that nano-TiO_2_ has one of the highest probabilities for environmental risks [29]. This is mainly due to some ecotoxicological studies with photocatalytic TiO_2_ in the presence of UV light that result in very low effect concentrations. Using separate exposure concentrations and different evaluations of the ecotoxicological literature is therefore clearly needed because both forms have very different effects and using a “generic TiO_2_” model is clearly overestimating the possible risks because the more toxic photocatalytic form has lower exposure concentrations than the photostable TiO_2_ used in sunscreens.

In our work we also modeled for the first time QD concentrations. Due to the very low production volume and the use in products with limited release during use, the environmental exposure concentrations are extremely small. Given the fact that many QDs are prone to dissolution under natural conditions [30,31], their actual concentrations will be even lower.

The new data for CeO_2_ and CuCO_3_ can be compared to the results reported by [16] who predicted their concentrations using a simple material flow model. For CeO_2_ concentrations in water between 1 and 10 µg/L were predicted, for Cu between 0.02 and 0.1 µg/L. In sediments the values from that study were around 100 µg/kg for CeO_2_ and 5 µg/kg for Cu. However, it has to be noted that no ranges were given in that study and only single values are reported. The values for CeO_2_ are orders of magnitude larger than what we modeled in our study. Reference [17] provided data for STP effluents for these ENM and reported for San Francisco Bay between 0.02 and 1 µg/L CeO_2_ and 0.001 to 0.01 µg/L for Cu. Our modeled range for CeO_2_ is 1–60 ng/L and 0.3–4.1 µg/L for CuCO_3_, therefore within the range given by [13], for CeO_2_ and above for CuCO_3_. However, also their work does not incorporate any uncertainty with respect to production, use and behavior and the ENM flows that form the basis of the concentration calculation are point values and not ranges. Concentrations for materials with such limited knowledge on production, use and fate as CeO_2_ and CuCO_3_ derived by different models define therefore a range of possible concentrations values.

Knowledge about sedimentation is significant for computing the residence time of the nanomaterials in water, as well as their subsequent transfer into sediments. This affects both the water as well as the sediment concentrations. One limit of our model was that we assumed that the probability of the transfer factor from water to sediment was spread out over the total possible spectrum from complete sedimentation to no sedimentation at all. Depending on the type of ENM, its coating and functionalization and the water chemistry, the stability of ENM in natural waters can vary greatly [32,33]. A coupling of mass flow models with geographically explicit fate modeling of different water bodies is needed to allow an accurate description of aggregation and sedimentation of ENM in natural waters such as presented in [14].

For all ENM we not only provide concentrations in environmental compartments but also in technical compartments such as wastewater, sewage sludge, solid waste, slag and ash from waste incineration. As a large fraction of the total ENM flows end up for many ENM in landfills receiving either directly solid waste or incineration residues [34,35,36], providing data on these materials is important for the current discussion on the nano-relevance of waste. Walser & Gottschalk [19] recently confirmed that for inert metals such as nano-CeO_2_ waste incineration plant processes cannot be seen as a nanomaterial end of life treatment procedures but rather as nanomaterial sources for fly ash and slag that may end up in recycling (and landfills). Depending on the type of ENM, also the flows into recycling are important. These release from recycling operations was not further modeled in this work but recently an evaluation of ENM-flows after recycling has been published [37]. These authors have shown that the main ENM flows after recycling go to landfill and waste incineration. Our model did also not take into account possible release from landfills. For Danish standards of those infrastructures, these seem to be a realistic model assumptions but future research has to investigate the potential for the presence of ENM in landfill leachates and the further dissipation.

Although our model clearly reveals the concentration range that can be expected (as 95% interval), we have to explicitly emphasize the limitations in interpreting the model outcomes. The expected future exposures may be considerably higher if production and use of the investigated ENM increases. The increased electrification of mobility could lead to much higher use increase of ENM, for example in batteries (e.g., QD and CNT). The future expectations for CNTs are large and are anticipated in a broad field of applications (consumer electronics, textiles, polymer composites, *etc*.); however, currently a wide-spread commercially significant use volumes do not seem to be observed. A potential complete market penetration of the use of nano-CuCO_3_ in wood treatment, or nanosized CeO_2_ as a fuel additive, may lead to scenarios with high environmental relevance. More information with respect to volumes of nanomaterial production and use on the part of industry would help considerably in making the exposure modeling more precise. As long as the companies are reluctant to provide the current (and anticipated) quantities of ENM produced and used, the exposure modeling cannot be improved. Because currently good production data are lacking, a forecast of ENM flows and concentrations remains attached with high uncertainties and models needs to incorporate them. Hence, there is a need for a better understanding and more empirical data for the ENM use in different countries and economies, and this by far represents the most important factor necessary for the updating of the environmental exposure assessment of nanomaterials.

Compared to previous published studies with a comprehensive set of ENM that are currently on the market such as published by [13] and [16], our work presents a much more exhaustive list of concentrations in both technical and natural environments and as such the new values are of invaluable help for ecotoxicologists that need knowledge on realistic exposure concentrations for these ENMs. These data are needed both for the design of experiments as well as for critical evaluations of published effect data. Also for analytical chemists these data are very useful because they indicate the range of expected concentrations that new methods need to be able to detect in different media.

Concluding, one must underline that we are still faced with distinct difficulties regarding the secure validation of all kinds of predicted exposure results for ENM. This will not change as long as trace analytical approaches are not applicable for the pollutant quantification and detection at trace levels of nanomaterials. Recent reviews [4,38] evaluated all available modelling and analytical contributions that provide concentrations and found at several occasions accordance between models and measurements. The differences found cover besides the mentioned analytical shortcomings difficulties in the distinction between nanomaterial of anthropogenic and natural origin as well as the limited or very crude consideration in the models of the nanomaterial fate in the environment. However, as far as the whole possible range of events for such nanomaterial fate is considered, the large spectrum models do not miss any significant events. Thus, several recent modelling studies [7,13,16] were seen to correlate well for concentrations ranging from water (surface water and sewage treatment effluents) to solid media (soils, biosolids, sediments) [38]. Furthermore, Sun *et al*. [8] showed in a TiO_2_ case study that the probabilistic/stochastic mass balance approach may be used also for predictions of the bulk material of this compound that may be documented and confirmed by measurements. Finally, these stochastic mass balance studies do not promise any unrealistic precision in the results but allow us to consider the entire currently conceivable model input and output spectrum. Hence, we do not have to validate the well established mass balance principles, but must rather ensure that we have at our disposal more and better parameter data.

## 5. Conclusions

For the first time the flows and concentrations of nano-TiO_2_ were separated into the photocatalytic and the photostable form, allowing now the material-specific environmental risk assessment of the two forms. We also provide first environmental flows and concentrations of quantum dots, carbon black and CuCO_3_ nanoparticles. The highest concentrations of the nine investigated ENM are expected for carbon black and photostable TiO_2_, caused by the high production volume and use in products with significant release during the use phase. ENM that undergo transformation reaction during wastewater treatment such as ZnO and Ag are only expected at very low concentrations in environmental media. For quantum dots we predict the lowest flows and concentrations of all investigated ENM due to a very small production volume and their use in products with almost no release during use. The concentration ranges in various technical and environmental systems that we estimated will provide valuable exposure information for future risk assessment of these ENM.

## Supplementary Files

Supplementary File 1
