# Supplementary material for: Modeling Flows and Concentrations of Nine Engineered Nanomaterials in the Danish Environment
Source: Int J Environ Res Public Health. 2015 May 22;12(5):5581–602. doi: 10.3390/ijerph120505581 (PMC4454986; doi:10.3390/ijerph120505581)
Supplement: Supplementary File 1 [file ijerph-12-05581-s001.pdf]

Supplementary Information

Modeling Flows and Concentrations of Nine Engineered Nano-materials in the Danish Environment

Fadri Gottschalk [1], Carsten Lassen [2], Jesper Kjoelholt [2], Frans Christensen [2] and Bernd Nowack [3,*]

[1] Environmental, technical and scientific services, CH-7558 Strada, Switzerland; E-Mail: fadri.gottschalk@etss.ch

[2] COWI A/S, Parallelvej 2, Kongens Lyngby, DK 2800, Denmark; E-Mails: CRL@cowi.dk (C.L.); JEK@cowi.dk (J.K.); FMCH@cowi.dk (F.C.)

[3] Swiss Federal Laboratories for Materials Science and Technology, EMPA, CH-9014 St. Gallen, Switzerland

* Author to whom correspondence should be addressed; E-Mail: nowack@empa.ch; Tel.: Tel +41-587-657-692; Fax: +41-587-656-998.

Supporting Information

This Supporting Information is based on the "*Annex I: Parameters for estimation of releases of nano-materials to the environment in Denmark*", published by The Danish Environmental Protection Agency, Copenhagen, Denmark, in 2014, within the project "*NanoDEN - Parameters for estimation of releases of nano-materials to the environment in Denmark*".

Contents

1. GENERAL MODEL PARAMETERS 5
1.1.1 Geographical data 5
1.1.2 Aquatic parameters 6
1.1.3 Waste handling 6
1.1.4 Environmental fate.. 8
1.1.5 Volumes of the technical and environmental compartments 9

2. SUBSTANCE-SPECIFIC MODEL PARAMETERS 13

2.1 Photostable nano titanium dioxide (TiO_2).. 13
2.1.1 Manufacturing and import/export of the substance on its own 13
2.1.2 Formulation in Denmark 13
2.1.3 Import/export and end-use in articles and mixtures 17
2.1.4 Waste water treatment... 22
2.1.5 Solid waste treatment (incineration and landfill) 23
2.1.6 Recycling 24

2.2 Photocatalytic titanium dioxide (TiO2) 26
2.2.1 Manufacturing and import/export of the substance on its own 26
2.2.2 Formulation in Denmark 26
2.2.3 Import/export and end-use in articles and mixtures 28
2.2.4 Waste water treatment... 29
2.2.5 Solid waste treatment (incineration and landfill) 29
2.2.6 Recycling 30

2.3 Zinc oxide (ZnO) 31
2.3.1 Manufacturing and import/export of the substance on its own 31
2.3.2 Formulation in Denmark 31
2.3.3 Import/export and end-use in articles and mixtures 31
2.3.4 Waste water treatment... 36
2.3.5 Solid waste treatment (incineration and landfill) 37
2.3.6 Recycling 37

2.4 Silver (AgNP) 38
2.4.1 Manufacturing and import/export of the substance on its own 38
2.4.2 Formulation in Denmark 38
2.4.3 Import/export and end-use in articles and mixtures 38
2.4.4 Waste water treatment... 44
2.4.5 Solid waste treatment (incineration and landfill) 44
2.4.6 Recycling 45

2.5 Carbon nanotubes (CNT) 46
2.5.1 General description 46
2.5.2 Manufacturing and import/export of the substance on its own 46
2.5.3 Down stream use of CNT for production proceses in Denmark 46
2.5.4 Import/export and end-use in articles and mixtures 46

2.5.5 Waste water treatment.....49
2.5.6 Solid waste treatment (incineration and landfill).....49
2.5.7 Recycling.....50

2.6 CuCO3.....51
2.6.1 Manufacturing and import/export of the substance on its own.....51
2.6.2 Formulation and industrial uses in Denmark.....51
2.6.3 Import/export and end-use in articles and mixtures.....52
2.6.4 Waste water treatment.....53
2.6.5 Solid waste treatment (incineration and landfill).....54
2.6.6 Recycling.....54
2.6.7 Further parameters for a soil exposure scenario.....54

2.7 Cerium dioxide (CeO_2).....55
2.7.1 General description.....55
2.7.2 Manufacturing and import/export of the substance on its own.....55
2.7.3 Formulation in Denmark.....55
2.7.4 Import/export and end-use in articles and mixtures.....57
2.7.5 Waste water treatment.....61
2.7.6 Solid waste treatment (incineration and landfill).....61
2.7.7 Recycling.....62

2.8 Quantum dots.....63
2.8.1 General description.....63
2.8.2 Manufacturing and import/export of the substance on its own.....63
2.8.3 Formulation in Denmark.....63
2.8.4 Import/export and end-use in articles and mixtures.....63
2.8.5 Waste water treatment.....65
2.8.6 Solid waste treatment (incineration and landfill).....66
2.8.7 Recycling.....67

2.9 Carbon black.....68
2.9.1 General description.....68
2.9.2 Manufacturing and import/export of the substance on its own.....68
2.9.3 Formulation in Denmark.....69
2.9.4 Import/export and end-use in articles and mixtures.....71
2.9.5 Waste water treatment.....75
2.9.6 Solid waste treatment (incineration and landfill).....76
2.9.7 Recycling.....76

3. REFERENCES.....77

1. General model parameters

Table S1: The model parameters for the geographic description and the volume/mass of compartments

Name of parameter	Unit	Value	Remark, data source
**1.1.1 Geographical data**			
**Population of Denmark**	mill. inhabitants	5.60	Population 2012 (Statistics Denmark, 2013).
**Population of the EU**	mill. inhabitants	504.4	Wikipedia, List of European Union member states by population
**Total area of Denmark**	km^2	43,000	Southern Denmark including islands (not including Faroe Islands or Greenland). (Statistics Denmark, 2013)
**Height of the air compartement**	km	1	Height of air considered affected.<br>ECB, 2003. Technical Guidance Document on Risk Assessment. European Chemicals Bureau. Institute for Health and Consumer Protection, European Commission, Dublin.
**Area of natural soil**	%	21	12% forest + 9% grasslands, heather and moors etc.<br>(Gyldendal, 2013)
**Area of agricultural soil**	%	66	Agriculture and horticulture (Gyldendal, 2013)
**Area of urban soil**	%	11	Urban areas and infrastructure (roads, railways, airports) (Gyldendal, 2013)
**Area of sludge treated soil**	km^2	800	Estimate based on max. 7 tons dw/ha/year according to the Danish regulations on use of sludge on soils (Statutory Order No. 1650/2006). (DEPA, 2009 and DANVA, 2009a)
**Mean depth of natural soil**	m	0.05-0.1	Depth of agricultural soil compartment considered.<br>Low average value: ECB, 2003. Technical Guidance Document on Risk Assessment. European Chemicals Bureau. Institute for Health and Consumer Protection, European Commission, Dublin.<br><br>Hi average value: Vanwalleghem, T., Poesen, J., McBratney, A., Deckers, J., 2010. Spatial variability of soil horizon depth in natural loess-derived soils. Geoderma 157, 37-45.
**Mean depth of agricultural soil**	m	0.2	Depth of agricultural soil compartment considered.<br>ECB, 2003. Technical Guidance Document on Risk Assessment. European Chemicals Bureau. Institute for Health and Consumer Protection, European Commission, Dublin.
**Mean depth of urban soil**	m	0.05	Depth of agricultural soil compartment considered.<br>ECB, 2003. Technical Guidance Document on Risk Assessment. European Chemicals Bureau. Institute for Health and Consumer Protection, European Commission, Dublin.
**Soil density**	kg m-3	1500	The dry soil density 1500 kg m-3 was computed by neglecting the water content from the standardized 1700 kg.<br>ECB, 2003. Technical Guidance Document on Risk Assessment. European Chemicals Bureau. Institute for Health and Consumer Protection, European Commission, Dublin.

Name of parameter	Unit	Value	Remark, data source
**Water-covered surface (fresh water)**	km^2 and %	700 (2 %)	Lakes and rivers (Statistics Denmark, 2013)
**Water-covered surface (sea waters)**	km^2	31,500	Estimate of area within limit of territorial waters (12 nautical miles = 22 km) based on visual assessment of a map of Denmark
**Mean depth of fresh water**	m	3	Depth of water compartment considered. ECB, 2003. Technical Guidance Document on Risk Assessment. European Chemicals Bureau. Institute for Health and Consumer Protection, European Commission, Dublin.
**Mean depth of the sea**	m	10	Rough estimate of mean depth within limit of territorial waters (12 nautical miles = 22 km) based on visual assessment of a map of Denmark showing sea depth isocurves.
**Sediment density**	kg m^{-3}	260	The dry sediemnt density was computed by neglecting the water content from the standardized 1300 kg m^{-3} resulting in 260 kg m^{-3}. ECB, 2003. Technical Guidance Document on Risk Assessment. European Chemicals Bureau. Institute for Health and Consumer Protection, European Commission, Dublin.
**Coast line (sea water)**	km	7,300	Official figure (Gyldendal, 2013). Use of it for calculation of the sea volume will lead to a serious overestimate.
**Coast line (fresh water)**	km	128,000	Official figure (Gyldendal, 2013). The length of the Danish shoreline (7300 km) is the length of the shoreline to the sea. The total length of Danish river and streams (mainly the latter) is 64,000 km of which 48.00 km are streams with a width of <2.5 metres, while 14.500 km are between 2.5-8.0 metres wide and only 1.500 km are >8 metres wide. And this has to be multiplied by 2 as each stream/river has two banks/shores.
**Sea volume relevant (for discharge of ENM: coastal length, distance from the coast and water depth**	km^3	350	Estimate using the width of the Danish territorial waters (12 nautical miles = 22 km) and the estimated mean sea depth within this limit (10 m).
**Locations of the sewage treatment plants (STP), distance from the river source**	km		All the largest STPs in Denmark are located at the coast. A list is provided separately, which gives the names/locations and effluent volumes (2011) of the 12 largest STPs (data from Danish Nature Agency, 2012).
**1.1.2 Aquatic parameters**			
**Daily water consumption per inhabitant**	l/d	130	In 2011 the total effluent volume from Danish STPs was 769 mill. m^3 corresponding to 137 l/d/person. The figure includes the volume of urban runoff in combined sewers, therefore some litres have been subtracted (Danish Nature Agency, 2012)
**Residence time of water in rivers (from the source to the stream mouth into the sea)**	days	2-2.5	The mean velocity is 0.33 m/s, but in the largest rivers the velocity is typically a little higher i.e. 0.4-0.5 m/s. This gives a residence in the longest river of approx. 3.5-4 days. The average is estimated at some 2-2.5 days.
**Fraction of the wastewater treatment plants connected to a) freshwater and b) sea water**	%	50 and 50	Danish Nature Agency (2014). Danish Nature Agency (2012).
**1.1.3 Waste handling**			

Name of parameter	Unit	Value	Remark, data source
**Annual sewage treatment sludge production**	t dw/year	130,000	Dry weight (2005).(DEPA, 2009)
**Sludge disposed of to agricultural soils**	%	55	3 % of the agricultural area in DK receives sludge. Dosage is regulated through criteria for N and P per year and a 5 year period. The figures differ from year to year, and from source to source. Estimated average figures.<br>(DANVA, 2009a; Kirkeby *et al.*, 2005; DEPA, 2009)
**Sludge incinerated**	%	45	
**Connection rate of waste water from households and industry to sewage treatment plants**	%	97	Based on estimate of the number of homes not connected to sewers (2006) divided by the total number of homes (Statistics Denmark). (Organisation of the Municipalities & Ministry of the Environment, 2010: Report from a working group regarding the performance related to sewage treatment as part of the consultations in connection with the planning in the water sector (in Danish)
**Sewage treatment plant overflows (due to heavy rain and flood, overflows escaping STP treatment processes)**	% of total water flow to the treatment plants	4	DANVA (2009b): Water in figures (in Danish)
**Fraction of the industrial and household waste ending up in waste incineration plants (WIP)?**	%	24<br>and<br>54	From Danish waste statistis<br>(DEPA, 2013).
**Fraction of the industrial and household waste ending up in recycling processes**	%	appr.<br>67<br>and<br>38	From Danish waste statistis<br>DEPA, 2013
**Fraction of the industrial and household waste ending up in landfills**	%	appr.<br>4<br>and<br>4	From Danish waste statistis<br>DEPA, 2013
**MSWI: burning, filtration, and acid washing**			Detailed modelling according to (Walser and Gottschalk, 2014). See also the following information.
**Use of bottom ash in construction works**	%	100	Bottom ash is recycled almost 100 % in road construction, soil consolidation and anti-frost layers under buildings. A few percent are landfilled.<br><br>Virksomhedernes Miljøguide (Environmental guide for entreprises), 2013: Slagger fra affaldsforbrændingsanlæg (Bottom ash from waste incinerators) .
**Fly ash ending up in recycling (cement production), export and landfill**	%	39<br><br>22<br><br>39	According to Sun *et al.* (2014) that base their values on (Walser *et al.*, 2012).

Name of parameter	Unit	Value	Remark, data source
**Landfills: leachate escape to soils waters?**	% of total leachate	0	At one Danish landfill, leachate from inert waste and other less contaminated waste (7%) is leached through the soil to the sea after recirculation. (DEPA, 2010; RenoDjurs I/S, 2013)<br>However, due to the uncertainties that are too large when considered the whole area of Denmark and totally missing data for ENM fate analysis the modellimg was stopped at the landfill compartment by considering it as an ENM sink. This occurred in accordance to a zero leaching out of landfills as suggested by others (Sun *et al.*, 2014).
**Recycling processes: escape to soils waters etc.?**		0	See line above second paragraph.
**Annual volume of slag from WIPs**	t/y	Approx. 850.000	Hansen and Olsen, 2004
**Annual volume of fly ash from WIPs**	t/y	Approx. 53.000	Hansen and Olsen, 2004
**Annual volume of munici-pal solid waste**	t/y	3.8	Computed based on the bottom ash volume: After burning, 1 tonne of MSW there will be e.g. a production of 221 kg of bottom ash (slag) (Salzmann, C. Modelling and Quantification of Emissions from Municipal Solid Waste Incin-eration in Europe. Swiss Federal Institute of Technology Zürich Zurich, 2008).

Name of parameter	Unit	Value	Remark, data source
**1.1.4 Environmental fate**			
**Sedimentation from air**	d/year	10 retention time in air	No quantitative values on ENM deposition from the atmosphere are available. Sedimentation factors were derived as suggested earlier (Sun *et al.*, 2014) from information on life-time of ultrafine particles (Anastasio and Martin, 2001)
**Sedimentation from fresh water**	%	0-100	The sedimentation processes in natural waters could not be considered mechanis-tically due to an inconclusive data situation (Praetorius *et al.*, 2012; Praetorius *et al.*, to be submitted). Due to a highly complex Danish river and lake scenery the mass transfer from the fresh water phase into sediments (not reaching sea water) was accounted for by considering all events between and including two extreme scenarios of complete sedimentation and absolutely no sedimentation.
**Sedimentation from sea water**	%	100	Sea water sediments represent the final sink for ENM that ends up in sea water. The modeled ENM sea water concentrations reflect the worst case situation before the ENM sedimentation process started.
**Terrestric compartments**	na	na	Material fate processes were not conisdered in any kind of soils. All solis are therefore modeled as final sinks.
**Soil-water transfer**	%	Approx. 0.6	ENM in soil may be trasnported to surface waters due to ersoion or during storm events etc. According to others (Sun *et al.*, 2014) 0.549% was used as a transfer factor from soils to surface water, a mean value that has been derived from data of diffuse transfer of linear alkylbenzene sulphonate (LAS) (Kannan *et al.*, 2007).

**Name of parameter**	**Unit**	**Value**	**Remark, data source**
**Dissolution upon contact with water**	%	See data below for each specif-ic material.	The elimination of ENM in the product use phase was modeled as dissolution upon contact with water for different products as indicated below in each specifi case. For carbon based ENM or e.g. for nano-TiO2 such dissolution was not considered.
**1.1.5 Volumes of the technical and environmental compartments**			
**Agricultural soils**	kg	8.3e+12	43,000*10^6*(0.2*0.66*0.97)*0.6*2500<br><br>43,000 km^2 total area of Denmark<br>10^6 is the transformation factor from km2 to m2<br>0.2 m is the depth considered for agricultural soil<br>0.66 is the share of agricultural land area<br>0.97 is the proportion of agricultural land not treated with sewage treatment plant (STP) sludge<br>0.6*2500=1500 kg/m^3 used density of dry soil
**Natural soils**	kg	1.1e+12	43,000*10^6*((0.05-0.1)*0.21) *0.6*2500<br><br>43000 km^2 total area of Denmark<br>10,^6 is the transformation factor from km2 to m2<br>0.05 -0.1 m depth used for natural soil (mean value)<br>0.21 is the share of natural land area<br>0.6*2500=1500 kg/m^3 used density of dry soil
**Urban soils**	kg	3.5e+11	43,000*10^6*(0.05*0.11)*0.6*2500<br><br>43,000 km^2 total area of Denmark<br>10^6 is the transformation factor from km2 to m2<br>0.05 m depth used for natural soil<br>0.11 is the share of urban land area<br>0.6*2500=1500 kg/m^3 used density of dry soil
**Sludge (biosolid) treated soils**	kg	2.4e+11	800*10^6*0.2*0.6*2500<br><br>800 km^2 sludge treated area<br>10^6 is the transformation factor from km2 to m2<br>0.2 m is the depth considered for agricultural soil<br>0.6*2500=1500 kg/m^3 used density of dry soil
**Surface water (fresh water)**	l	2.1e+12	700*10^6*3*1000<br><br>700 km^2 water covered surface (fresh water)<br>10^6 is the transformation factor from km2 to m2<br>3 m is the depth of water compartment considered<br>1000 is the transformation factor from m^3 to litre
Surface water (sea water)	l	3.5e+14	350*10^9*1000<br>350*10^9 m^3 relevant water volume<br>1000 is the transformation factor from m^3 to litre
Sewage treatment plant (STP) effluents	l	2.57288135 e+11	130*365*5,590,000*0.97<br>130 l/head is the daily water consumption<br>5590000 Danish population<br>0.97 is the connection rate to central sewage facilities

Name of parameter	Unit	Value	Remark, data source
Sediments (fresh water)	kg	5.46e+09	700*10^6*0.03*0.2*1300<br><br>700 km2 water covered surface (fresh water)<br>10^6 is the transformation factor from km2 to m2<br>0.03 m sediment depth<br>0.2*1300=260 kg/m^3 density of sediments soil
Sediments (sea water)	kg	2.73e+11	3.5*10^10*0.03*0.2*1,300<br><br>3.5*10^10 m^2 relevant surface (sea water sediment)<br>0.03 m sediment depth<br>0.2*1300=260 kg/m^3 density of sediments soil
Atmosphere	m^3	4.3e+13	43,000*1*10^9<br><br>43000 km^2 total area of Denmark<br>1 km assumed depth of air affected by ENM<br>transformation factor from km^3 to m^3
Sewage treatment plant (STP) sludge	kg	1.3e+08	130,000*1,000<br><br>130,000 t annual sewage treatment sludge volume in Denmark<br>1,000 is the transformation factor from t to kg
Municipal waste for incineration	kg	1.86e +9	2,590,000*1000/7997000*5590000<br><br>2.59 million tons Swiss waste volume scaled to Danish conditions based on the population numbers<br>1000 is the transformation factor from t to kg
Waste incineration plant (WIP) bottom ash	kg	8.5e+08	850,000*1000<br><br>Annual volume of slag from Danish waste incineration plants (WIP)<br>1000 is the transformation factor from t to kg
Waste incineration plant (WIP) fly ash	kg	5.3e+07	53,000*1000<br><br>Annual volume of fly ash from Danish waste incineration plants (WIP)<br>1000 is the transformation factor from t to kg

Waste incineration processes were organized as illustrated i Figure 1 and modelled (see Table 1) as suggested by others (Walser and Gottschalk, 2014).

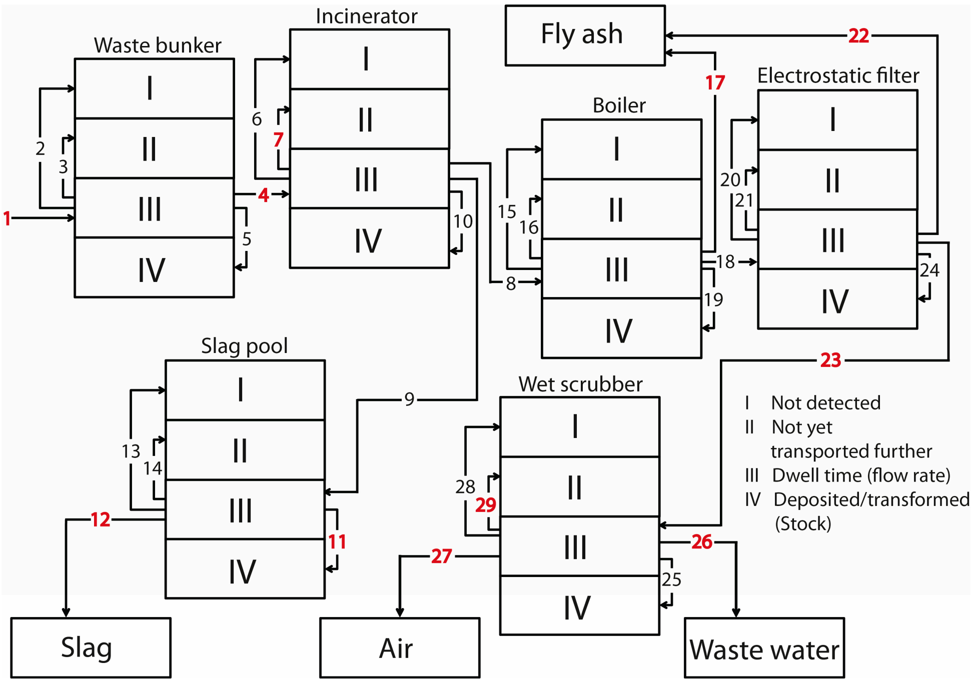

Figure 1. Model structure for the engineered nanomaterial (ENM) transport and fate in waste incineration plants shown in (Walser and Gottschalk, 2014). Red: Measurement points. The ENM transport and transfer were 11odelled based on the measurement data and transfer coefficients between the compartments (and subcompartments) of the prototypical waste incineration plant.

Table S2. Mass transfer and fate fractions for all relevant paths of the metallic ENM studied in a waste incineration plant system derived from (Walser and Gottschalk, 2014).

Transfer Path	Transfer Factors 5% Quantiles	Medians	95 % Quantiles
1.	1.000E+00	1.000E+00	1.000E+00
2.	*Considered either in path 4 or path 5*		
3.	*Considered either in path 4 or path 5*		
4.	9.524E-01	9.762E-01	1.000E+00
5.	1.228E-13	2.379E-02	4.757E-02
6.	*Considered either in path 8, 9 or path 10*		
7.	*Considered either in path 8, 9 or path 10*		
8.	1.179E-01	1.817E-01	2.455E-01
9.	3.625E-01	5.585E-01	7.545E-01
10.	1.232E-13	2.598E-01	5.195E-01
11.	2.679E-28	5.189E-02	1.038E-01
12.	8.962E-01	9.481E-01	1.000E+00
13.	*Considered either in path 11 or path 12*		
14.	*Considered either in path 11 or path 12*		
15.	*Considered either in path 17, 18 or path 19*		
16.	*Considered either in path 17, 18 or path 19*		
17.	3.165E-01	3.328E-01	3.490E-01

Table S2. *Cont.*

Transfer Path	Transfer Factors 5% Quantiles	Medians	95 % Quantiles
18.	6.498E-01	6.504E-01	6.510E-01
19.	8.224E-13	1.683E-02	3.367E-02
20.	*Considered either in path 22, 23 or path 24*		
21.	*Considered either in path 22, 23 or path 24*		
22.	9.039E-01	9.517E-01	9.995E-01
23.	2.023E-04	3.680E-04	5.337E-04
24.	1.265E-12	4.794E-02	9.587E-02
25.	3.352E-09	3.501E-01	7.001E-01
26.	2.999E-01	6.499E-01	1.000E+00
27.	3.352E-09	7.264E-09	1.118E-08
28.	*Considered either in path 25, 26 or path 27*		
29.	*Considered either in path 25, 26 or path 27*		

* The same marked transfer categories do not necessarily exactly add up to one in a particular column of quantiles, for mass balance computations one value has to be derived in dependence of the others.

2. Substance-specific model parameters

2.1 Photostable nano titanium dioxide (TiO_2)

Photostable nano-TiO2 and other applications of nano-TiO2

General applications

A wide range of applications exist for TiO_2 nanomaterials exploiting the various properties of TiO_2 nanomaterials. Pigmentary TiO_2 is widely used as a pigment in paints, whereas nano-scale TiO_2 is widely used in sunscreens and cosmetics due to the UV-absorption of the material. In paints and for water treatment nano-scale TiO_2 is used as a photo-catalyst producing reactive oxygen that may
degrade organic contaminants. Finally, a number of other and very diverse set of applications exists such as ointments, toothpaste, catalysts, catalyst supports, adsorbents, delustrants, semiconductors, etc. In some consumer products, e.g. sunscreens, the percentage of nano-TiO_2 may constitute several percent of the product. TiO_2 rank as one of the most used chemicals world-wide (mainly as a pigment), but the tonnages of nano-TiO_2 used nationally, in the EU or worldwide can at present not be estimated. Given the range of possible applications of nano-TiO_2, the use is anticipated to increase significantly in the near future. (Mikkelsen *et al.*, 2011)

In order to discriminate varying life cycle and release (environmental exposure) pathways the report distribute the use categories of TiO2 nanomaterials reported in Sun *et al.* (2014) into two groups:

- Photostable nano-TiO2 and other nano-TiO2 applications: cleaning agent, spray, cosmetics, paper, plastics, batteries & capacitors, light bulbs, glass & ceramics, consumer electronics, textiles, food, ink, sport goods (covered by the next chapter)
- Photocatalytic nano-TiO2: Paints, metals, cement, filters. (covered by this chapter).

Photostable and other nano-TiO2 applications - It is difficult to define that other applications only use photostable TiO2, it may be a mixture of those properties. Therefore, the second category comprises both application where the photostability of nano TiO2 is applied and other applications. In such applications the chemical stability is crucial, hence, the photocatalytic properties – when exposed to ultraviolet (UV) radiation – have to be avoided/suppressed by coating the TiO2 nanomaterial e.g. with silica and alumina and other (US EPA, 2010a).

Photocatalytic TiO2 - Photocatalytic TiO2 is a material category defined as application where the photolytic effects represent the main target material property. The photocatalytic properties of TiO2 are used in experimental and some commercial fields e.g. for the following purposes: degradation of organic compounds, and destruction of microbiological organisms as well as for transforming e.g. metals to less soluble material forms in waters and air environments (waste and drinking water, indoor air (US EPA, 2010). Photostable nano-TiO2 applications include : e.g. cosmetics, coatings and paints etc.

2.1.1 Manufacturing and import/export of the substance on its own

**Manufacturing processes**	A large number of manufacturing processes exist for ultrafine grade of TiO_2 many of which use either titanium tetrachloride or titanyl sulfate as starting material. These include precipitation, thermal hydrolysis and flame hydrolysis. For the ultrafine grade, the crystal may be further processed involving milling, then coating and milling again. Depending on the medium relevant to the application for marketing, a possible last dispersion step (with water / cosmetic oils) can be applied for example for UV attenuation dispersion grades. If no further dispersion is done, the products obtained are UV attenuation powder grades. Both fine and ultrafine TiO2 may be surface treated to increase their applicability in products, e.g. to ensure a uniform distribution in sunscreens or to optimize UV-absorption properties. (Mikkelsen *et al.*, 2o11)
**Manufacturing in Denmark**	Nanosized TiO_2 is not manufactured in Denmark

**Name of parameter**	**Unit**	**Value**	**Remark, data source**
**Import of the substance on its own or in mixtures to Denmark**	t/year	2.5-30	Estimated as the total of the uses for formulation processes in Denmark mentioned below.
**Re-export**	% of import	no data	No export of the substance on its own has been identified

2.1.2 Formulation in Denmark

**Photostable nano-TiO2 and other applications of nano-TiO2**			
**Identified formulation processes in Denmark**	Titanium dioxide is widely used as a pigment i various mixtures and materials and used for many different formulation processes in Denmark such as manufacture of pigments, paint and varnishes, adhesives, plastics, cosmetics, and food items.<br><br>Intentional use of photostable TiO2 in nanoform for the manufacture of pigments, paint and varnishes, and cosmetics has been confirmed. In all the mixtures the TiO2 is used for UV protection. TiO2 in nanoform may potentially be used to some extent for the manufacture of UV protective plastics and textiles, but an actual use for manufacturing processes in Denmark has not been confirmed.<br><br>A survey of nanomaterials in products on the Danish market indicates the use of titanium dioxide as pigment in ostomy and incontinent devices and plasters (Tønning *et al.*, 2014). According to information obtained from industry, the used titanium dioxide is pigment grade and thus not here considered as nano-material.		
**Name of parameter**	**Unit**	**Value**	**Remark, data source**
**Formulation 1: Production of pigments, paint and lacquers and adhesives**			
**Number of companies**	companies	2-10	The use of TiO2 in nanoform for these formulation processes have been grouped for confidentiality reasons as less than three companies are involved in the production of some of the product types.
**Quantities used**	t/year	2-20	
**Ending up in final products**	%	97%	

**Photostable nano-TiO2 and other applications of nano-TiO2**			
**Release* to municipal waste water system**<br><br>* without any other indications the release values were reduced/ enlarged on each side by 50% for the modeling of symmetrical triangular distributions around the specified quantities. The symmetry may possibly be by the absolute border values (highest or lowest possible release value, 1 and 0). In cases were more values are given, the mean is taken as modeal value for such distributions.	%	<0,25	The emission scenario document (ESD) for the paint industry from the OECD (2009) assume for manufacture of aqueous dispersion coatings that the total fraction of raw materials lost to waste from the manufacturing process is 1.5%. This includes 1% lost due to residues in the mixing vessels and 0.5% due to residues in bags, spills and product returns. It is in the ESD assumed that half of the residue material in the mixing vessels will be re-used in the manufacturing process (recycling. For aqueous dispersion coatings the remaining equipment residue is assumed to be removed in water washings and hence to waste water.<br><br>According to information from Danish manufactures waste water originates from cleaning of tanks and other production equipment. Approximately 1-2% of the total used may be released to the waste water for pre-treatment/treatment at the manufacturing sites.<br><br>The first step at all sites is a precipitation/flocculation where the majority of the TiO2 is precipitated and ends up in a sludge/filter cake which is disposed of for external incineration or gasification.<br><br>The pre-treated waste water is either directed to municipal waste water plants or further treated at the manufacturing sites.<br><br>In the latter case, the waste water is further treated. The waste water is first treated by pre-precipitation tank, then by biological treatment (do not remove TiO2) and ultimately by a final polishing.<br><br>As a worst case estimate, the releases to municipal waste water treatment plants or surface water is estimated to be <0,5%. The actual release is probably significantly below this value. As the pre-treated waste water is either directed to municipal waste water treatment plants or to surface water, the emission to each of the pathways is estimated to be <0.25 %
**Direct release to surface water (after internal WW treatment)**	%	<0,25	Worst case estimate – the total release is probably significantly below the <0.25%.
**Direct release to air**	%		The TiO2 is imported as pastes in which the TiO2 is dispersed in water. The generation of dust by handling of the pastes is considered insignificant.
**Disposed of as solid waste for incineration**	%	2	Filtercake/sludge and TiO2 remaining in packaging are disposed of for incineration or gasification.
**Transformation during use into other forms**	%		Considered insignificant
**Percentage of produced products exported**	% of quantity in final product	Majority	
**Formulation 2: Production of cosmetics**			
**Number of companies**	companies	<4	

**Photostable nano-TiO2 and other applications of nano-TiO2**			
**Quantities used**	t/year	0,5-10	According to a new survey, sunscreens manufactured in Denmark for the Danish market in general do not contain TiO2 in nano-form (Tønning *et al.*, 2014). Most Danish cosmetic producers offer a range of products in compliance with the ecolabel 'Svanemærket' which does not allow the use of nanomaterials with a few exceptions. This has led to a substitution of the former widespread use of titanium dioxide for the benefit of chemical UV filters in sunscreen (Tønning *et al.*, 2014).<br>According to information from Danish manufacturers of cosmetics, nano-TiO2 is still used for UV protection in mascara, eyeliner, face powder and foundation. The use of TiO2 for these applications has been introduced within the last year.<br>A few years ago one of the major manufacturers was reported to use 1-10 tonnes nano TiO2 for sunscreens (Tønning and Poulsen, 2007). It has not been possible to obtain updated information on the use of TiO2 in the Danish cosmetics industry.
**Ending up in final products**	%	95%	
**Release to municipal waste water system**	%	2	Water used to wash containers is handled as common waste water (Tønning and Poulsen, 2007). The percentage is roughly estimated from the experience from other formulation processes.
**Direct release to surface water (after internal WW treatment)**	%		No direct discharges to surface water
**Direct release to soil**	%		No direct releases to soil
**Direct release to air**	%	<0.01	According to Tønning and Poulsen (2007), TiO2 nanoparticles are purchased as a powder. The powders are handled in 25 kg bags which are cut open, hereafter the content is dispersed in the mixture that constitutes the base of the products.<br><br>The OECD has not developed an Emission Scenario Document (ESD) for the cosmetics industry but some of the mixing processes may be comparable with the paint industry. According to the ESD (OECD, 2009) for the paint industry when using pigments and fillers in powder form the emissions are estimated at 0.5-1.0% by weight of the raw materials in powder form. It is supposed, that 95% of this will be removed by the ventilation system. The remaining is assumed to settle on the surfaces inside the workshop. The percentage captured by air extraction systems is assumed to be 95% - and the percentage of solid raw materials released to the atmosphere is estimated at 0.0095% of the raw materials used. The remaining part of the generated dust is assumed to be disposed of as solid waste.
**Disposed of as solid waste for incineration**	%	1	Empty bags containing small amounts of material are disposed as regular waste. (Tønning and Poulsen, 2007). 0.5-1% in the form of generated dust is estimated to be disposed of as regular waste.<br>The percentage is roughly estimated from the experience from other formulation processes.
**Disposed of for other waste management**	%	2	Whole bags and miss productions are disposed as hazardous waste (Tønning and Poulsen, 2007)<br>The percentage is roughly estimated from the experience from other formulation processes.

**Photostable nano-TiO2 and other applications of nano-TiO2**			
**Transformation during use into other forms**	%		Considered insignificant
**Percentage of produced products exported**	% of quantity in final product	no data	
**2.1.3 Import/export and end-use in articles and mixtures**			

	End use	Percentage of total *1 Lower, mean, higher value (l,m,h)
1	Plastics	0, 3.6, 12
2	Cosmetics	0.33, 59.4, 94
3	Cleaning agents	0, 6.2, 16
4	Consumer electronics	0,6.9, 18
5	Battery	0, 0.4, 2
6	Light bulb	0, 0.2, 1
7	Glass & ceramics	0, 1.7, 10
8	Textiles	0,0.3, 1
9	Food	0, 0.4, 2
10	Paper	0, 0.003, 0.02
11	Ink	<0.0003
12	Sporting goods	0, 1.5 , 6
13	Spray	0, 0.2, 1
14	Metals	0, 0.1, 1

1 Note that these values due not sum up to 100% since they refer to the total of both applications (photostable and photocatalytic) nano-TiO2

**Photostable nano-TiO2 and other applications of nano-TiO2**	
**Identified uses in articles and mixtures**	In the model, the total use in Denmark will be computed by scaling raw data from other regions into Danish dimensions based on the comparison of the population figures. Sun *et al.* (2014) summarize current available quantitative estimations: The US EPA (EPA, 2010) reports for the global use/production of nano-TiO2 12’500 t/y; while a study by Nightingale *et al.* (2008) suggests such amounts being around 5’000 t/y. A market analysis from 2011 (FutureMarkets, 2011a) indicates that the global production of nanoparticle TiO2 in 201o was 50,400 t/y and project the production to reach 201,500 tons by 2013. Dupont (2010) indicates the global market to be less than 26,000 t/y in 2010. Considering the different estimates, most likely the total global consumption of nano TiO2 will be in the range of 10,000-50,000 t/y.<br><br>Piccinno *et al.* (2012) provided an industry survey indicating European use ranging from 55 to 3’000 tons. The figures are reached by adding up the estimates for each application area. Others (Hendren *et al.*, 2011) reported for US production volumes a range between 7’800 and 44’400 tons. Schmid *et al.* (2008) came up with survey based production/use volumes per year of nano-TiO2 for Switzerland of 436 tons.<br><br>On the basis of the survey of use of nano-TiO2 in Europe (Piccinno *et al.*, (2012)), the total consumption of nano-TiO2 (both photostable and photocatalytic) in Denmark with finished products (import and domestic production) is roughly estimated at 0.6-465 t/y. This range is used to complement the annual use volume modelled in a reference study by others (Sun *et al.*, 2014) and scaled down for Danish conditions. The latter work uses raw values (for Switzerland and appr. by a factor 1.43 higher than equivalent Danish figures) that range from 1.8t/a (Piccinno *et al.*, 2012) to 1’606 t/a (Schmid and Riediker, 2008). This approach seems to be rather conservative and result in a very high uncertainty, but is used in order to the approach is used in order to ensure that the model estimates span the full range of possible outcomes. Based on the ENM use categorization in Sun *et al.* (2014) we could attribute in average 19.2 % of TiO_2 nanomaterial to the photocatalytic titanium and the rest to the material categorization photostable and other nano-TiO_2.<br><br>Lower, upper boundary and mean mass fraction of nano-TiO2 modelled for the allocation to different product applications represent the basis for computing nano-TiO2 release from those applications.<br><br>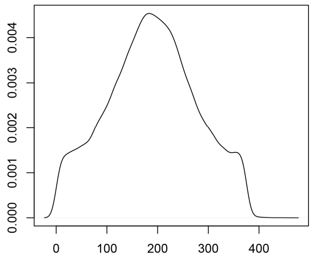<br><br>Figure 2 Annual and total nano-TiO2 use in Denmark (t/a).

Photostable nano-TiO2 and other applications of nano-TiO2	
	Such a release model tracks nano-TiO2 emissions throughout the complete life cycle of these categories.<br><br>The distribution on end uses and parameter values are taken from the 2014 study (Sun *et al.*, 2014) and reflect the fraction of the total nano-TiO production that is used in a specific product category. The mean, the lower and upper limit values describe the average quantities of triangular shaped probability distributions produced via the used MC model procedure (Gottschalk *et al.*, 2010).<br><br>In order to count the relevant (only commercially available) nanoproducts and allocate them to these product categories, data from several sources was used for this allocation (Sun *et al.*, 2014):<br>• Company survey with direct distributional data (Piccinno *et al.*, 2012)<br>• Inventories of nano-products: Woodrow Wilson Centre for Scholars' Project on Emerging Nanotechnologies (Woodrow Wilson Institute, 2012) ANEC/BEUC Inventory (ANEC/BEUC, 2010); the BUND inventory (BUND, 2011); nanotechnology patents (Lem *et al.*, 2012)<br>• A market report (Future Markets, 2011).<br>• Internet search on Google, Yahoo and EC21 in order to count the numbers of products on the market.<br><br>The knowledge available from these data were either the fraction of ENM in a particular application category or the product numbers containing such ENM.<br>Lower, upper boundary and mean mass fraction of ENM modelled for the allocation to different product applications. Details on such computation from different sources are explained in detail below and exemplary for the nano-TiO2 and based on a recent study (Sun *et al.*, 2014).<br>The distribution on end-uses of nano-TiO2 presented by Sun *et al.*, 2014 is here divided into two groups: photostable and other applications of TiO2 (this section) and photoactive TiO2 (next section).

Photostable nano-TiO2 and other applications of nano-TiO2			
**Name of parameter**	**Unit**	**Value**	**Remark, data source**
**End-use 1: Plastics**			
**Total consumption**	% of total consump-tion of TiO_2	3.6	Percentage (mean value) of the total nano-TiO2 use (Sun *et al.*, 2014).
**Release to municipal waste water system**	%		
**Disposed of to MSWI**	%	100	
**End-use 2: Cosmetics**			
**Total consumption**	% of total consump-tion of TiO_2	59.4	Percentage (mean value) of the total nano-TiO2 use (Sun *et al.*, 2014).
**Release to municipal waste water system**	%	85	
**Direct release to surface water**	%	10	
**Disposed of to MSWI**	%	5	
**End-use 3: Cleaning agent**			
**Total consumption**	% of total consump-tion of TiO_2	6.2	Percentage (mean value) of the total nano-TiO2 use (Sun *et al.*, 2014).
**Release to municipal waste water system**	%	95	
**Disposed of to MSWI**	%	5	
**End-use 4: Consumer electronics**			
**Total consumption**	% of total consump-tion of TiO_2	6.9	Percentage (mean value) of the total nano-TiO2 use (Sun *et al.*, 2014).
**Disposed of to MSWI**	%	5	
**Disposed of for recycling (excl. energy recovery)**	%	75	
**Export**	%	20	
**End-use 5: Batteries and Capacitors**			
**Total consumption**	% of total consump-tion of TiO_2	0.4	Percentage (mean value) of the total nano-TiO2 use (Sun *et al.*, 2014).
**Disposed of to MSWI**	%	13	
**Disposed of for recycling (excl. energy recovery)**	%	33	
**Export**		54	
**End-use 6: Light bulbs**			

**Photostable nano-TiO2 and other applications of nano-TiO2**			
**Total consumption**	% of total consump-tion of TiO_2	0.2	Percentage (mean value) of the total nano-TiO2 use (Sun *et al.*, 2014).
**Disposed of to MSWI**	%	80	
**Disposed of for recycling (excl. energy recovery)**	%	20	
**End-use 7: Glass and ceramics**			
**Total consumption**	% of total consump-tion of TiO_2	1.7	Percentage (mean value) of the total nano-TiO2 use (Sun *et al.*, 2014).
**Release to municipal waste water system**	%	1	
**Disposed of to MSWI**	%	20	
**Disposed of for recycling (excl. energy recovery)**	%	79	
**End-use 8: Textiles**			
**Total consumption**	% of total consump-tion of TiO_2	0.3	Percentage (mean value) of the total nano-TiO2 use (Sun *et al.*, 2014).
**Release to municipal waste water system**	%	1	
**Direct release to air**	%	1	
**Disposed of to MSWI**	%	50	
**Export**	%	48	
**End-use 9: Food**			
**Total consumption**	% of total consump-tion of TiO_2	0.4	Percentage (mean value) of the total nano-TiO2 use (Sun *et al.*, 2014).
**Release to municipal waste water system**	%	90	
**Disposed of to MSWI**	%	10	
**End-use 10: Ink**			
**Total consumption**	% of total consump-tion of TiO_2	<0.003	Not quantified in the model due to extremely low assumed amounts.
**Release to municipal waste water system**	%	80	Percentage (mean value) of the total nano-TiO2 use (Sun *et al.*, 2014).
**Disposed of to MSWI**	%	20	Percentage (mean value) of the total nano-TiO2 use (Sun *et al.*, 2014).
**End-use 11: Paper**			

**Photostable nano-TiO2 and other applications of nano-TiO2**			
**Total consumption**	% of total consump-tion of TiO_2	0.003	Percentage (mean value) of the total nano-TiO2 use (Sun *et al.*, 2014).
**Release to municipal waste water system**	%	10	
**Disposed of for recycling (excl. energy recovery)**	%	80	
**Export**	%	10	
**End-use 12: Sporting goods**			
**Total consumption**	% of total consump-tion of TiO_2	1.5	Percentage (mean value) of the total nano-TiO2 use (Sun *et al.*, 2014).
**Release to municipal waste water system**	%	2	
**Direct release to air**	%	2	
**Disposed of to MSWI**	%	96	
**End-use 13: Spray**			
**Total consumption**	% of total consump-tion of TiO_2	0.2	Percentage (mean value) of the total nano-TiO2 use (Sun *et al.*, 2014).
**Release to municipal waste water system**	%	85	
**Direct release to air**	%	10	
**Disposed of to MSWI**	%	5	
**End-use 14: Metals**			
**Total consumption**	% of total consump-tion of TiO_2	0.1	Percentage (mean value) of the total nano-TiO2 use (Sun *et al.*, 2014).
**Release to municipal waste water system**	%	5	Release specific data for all end uses were used as presented in the newest study for Swiss conditions (Sun *et al.*, 2014). This seems reasonable since the crucial release parameters e.g. in landfilling, waste and wastewater treatment are e.g. very similar in these coun-tries. Single data points are given. However, according to Sun *et al.* (2014) these single values are deviated on each side by 50% and a triangular distribution computed.
**Disposed of to MSWI**	%	5	
**Disposed of for recycling (excl. energy recovery)**	%	90	

2.1.4 Waste water treatment

**Name of parameter**	**Unit**	**Value**	**Remark, data source**
**Transformation during STP treatment into other forms**	%	0	See next line.

**Photostable nano-TiO_2 and other applications of nano-TiO_2**			
**Percentage ending up in sludge**	% (l, m, u)	20, 60, 98	We modelled empirical distributions with mean, an the lower and upper limit values as indicated in the column adjacent that follow the newest evidence that in turn is based on the following sources: real plant experiments conducted by others (Westerhoff *et al.*, 2011) showed removal efficiency for Ti between 96.1% to 99.4% with a mean value of 98.3%. Gomez-Rivera *et al.* (Gomez-Rivera *et al.*, 2012) (2012) showed in a laboratory-scale activated sludge system the removal of CeO_2 that probably can also be used for a TiO_2 case. These authors came up with a total CeO_2 removal of 96.6%, for the CeO_2 smaller than 200 nm removal efficiency of 98.5% was seen. Other CeO_2 removal experiments in a model wastewater treatment plant and showed an efficiency between 95% and 98% (Limbach *et al.*, 2008). Our values reflect also other sources that cover and confirm the spectrum as describedabove .(Zhang *et al.*, 2008; Hwang *et al.*, 2011; Johnson *et al.*, 2011; Wang *et al.*, 2012)<br>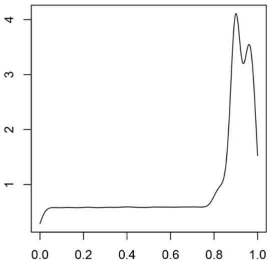<br><br>Figure 3. Modelled probability distribution of STP removal efficiency for generic nano-TiO_2.<br><br>In the model procedure the data from these sources were computed by using weighting factors: the highest factors were given to data taken from full scale STP experiments/measurements, lower ones to evidence taken from pilot STP experiments, and the lowest weighting factor for quantitative values from batch laboratory experiments.
**Percentage discharges**	% (l, u)	2-80	See line above.
**2.1.5 Solid waste treatment (incineration and landfill)**			
**Name of parameter**	**Unit**	**Value**	**Remark, data source**

**Photostable nano-TiO2 and other applications of nano-TiO2**			
**Transformation or deposition during incineration into other forms (average Danish MSWIs)**	%	approx. 0.1-52 (deposition, transformation)	According to Mueller *et al.* (2013) three main processes of waste incineration processes are relevant: i) burning, ii) filtration, and iii) acid washing (wet-flue gas cleaning). We considered the nanoparticle fate during such processes based on measurement evidence for CeO2 (Walser *et al.*, 2012). In line with the above mentioned CeO2 study the partition of all target metallic nanomaterials between waste bunker, incinerator, boiler, electrostatic filter, wet scrubber, slag and fly ash was modelled as done in Walser and Gottschalk (2014).<br>Mass transfer and fate parameters (see please Figure 1) are modelled as shown below in Table 1 that reflects the values derived from computer based simulations combined with real analytic/experimental results. These results show the steady state mass transport/transformation for all relevant WIP paths reached after steady state mode of such plants (infinite time scale). This means that analytically not detected and not further transported material mass has been assigned to the subsequent further transport and/or to the subsequent deposition/transformation by covering at each stage in the WIP process the entire range of transport and fate possibilities. A distinction between material deposition and transformation was not possible due to analytical limitations. However, in contrast to others we quantified also such not further studied mass volumes that were ignored by others (Gottschalk *et al.*, 2009; Sun *et al.*, 2014) assuming zero deposition and transformation for nano-TiO2.
**Percentage emitted to the air (average Danish MSWIs)**	%	~0	See line above.<br><br>The efficiency of filter processes depends on the particle-size rather than on material composition (Sun *et al.*, 2014). Others (Walser *et al.*, 2012) report removal efficiency of filter up to to 99.9%. This is totally in line with earlier evidence (Burtscher *et al.*, 2001) (> 99.5% efficiency) used in other occasions (Gottschalk *et al.*, 2009; Sun *et al.*, 2014). Regarding the removal of acid washing processes the mentioned studies agree on values higher than 99.9%.
**Percentage ending up in residues (average Danish MSWIs)**	%	approx. 36-75 (slag)<br>approx. 3-9 (fly ash)	See line above on nanoparticle deposition transformation for sources and data.<br><br>Reijnders (2005) reports for Denmark over 72% of the ash of waste incineration processes being reused for the construction of cycling tracks, parking lots, roads etc.
**Release from landfills to municipal waste water treatment**	kg/year	0	For landfill, no leachate out is assumed (Sun *et al.*, 2014).
**Direct release from landfills to surface water**	kg/year	0	See line above.
**Transformation during landfilling into other forms**	%	No data	At this point we stopped our modelling. Nanomaterial fate and behaviour during landfilling was not considered. See also general comments on landfilling.
**2.1.6 Recycling**			
**Type of recycling activities**	Recycling of the bottom ash of waste incineration processes		
**Name of parameter**	**Unit**	**Value**	**Remark, data source**

**Photostable nano-TiO2 and other applications of nano-TiO2**			
**Transformation during recycling into other forms**	**%**	No data	Currently quantitative information that could be used to model fate and behaviour of ENM during and after recycling is not available. Hence according to others (Sun *et al.*, 2014), we did not track the material fate and mass flows of the studied nanoparticles during and after the recycling process.
**Ending up in recycled products**	**%**	72 (of the bottom ash of waste incineration processes)	See comments above for percentages ending up in residues (average Danish MSWIs).
**Release from recycling process**	**% of recycled**	0	See first line on transformation during recycling into other forms.

2.2 Photocatalytic titanium dioxide (TiO2)

Photocatalytic titanium dioxide (TiO_2)

General applications

Paints – One use of nano-TiO2 in paint in the intentional use of nano-sized anatase titanium dioxide in paint, which has been developed to exploit the photo-reactive properties of nano-sized anatase titanium dioxide for decomposing organic substances and bacteria on the surface to achieve self-cleaning/antibacterial effects. In the preliminary product screening, a total of 16 paint undertaken by Sørensen *et al.* (2014) products that apply photo-reactive TiO2 particles were identified. Among the paints applied in Denmark is roof paint with photo-reactive TiO2.

Coatings - The use of coatings for surfaces has the explicit purpose of protecting the surfaces from bacteria and other pollutants; the so-called Clean Catalytic Surfaces. Herein, the specific use of nano-sized anatase titanium dioxide is very much desired due to its photocatalytic properties. Many of the commercially available coatings are aimed at the DIY market, where surfaces on buildings and metal, stone and glass can be coated to gain the self-cleaning property. The application techniques of the coatings include spray coating at room temperature or elevated temperatures (400-600 ºC) and application by brush. In the preliminary product screening by Sørensen *et al.* (2014), a total of 43 coating products that contain photocatalytic titanium dioxide particles were identified.

Construction materials - The major applications of titanium dioxide-based photocatalytic construction and building materials are air pollution remediation, self-cleaning and self-disinfection. For all products the driving force is solar light (and the presence of water). Construction and building materials are optimal media for applying the photocatalytic nano materials because large areas are exposed to light. Several pilot projects have been carried out to verify the effectiveness of photocatalytic construction and building materials. Successful commercialization of self-cleaning surfaces includes concrete, mortar, glass, titles and ceramic products. These products enable buildings to maintain their aesthetic appearance over time. Similar, a number of self-disinfecting building materials have been commercialized to achieve a microorganism free environment. Lastly, a number of building materials enable of decomposing air pollutant (including volatile organic compounds and oxides such as NO, NO2 and SO2) have been commercialized; including roofing felt and pavement blocks.

Water treatment systems - Photocatalytic water treatment systems are applied for the removal of trace contaminants and pathogens (Savage and Diallo 2005; Qu, Alvarez *et al.* 2013 as cited by Tønning *et al.* (2014)). Two configurations are commonly used: slurry reactors and immobilized. Photocatalytic water-treatment applications have almost become a mature market as systems based on artificial UV light have been on the market for several years and systems for treating municipal, industrial, swimming facility, drinking and ballast water are also available (Saari, Iler *et al.* 2010 as cited by Sørensen *et al.* (2014)).

Photocatalytic UV irradiation using titanium dioxide nanoparticles as a catalyst is applied for removal of bacteria and other pollutants in water treatment systems, air cleaners and construction materials. Contrary to the product groups mentioned above, the nano-sized titanium dioxide has been deposited as a thin film to the surface of most of these product groups at the time of purchase. Most thin films are synthesized using a gas phase method, i.e. chemical/chemical vapour deposition, spray pyrolysis deposition (Carp, Huisman *et al.* 2004). In the preliminary product screening by Sørensen *et al.* (2014), a total of 35 products that apply photo-reactive titanium dioxide particles were identified.

Air cleaners - A limited number of air cleaners using artificial UV light and titanium dioxide as a catalyst for the removal of pathogens, viruses and volatile organic compounds are marketed. Most of the air cleaners have one or more pre-filtration steps before the air is passed over the titanium dioxide-coated surface.

2.2.1 Manufacturing and import/export of the substance on its own

**Manufacturing processes**	The overall description the manufacturing of tiO2 is included in section 2.1.1.		
**Manufacturing in Denmark**	Nanosized TiO_2 is not manufactured in Denmark		
**Name of parameter**	**Unit**	**Value**	**Remark, data source**
**Import of the substance on its own uses to Denmark**	t/year	1-10	
**Re-export**	% of import		No export of the substance on its own has been identified

2.2.2 Formulation in Denmark

**Photocatalytic titanium dioxide (TiO_2)**			
**Identified formulation processes in Denmark**	The use of photoactive TiO2 for the manufacture of paint in Denmark has been confirmed by Sørensen *et al.* (2014). The photoactive TiO2 is among others used for protection against microbial growth on roof paints.		
**Name of parameter**	**Unit**	**Value**	**Remark, data source**
**Formulation 1: Production of paint and lacquers**			
**Number of companies**	companies	<4	
**Quantities used**	t/year	1-10	Exact figures not available. The quantities are roughly estimated.
**Ending up in final products**	%	97.5%	
**Release to municipal waste water system**<br>* Unless otherwise noted the release values were reduced/ enlarged on each side by 50% for the modeling of symmetrical triangular distributions around the specified quantities. The symmetry may possibly be by the absolute border values (highest or lowest possible release value, 1 and 0). In cases were more values are given, the mean is taken as modeal value for such distributions.	%	<0,5	The emission scenario document (ESD) for the paint industry from the OECD (2009) assume for manufacture of aqueous dispersion coatings that the total fraction of raw materials lost to waste from the manufactring process is 1.5%. This includes 1% lost due to residues in the mixing vessels and 0.5% due to residues in bags, spills and product returns. It is in the ESD assumed that half of the residue material in the mixing vessels will be re-used in the manufacturing process (recycling. For aqueous dispersion coatings the remaining equipment residue is assumed to be removed in water washings and hence to waste water.<br><br>According to information from Danish manufactures waste water originates from cleaning of tanks and other production equipment. Approximately 1-2% of the total used may be released to the waste water for pre-treatment at the manufacturing sites.<br><br>The first step at all sites is a precipitation/flocculation where the majority of the TiO2 is precipitated and ends up in a sludge/filter cake which is disposed of for external incineration or gasification.<br><br>The pretreated waste water is directed to municipal waste water plants.<br><br>As a worst case estimate, the releases to municipal waste water treatment plants are estimated to be <0,5%. The actual release is probably significantly below this value.
**Direct release to surface water (after internal WW treatment)**	%	0	No direct discharge to surface water
**Direct release to soil**	%	0	
**Direct release to air**	%	0	The TiO2 is imported as pastes in which the TiO2 is dispersed in water. The generation of dust by handling of the pastes is considered insignificant.
**Disposed of as solid waste for incineration**	%	2	Filtercake/sludge and TiO2 remaining in packaging are disposed of for incineration or gasification.
**Transformation during formulation into other forms**	%		Considered insignificant
**Percentage of produced products exported**	% of quantity in final product	No data	

Photocatalytic titanium dioxide (TiO_2)

2.2.3 Import/export and end-use in articles and mixtures

Identified uses in articles and mixtures

The distribution on end-uses of nano-TiO2 presented by Sun *et al.* (2014) is here divided into two groups: photostable and other applications of TiO2 (previous section) and photoactive TiO2 (this section).
Please note that the indicated percentages of the use of photoactive TiO2 shown below are percentage of the total consumption of nano-TiO2. The terms in the brackets indicated the terms used for the application areas in Sun *et al.* (2014)

	End use	Percentage of total TiO_2 consumption<br>Lower, modal , higher value<br>(l,m,h)
1	Paints	0, 8.9, 26
2	Coatings	0, 3.7, 19
3	Construction materials (Cement)	0, 0.1, 1
4	Waste water treatment	0, 0.7, 4
5	Cleaning of water and air (Filters)	0, 5.8, 26

**Name of parameter**	**Unit**	**Value**	**Remark, data source**
**End-use 1: Paints**			
**Total consumption**	% of total consump-tion of TiO_2	8.9	Percentage (mean value) of the total nano-TiO2 use (Sun *et al.*, 2014).<br>A part of the nano TiO2 used for paint may in fact be photostable TiO2 applied for UV protection.<br><br>The transfer coefficients used in the Swiss study has been reconsid-ered for the Danish situation as shown below
**Release to municipal waste water system**	%	1	Dust and flakes from maintenance of painted surfaces and from abrasion of painted surfaces
**Direct release to surface water**	%	1	Dust and flakes from maintenance of painted surfaces and from abrasion of painted surfaces
**Direct release to soil**	%	1	Dust and flakes from maintenance of painted surfaces and from abrasion of painted surfaces
**Direct release to air**	%	1	Dust and flakes from maintenance of painted surfaces and from abrasion of painted surfaces
**Disposed of to MSWI**	%		Paint remaining in packaging and paint on wood and other combus-tible materials
**Disposed of to landfill**	%	50	Paint on concrete and other non-combustible building materials
**Disposed of for recycling (excl. energy recovery)**	%	46	Paint on metals
**End-use 2: Coatings**			
**Total consumption**	% of total consum-ption of TiO_2	3.7	Percentage (mean value) of the total nano-TiO2 use (Sun *et al.*, 2014).
**Direct release to surface water**	%		[In the Danish situation probably a part will be discharged directly to the environment from areas with municipal separate storm water sewer systems]

**Photocatalytic titanium dioxide (TiO_2)**			
**Release to municipal waste water system**	%	90	
**Direct release to air**	%	5	
**Disposed of to MSWI**	%	5	
**End-use 3: Construction material (Cement)**			
**Total consumption**	% of total consump-tion of TiO_2	0.1	Percentage (mean value) of the total nano-TiO2 use (Sun *et al.*, 2014).
**Release to municipal waste water system**	%	1	
**Disposed of to landfill**	%	19	
**Disposed of for recycling (excl. energy recovery)**	%	80	
**End-use 4: Waste water treatment**			
**Total consumption**	% of total consump-tion of TiO_2	0.7	Percentage (mean value) of the total nano-TiO2 use (Sun *et al.*, 2014).
**Release to municipal waste water system**	%	95	[The information on the use of nano TiO2 for waste water treatment in the Danish survey indicates that the nano TiO2 has been deposited as a thin film and is not released to the waste water - Should be reconsidered]
**Disposed of to MSWI**	%	5	
**End-use 5: Cleaning of water and air (Filters)**			
**Total consumption**	% of total consump-tion of TiO_2	5.8	Percentage (mean value) of the total nano-TiO2 use (Sun *et al.*, 2014).
**Release to municipal waste water system**	%	25	(Sun *et al.*, 2014)
**Direct release to air**	%	5	(Sun *et al.*, 2014)
**Disposed of to MSWI**	%	70	(Sun *et al.*, 2014)

2.2.4 Waste water treatment

**Name of parameter**	**Unit**	**Value**	**Remark, data source**
**Transformation during STP treatment into other forms**	%	-	See indications for Photo-stable and other nanomaterial titanium dioxide (TiO2)
**Percentage ending up in sludge**	% (l, m, u)	-	See line above.
**Percentage discharges**	% (l, u)	-	See line above.

2.2.5 Solid waste treatment (incineration and landfill)

**Name of parameter**	**Unit**	**Value**	**Remark, data source**
**Transformation or deposition during incineration into other forms (average Danish MSWIs)**	%	approx. 0.1-52 (deposition, transformation)	See remarks on data and sources for phostostable nano-TiO2.

Photocatalytic titanium dioxide (TiO_2)			
**Percentage emitted to the air (average Danish MSWIs)**	%	~0	See line above.
**Percentage ending up in residues (average Danish MSWIs)**	%	approx. 36-75 (slag)<br>approx. 3-9 (fly ash)	See line above.
**Release from landfills to municipal waste water treatment**	kg/year	0	For landfill, no leachate out is assumed (Sun *et al.*, 2014).
**Direct release from landfills to surface water**	kg/year	0	See line above
**Transformation during landfilling into other forms**	%	No data	At this point we stopped our modelling. Nanomaterial fate and behaviour during landfilling was not considered. See also general comments on landfilling.
**2.2.6 Recycling**			
**Type of recycling activities**	No recycling activities identified		

2.3 Zinc oxide (ZnO)

**Zinc oxide (ZnO)**			
**General applications**			
Piccinno *et al.* (2012) report for nano-ZnO very similar (compared to nano-TiO_2) applications by listing all above paints, cosmetics and sun-screens. The cosmetic use is confirmed also by others (AZoNano, 2013) by mentioning popularly known calamine lotions containing zinc oxide powder and the use in ointments for treating skin diseases. Filter application in rubber and cigarettes and the application as an additive in the manufacture of concrete and zinc oxide powders for the Ceramic industry and as additive material for the food industry are also mentioned. Others (Steinfeldt *et al.*, 2013) focused on nano-ZnO application in glass coatings by stressing that this type of ENM would improve the optical material characteristics by reducing (as seen above) UV permeability while simultaneously increasing the visible transmittance. These authors also refer to others (Lowry *et al.*, 2008) that for their part emphasize the use in coatings due to material properties, such as being stable and nonmigratory within coating matrices, ending up in a longer service life for the target coated products. Coating agent use is also mentioned for paints (AZoNano, 2013). A promising future field of applications has been demonstrated for optoelectronics, sensors, transducers and biomedical sciences due to the fact that such ZnO is as seen above a multi-functional material and due to its high variety of growth morphologies, such as nanocombs, nanorings, nanohelixes/nanosprings,nanobelts, nanowires and nanocages (Wang, 2004). Wang (2004) pays particular attention to nanobelts as nanosensors, nanocantilevers, field effect transistors and Nanoresonators.<br><br>Nano-ZnO is according to the Cosmetics Regulation (Regulation (EC) No 1223/2009) not included in the list of UV filter allowed in cosmetic products in the EU and Denmark.			
**2.3.1 Manufacturing and import/export of the substance on its own**			
**Manufacturing processes**			
**Manufacturing in Denmark**	Nanosized zinc oxide is not produced in Denmark.		
**Name of parameter**	**Unit**	**Value**	**Remark, data source**
**Import of the substance on its own uses to Denmark**	kg/year		
**Re-export**	% of import		
**2.3.2 Formulation in Denmark**			
**Identified formulation processes in Denmark**	No formulation processes involving nanosized zinc oxide in Denmark have been identified.		
**2.3.3 Import/export and end-use in articles and mixtures**			

**Zinc oxide (ZnO)**	
**Identified uses in articles and mixtures**	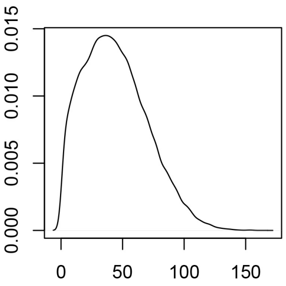 Figure 4. Annual nano-ZnO use in Denmark (t/a).<br><br>Total use of the substance in articles and mixtures in Denmark is estimated at 0.6-165 t/year (minimal and maximal value used in our MC based mass flow model. The figure is adapted from Swiss values declared to show different levels of reliability expressed as degree of belief (DoB) of 80% and 20% (Sun *et al.*, 2014) and based on a comparison of the population numbers Denmark-Switzerland.<br><br>According to Sun *et al.* (2014) the DoB was considered as a combination of Bayes inference and Monte Carlo calculations in a Markov Chain Monte Carlo (MCMC) approach by computing more or fewer values (in relation to their DoB) based on each corresponding rough input production value. 80% reliability was attributed to 70 t/y (Schmid and Riediker, 2008) and to the range between 0.2 and 911 t/y (Piccinno *et al.*, 2012), 20% reliability used as prior knowledge to 0.15 (Nightingale *et al.*, 2008), 4.4 (Zhang and Saebfar, 2010) and 84 t/y (Aschberger *et al.*, 2011).<br>In our model these raw values for total use valid for Switzerland were scaled down data from other regions (Switzerland; Europe and global dimensions) into Danish levels based on the comparison of the population numbers.<br><br>Sun *et al.* (2014) summarize current available quantitative estimations: Nightingale *et al.* (2008) suggest global 18 t/y nano-ZnO production, Zhang and Saebfar (2010) reported 528 t/y; others (Aschberger *et al.*, 2011) suggest 10,000 t/y. Another group (Schmid and Riediker, 2008) suggests 70 t/y for the Swiss nano-ZnO production/use; European results of 5.5 to 28,000 t/y were reported as well (Piccinno *et al.*, 2012).<br><br>In a further step the total annual use volume was allocated to end use categories. Such categories integrate ENM containing products with similar material life-cycles characteristics where we could assume identical environmental release kinetics

**Zinc oxide (ZnO)**			
	The computed ENM fraction in each category reflects as described in detail in Gottschalk *et al.* (2009) the counted number of products per category multiplied by the assumed share of the overall distributed ENM mass in each product.<br><br>Lower, upper boundary and mean mass fraction of ENM modelled for the allocation to different product applications. Details on such computation from different sources are explained in detail below and exemplary for the nano-TiO2 and based on a recent study (Sun *et al.*, 2014). Such comprehensive data collection and preparation bases on information from the ANEC/BEUC Inventory (ANEC/BEUC, 2010), the nanomaterial market report (Future Markets, 2011), the Woodrow Wilson Centre inventory of the for Scholars' Project on Emerging Nanotechnologies (WWI, 2012), the BUND inventory (BUND, 2011) as well as a summary of nanotechnology patents (Lem *et al.*, 2012) and an own internet search using Google, Yahoo and EC21.		

	End use	Percentage of total<br>Lower, modal, higher value<br>(l,m,h)
1	Cosmetics	27, 83, 100
2	Paints	0, 14, 57
3	Filters	0, 0.1, 1
4	Consumer electronics	0, 0.2, 1
5	Plastics	0, 2, 11
6	Textiles	0, 0.01, 0.02
7	Paper	0, 0.02,0.14
8	Woods	0, 0.01, 0.04
9	Foods	0, 0, 0.01
10	Cleaning agents	0, 0.15, 1
11	Metals	0, 0, 0.02
12	Glass & ceramics	0, 0.7, 4

**Name of parameter**	**Unit**	**Value**	**Remark, data source**<br>Unless otherwise noted mean values used for triangular distributions with 50% deviation on each side that refer to Sun *et al.* (2014).
**End-use 1: Cosmetics**			
**Total consumption**	% of total consump-tion	83	Percentage (mean value) of the total nano-ZnO.<br>As mentioned elsewhere, nano-ZnO is not included in the list of UV filter allowed in cosmetic products in the EU and Denmark. Even some products may not be in compliance with the Regulation it seems unlikely that cosmetics should account for a majority of the use nano-ZnO.<br>The figures are kept here for the first round of computing as a worst case estimate.

**Zinc oxide (ZnO)**			
**Release* to municipal waste water system**<br>* Unless otherwise noted the re-lease values were reduced/ enlarged on each side by 50% for the model-ing of symmetrical triangular distributions around the specified quantities. The symmetry may possibly be by the absolute border values (highest or lowest possible release value, 1 and 0). In cases were more values are given, the mean is taken as modal value for such distributions.	%	75	
**Direct release to surface water**	%	10	
**Disposed of to MSWI**	%	5	
**Transformation during use into other forms**	%	10	Transformation in the form of dissolution during the use phase was considered as material elimination due to contact with water (Sun *et al.*, 2014).
**End-use 2: Paints**			
**Total consumption**	% of total consump-tion	14	Percentage (mean value) of the total nano-ZnO use (Sun *et al.*, 2014).
**Release to municipal waste water system**	%	1	
**Direct release to surface water**	%	1	
**Direct release to soil**	%	1	
**Direct release to air**	%	1	
**Disposed of to MSWI**	%		
**Disposed of to landfill**	%	50	Disposed off with non-combusible building materials
**Disposed of for recycling (excl. energy recovery)**	%	41	
**Transformation during use into other forms**	%	5	Transformation in the form of dissolution during the use phase was considered as material elimination due to contact with water (Sun *et al.*, 2014).
**End-use 3: Filters**			
**Total consumption**	% of total consump-tion	0.1	Percentage (mean value) of the total nano-ZnO use (Sun *et al.*, 2014).
**Release to municipal waste water system**	%	20	
**Direct release to air**	%	5	
**Disposed of to MSWI**	%	70	

**Zinc oxide (ZnO)**			
**Transformation during use into other forms**	%	5	
**End-use 4: Consumer Electronics**			
**Total consumption**	% of total consump-tion	0.2	Percentage (mean value) of the total nano-ZnO use (Sun *et al.*, 2014).
**Disposed of to MSWI**	%	5	
**Disposed of for recycling (excl. energy recovery)**	%	75	
**Transformation during use into other forms**	%	20	
**End-use 5: Plastics**			
**Total consumption**	% of total consump-tion	2	Percentage (mean value) of the total nano-ZnO use (Sun *et al.*, 2014).
**Disposed of to MSWI**	%	100	
**End-use 6: Textiles**			
**Total consumption**	% of total consump-tion	0.01	Percentage (mean value) of the total nano-ZnO use (Sun *et al.*, 2014).
**Release to municipal waste water system**	%	1	
**Direct release to air**	%	1	
**Disposed of to MSWI**	%	58	
**Transformation during use into other forms**	%	30	
**Export**		30	
**End-use 7: Paper**			
**Total consumption**	% of total consump-tion	0.02	Percentage (mean value) of the total nano-ZnO use (Sun *et al.*, 2014).
**Disposed of to MSWI**	%	25	
**Disposed of for recycling (excl. energy recovery)**	%	70	
**Export**	%	10	
**End-use 8: Woods**			
**Total consumption**	% of total consump-tion	0.01	Percentage (mean value) of the total nano-ZnO use (Sun *et al.*, 2014).
**Release to municipal waste water system**	%	1	
**Disposed of to MSWI**	%	94	
**Transformation during use into other forms and release to air**	%	5	Transformation in the form of dissolution during the use phase was considered as material elimination due to contact with water (Sun *et al.*, 2014).
**End-use 9: Foods**			

**Zinc oxide (ZnO)**			
**Total consumption**	% of total consump-tion	<0.01	Percentage (mean value) of the total nano-ZnO use (Sun *et al.*, 2014).
**Transformation during use into other forms**	%	100	Complete dissolution is considered due to contact with gastric acid in stomach (Sun *et al.*, 2014).
**End-use 10: Cleaning agent**			
**Total consumption**	% of total consump-tion	0.15	Percentage (mean value) of the total nano-ZnO use (Sun *et al.*, 2014).
**Release to municipal waste water system**	%	90	
**Direct release to air**	%	5	
**Disposed of to MSWI**	%	5	
**End-use 11: Metals**			
**Total consumption**	% of total consump-tion	<0.02	Percentage (mean value) of the total nano-ZnO use (Sun *et al.*, 2014).
**Release to municipal waste water system**	%	5	
**Disposed of to MSWI**	%	5	
**Disposed of for recycling (excl. energy recovery)**	%	90	
**Transformation during use into other forms**	%		
**End-use 12: Glass & ceramics**			
**Total consumption**	% of total consump-tion	0.7	
**Release to municipal waste water system**	%	1	
**Direct release to air**	%		
**Disposed of to MSWI**	%	20	
**Disposed of to landfill**	%		
**Disposed of for recycling (excl. energy recovery)**	%	74	
**Transformation during use into other forms**	%	5	
**Export**	%		
**2.3.4 Waste water treatment**			
**Name of parameter**	**Unit**	**Value**	**Remark, data source**
**Transformation during STP treatment into other forms**	%	100	(Lombi *et al.*, 2012) reported on the fate and behaviour of zinc oxide nanosized particles during anaerobic digestion in wastewater and post-treatment processes of sewage sludge. These authors concluded that after such treatments the target particles were transformed into non-nanomaterial forms.

**Zinc oxide (ZnO)**			
**Percentage ending up in sludge**	%	-	See comments above.
**Percentage discharges**	%	-	See comments above.
**2.3.5 Solid waste treatment (incineration and landfill)**			
**Name of parameter**	**Unit**	**Value**	**Remark, data source**
**Transformation or deposition during incineration into other forms (average Danish MSWIs)**	%	approx. 0.1-52 (deposition, transformation)	Mass transfer and fate parameters (see please Figure 1) for all metallic ENMs in a waste incineration system are modelled as shown below in Table 1 that reflects the values derived from computer based simulations that were combined with real analytic/experimental results of a detailed nano-CeO_2 case study (Walser and Gottschalk, 2014). These results show the steady state mass transport/transformation for all relevant WIP paths reached after steady state mode of such plants (infinite time scale). This means that analytically not detected and not further transported material mass has been assigned to the subsequent further transport and/or to the subsequent deposition/transformation by covering at each stage in the WIP process the entire range of transport and fate possibilities. See also comments on the nano-TiO_2 cases.
**Percentage emitted to the air (average Danish MSWIs)**	%	~0	See line above.
**Percentage ending up in residues (average Danish MSWIs)**	%	approx. 36-75 (slag) approx. 3-9 (fly ash)	See line above.
**Release from landfills to municipal waste water treatment**	kg/year	0	For landfill, no leachate out is assumed (Sun *et al.*, 2014).
**Direct release from landfills to surface water**	kg/year	0	See line above.
**Transformation during landfilling into other forms**	%	No data	At this point we stopped our modelling. Nanomaterial fate and behaviour during landfilling was not considered. See also general comments on landfilling.
**2.3.6 Recycling**			
**Type of recycling activities**	**description**		
**Name of parameter**	**Unit**	**Value**	**Remark, data source**
**Transformation during recycling into other forms**	**%**	No data	Currently quantitative information that could be used to model fate and behaviour of ENM during and after recycling is not available. We did not track the material fate and mass flows of the studied nanoparticles during and after the recycling process. See also comments on the previus cases.
**Ending up in recycled products**	**%**	~20	Most part directly after product use. Bottom ash is e.g. recycled up to 100 % in road construction, soil consolidation and anti-frost layers under buildings. A few percent are landfilled.
**Release from recycling process**	**% of recycled**	0	See lines above.

2.4 Silver (AgNP)

**Silver (Ag)**			
**General applications**			
The use of AgNP is very diverse and include therapeutic applications (diet supplement), personal care products, powdered colours, varnish, textile, paper, interior and exterior paints, printing colours, water and air-purification, polymer-based products and foils for antibacterial protection such as washing machines, kitchenware and food storage. The AgNP concentrations used are unknown for most applications. The scale of use of AgNP is unknown at this point in time, but expected to increase rapidly as more and more consumer products with AgNP are entering the market. (Mikkelsen *et al.*, 2011)<br>In some types of applications, the AgNP is dispersed in mixture (e.g. paint or printing colours) whereas in others it may be adhered to a surface (e.g. in textiles), or it may be embedded in a polymer matrix as in hygienic surfaces of kitchenware and equipment for food storage.			
**2.4.1 Manufacturing and import/export of the substance on its own**			
**Manufacturing processes**	Ultra-sonic precipitation, chemical vapour deposition, exploding wire synthesis. The size, shape, surface area, etc. can be modified by adding various surface active agents and coatings to syntheses involving silver salts.		
**Manufacturing in Denmark**	AgNP is not manufactured in Denmark		
**Name of parameter**	**Unit**	**Value**	**Remark, data source**
**Import of the substance on its own uses to Denmark**	kg/year	No data indentified	
**Re-export**	% of import	No data indentified	
**2.4.2 Formulation in Denmark**			
**Identified formulation processes in Denmark**	No formulation processes with the use of AgNP in Denmark have been identified.		
**2.4.3 Import/export and end-use in articles and mixtures**			

<table>
<tr><th colspan="2">Silver (Ag)</th></tr>
<tr><td>Identified uses in articles and mixtures</td><td>
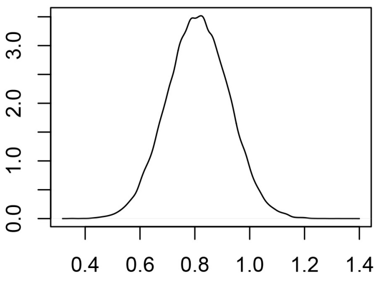

Figure 5. Annual nano-Ag use in Denmark (t/a).

Total Danish use of nanao-Ag in articles and mixtures is modelled between x-x t/year (minimal and maximal value used in the MC model).
The figure 5 reflects data of different levels of reliability modeled as degree of belief (DoB) of 80% and 20% (Sun et al., 2014) considered as a combination of Bayes inference and Monte Carlo calculations by computing more or fewer values (in relation to their DoB) and by comparing the population numbers Denmark-Switzerland.

For nano-Ag annual global productions/use volumes were presented from different groups (Sahasrabudhe, 2010; Scheringer et al., 2010; Aschberger et al., 2011; Piccinno et al., 2012; Windler et al., 2013). The raw data of use volume for Switzerland (that have been scaled down for Denmark approx. with a factor of 0.7)) with Degree of Belief as reported in detail in Sun et al. (2014): 80% reliability was attributed to values ranging from 0.02 t annual use (Piccinno et al., 2012) to 3 t annual use (Schmid and Riediker, 2008). 20% reliability was attributed to values ranging from 0.36 t/a (Blaser et al., 2008) to 4.2 t/a (Aschberger et al., 2011)

As shown in figure 5 the modelled final use of the substance in Denmark is assumed to range approx. from 0.3-1.4 t/a with modal values around 0.8 t/a.

In an additional step the total annual use volume was linked to end use categories.
Such categories integrate ENM containing products with similar material life-cycles properties leading to (assumed) identical environmental release characteristics.

Lower, upper boundary and mean mass fraction of ENM modelled for the allocation to different product applications. Details on such computation from different sources are explained in detail below and exemplary for the AnNP and based on a recent study (Sun et al., 2014).
</td></tr>
</table>

Silver (Ag)

	End use	Percentage of total<br>Lower, mean, higher value<br>(l,m,h)
1	Textiles	15, 25, 35
2	Cleaning agents	2, 6, 15
3	Paints	0, 3, 15
4	Consumer electronics	11, 38, 60
5	Cosmetics	4, 10, 31
6	Medtech	0, 4, 15
7	Plastics	0, 3, 7
8	Food	0, 7, 24
9	Glass & ceramics	0, 1, 4
10	Metals	0, 2, 12
11	Soil remediation	0, 1, 4
12	Filtration	0, 0.3, 0.6
13	Sanitary	0, 0.16, 0.5
14	Paper	0, 0.1, 1

**Name of parameter**	**Unit**	**Value**	**Remark, data source**
**End-use 1: Textiles**			
**Total consumption**	%	25	Percentage (mean value) of the total nano-TiO2 use (Sun *et al.*, 2014).
**Release to municipal waste water system**<br>* Unless otherwise noted the release values were reduced/ enlarged on each side by 50% for the modeling of symmetrical triangular distributions around the specified quantities. The symmetry may possibly be by the absolute border values (highest or lowest possible release value, 1 and 0). In cases were more values are given, the mean is taken as modal value for such distributions.	%	32	Derived from empirical data as suggested by others (Sun *et al.*, 2014)
**Direct release to air**	%	5	
**Disposed of to MSWI**	%	32	
**Disposed of for recycling (excl. energy recovery)**	%	6.4	
**Transformation during use into other forms**	%	5	
**Export**	%	25.6	
**End-use 2: Cleaning agent**			

**Silver (Ag)**			
**Total consumption**	%	6	Percentage (mean value) of the total nano-TiO2 use (Sun *et al.*, 2014).
**Release to municipal waste water system**	%	85	
**Direct release to air**	%	5	
**Disposed of to MSWI**	%	5	
**Transformation during use into other forms**	%	5	Transformation in the form of dissolution during the use phase was considered as material elimination due to contact with water (Sun *et al.*, 2014).
**End-use 3: Paints**			
**Total consumption**	%	3	Percentage (mean value) of the total nano-TiO2 use (Sun *et al.*, 2014).
**Release to municipal waste water system**	%	1	
**Direct release to surface water**	%	1	
**Direct release to soil**	%	1	
**Direct release to air**	%	1	
**Disposed of to landfill**	%	50	
**Disposed of for recycling (excl. energy recovery)**	%	41	
**Transformation during use into other forms**	%	5	Transformation in the form of dissolution during the use phase was considered as material elimination due to contact with water (Sun *et al.*, 2014).
**End-use 4: Consumer electronics**			
**Total consumption**	%	38	Percentage (mean value) of the total nano-TiO2 use (Sun *et al.*, 2014).
**Release to municipal waste water system**	%		
**Disposed of to MSWI**	%	5	
**Disposed of for recycling (excl. energy recovery)**	%	75	
**Export**	%	20	
**End-use 5: Cosmetics**			
**Total consumption**	%	10	Percentage (mean value) of the total nano-TiO2 use (Sun *et al.*, 2014).
**Release to municipal waste water system**	%	80	

**Silver (Ag)**			
**Direct release to surface water**	%	10	
**Disposed of to MSWI**	%	5	
**Transformation during use into other forms**	%	5	
**End-use 6: Medtech**			
**Total consumption**	%	4	Percentage (mean value) of the total nano-Ag use (Sun *et al.*, 2014).
**Release to municipal waste water system**	%	5	
**Disposed of to MSWI**	%	5	
**Disposed of for recycling (excl. energy recovery)**	%	90	
**Transformation during use into other forms**	%	0	
**End-use 7: Plastics**			
**Total consumption**	%	3	Percentage (mean value) of the total nano-TiO2 use (Sun *et al.*, 2014).
**Disposed of to MSWI**	%	95	
**Transformation during use into other forms**	%	5	Transformation in the form of dissolution during the use phase was considered as material elimination due to contact with water (Sun *et al.*, 2014). Such dissolution was modelled based on data presented in Blaser *et al.* (2008) when studying the release of Ag from biocidal plastics. Sun *et al.* (2014) did not assume complete dissolution of Ag for natural waters, although suggesting that a continuous dissolution was in principle possible, and that the particle form could persist sufficiently long for allowing new pathways of silver partitioning and mass transfer.
**End-use 8: Food**			
**Total consumption**	%	7	Percentage (mean value) of the total nano-TiO2 use (Sun *et al.*, 2014).
**Release to municipal waste water system**	%	90	
**Disposed of to MSWI**	%	10	
**End-use 9: Glass & ceramics**			
**Total consumption**	%	1	Percentage (mean value) of the total nano-TiO2 use (Sun *et al.*, 2014).
**Release to municipal waste water system**	%	1	
**Disposed of to MSWI**	%	20	
**Disposed of for recycling (excl. energy recovery)**	%	74	
**Transformation during use into other forms**	%	5	Transformation in the form of dissolution during the use phase was considered as material elimination due to contact with water (Sun *et al.*, 2014).

**Silver (Ag)**			
**End-use 10: Metals**			
**Total consumption**	%	2	Percentage (mean value) of the total nano-TiO2 use (Sun *et al.*, 2014).
**Release to municipal waste water system**	%	5	
**Disposed of to MSWI**	%	5	
**Disposed of for recycling (excl. energy recovery)**	%	90	
**End-use 11: Filtration**			
**Total consumption**	%	0.3	Percentage (mean value) of the total nano-TiO2 use (Sun *et al.*, 2014)
**Release to municipal waste water system**	%	25	
**Direct release to air**	%	5	
**Disposed of to MSWI**	%	60	
**Transformation during use into other forms**	%	10	Transformation in the form of dissolution during the use phase was considered as material elimination due to contact with water (Sun *et al.*, 2014).
**End-use 12: Sanitary**			
**Total consumption**	%	0.16	Percentage (mean value) of the total nano-TiO2 use (Sun *et al.*, 2014).
**Release to municipal waste water system**	%	5	
**Direct release to surface water**	%		
**Direct release to soil**	%		
**Direct release to air**	%		
**Disposed of to MSWI**	%	95	
**Disposed of to landfill**	%		
**Disposed of for recycling (excl. energy recovery)**	%		
**Transformation during use into other forms**	%		
**Export**	%		
**End-use 13: Paper**			
**Total consumption**	%	0.1	Percentage (mean value) of the total nano-TiO2 use (Sun *et al.*, 2014).
**Release to municipal waste water system**	%		
**Direct release to surface water**	%		
**Direct release to soil**	%		

Silver (Ag)			
**Direct release to air**	%		
**Disposed of to MSWI**	%	10	
**Disposed of to landfill**	%	16	
**Disposed of for recycling (excl. energy recovery)**	%	63	
**Transformation during use into other forms**	%		
**Export**	%	10	
**2.4.4 Waste water treatment**			
**Name of parameter**	**Unit**	**Value**	**Remark, data source**
**Transformation during STP treatment into other forms**	%	85-100	85%-100% of the metallic nano-Ag was reported to be transformed into Ag_2S (Kaegi *et al.*, 2011), which means only less than 15% of initial nano-Ag entering into STP with wastewater can survive STP process.
**Percentage ending up in sludge**	%	Approx. 0-100	As seen above empirical distributions with mean, an the lower and upper limit values as indicated in the column on the left that follow the newest evidence (Sun *et al.*, 2014) were modeled. These values reflect data taken from different sources (Kiser *et al.*, 2010; Tiede *et al.*, 2010; Kaegi *et al.*, 2011; Hou *et al.*, 2012; Wang *et al.*, 2012). 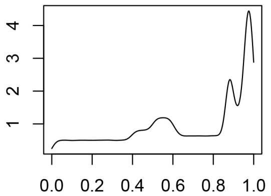 Figure 6. Modelled probability distribution of STP removal efficiency for nano-Ag (Sun *et al.*, 2014) See please for detailed model procedure explanations the equivalent information on the nano-TiO2 case.
**Percentage discharges**	%	0-15	See line above.
**2.4.5 Solid waste treatment (incineration and landfill)**			
**Name of parameter**	**Unit**	**Value**	**Remark, data source**
**Transformation during incineration into other forms (average Danish MSWIs)**	%	approx. 0.1-52 (deposition, transformation)	Sources of data and all technical details on the processes of waste incineration and landfilling correspond to the ones reported for other metallic nanoparticles (Walser and Gottschalk, 2014). See also comments on the previous nano-ZnO case.
**Percentage emitted to the air (average Danish MSWIs)**	%	~0	See line above.
**Percentage ending up in residues (average Danish MSWIs)**	%	approx. 36-75 (slag) approx. 3-9 (fly ash)	See line above.

Silver (Ag)			
**Release from landfills to municipal waste water treatment**	kg/year	0	For landfill, no leachate out is assumed, see also comments on previus cases.
**Direct release from landfills to surface water**	kg/year	0	See line above.
**Transformation during landfilling into other forms**	%	No data	At this point we stopped our modelling. Nanomaterial fate and behaviour during landfilling was not considered. See also general comments on landfilling.
**2.4.6 Recycling**			
**Type of recycling activities**	Recycling of bottom ash from waste incineration		
**Name of parameter**	**Unit**	**Value**	**Remark, data source**
**Transformation during recycling into other forms**	**%**	Not considered in the model.	See general comments on recycling processes.
**Ending up in recycled products**	**%**	Not considered in the model.	Most part directly after product use. Bottom ash is e.g. recycled up to 100 % in road construction, soil consolidation and anti-frost layers under buildings. A few percent are landfilled.
**Release from recycling process**	**% of recycled**	0	See lines above.

2.5 Carbon nanotubes (CNT)

<table>
<tr><td colspan="4">Carbon nanotubes (CNT)</td></tr>
<tr><td colspan="4">2.5.1 General description</td></tr>
<tr><td colspan="4">General applications</td></tr>
<tr><td colspan="4">Steinfeldt et al. (2013) emphasized a widespread usage due to properties such as being persistent against degradation, or CNT as composite material (Ma et al., 2010) due to outstanding mechanical properties made perfect by multi-functional properties based e.g. on thermal and electrical conductivity (Bokobza, 2007; Gibson et al., 2007; Hu et al., 2006; Tsu-Wei et al., 2010). Survey results of (Piccinno et al., 2012) show that currently most material is probably used in Composites & polymer additives and Batteries. This includes probably flat panel displays, super composite fibres, and conductive plastics, field storage batteries, micro-electronics based on semiconductors and other conductive material (READE, 2013). Future applications are expected in a very broad (probably the widest one of all studied materials in this work) spectrum, READE (2013) list among other: ‚nano-lithography/-tweezers/-balance/-doping, data storage, magnetic nanotube, nano gear, nanotube actuator, molecular quantum wires, hydrogen storage, noble radioactive gas storage, solar storage, waste recycling, electromagnetic shielding, dialysis filters, thermal protection, reinforcement of armour and other materials, avionics, collision-protection materials, fly wheels, body armour and other.</td></tr>
<tr><td colspan="4">2.5.2 Manufacturing and import/export of the substance on its own</td></tr>
<tr><td>Manufacturing processes</td><td colspan="3"></td></tr>
<tr><td>Manufacturing in Denmark</td><td colspan="3"></td></tr>
<tr><td>Name of parameter</td><td>Unit</td><td>Value</td><td>Remark, data source</td></tr>
<tr><td>Import of the substance on its own uses to Denmark</td><td>kg/year</td><td></td><td></td></tr>
<tr><td>Re-export</td><td>% of import</td><td></td><td></td></tr>
<tr><td colspan="4">2.5.3 Down stream use of CNT for production proceses in Denmark</td></tr>
<tr><td>Identified formulation processes in Denmark</td><td colspan="3">No information on actual use of carbon nanotubes in production processes in Denmark has been identified. In the Nanoplast project "Nano-technological materials and products in the plastics industry: Exposure assessment and toxicological properties" published in 2012, the exposure to CNTs in the production of fibre-reinforced polymer nano composites was studied (Clausen et al., 2012). The study mentions the large potential of the use of CNTs in the composite industry but no actual large scale uses.</td></tr>
<tr><td>Name of parameter</td><td>Unit</td><td>Value</td><td>Remark, data source</td></tr>
<tr><td colspan="4">2.5.4 Import/export and end-use in articles and mixtures</td></tr>
</table>

Carbon nanotubes (CNT)

Identified uses in articles and mixtures

Total annual use of the substance in Denmark is modelled from approx. 1-18 t/y. The figures are adapted from Swiss (European) values declared to show varying reliability expressed as degree of belief of 80%and 20% (Sun *et al.*, 2014) on a comparison of the population numbers Denmark-Switzerland.

Sun *et al.* (2014) report current available quantitative estimations for CNT: global production/use: 55 - 3’300 t/y (Healy *et al.*, 2008; Aschberger *et al.*, 2011; Future Markets, 2011; Piccinno *et al.*, 2012). Our values leading to anormal use volume distribution reflect different estimations (80% reliability) (Schmid and Riediker, 2008; Future Markets, 2011; Hendren *et al.*, 2011; Piccinno *et al.*, 2012) and (20% reliability) (Healy *et al.*, 2008; Ray *et al.*, 2009; Aschberger *et al.*, 2011).

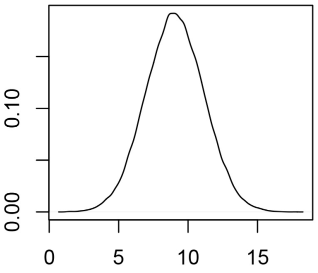

Figure 7. Annual use volumes computed for Denmark.

Please note that Research and Development (R&D) could be significant, but this is not considered an end use, and would be included under the use of the carbon nanotubes for formulation/production processes. Some R&D on the use of CNT has been undertaken in Denmark, among these the Nanoplast project "Nano-technological materials and products in the plastics industry: Exposure assessment and toxicological properties" (Clausen *et al.*, 2012). The environmental releases from the R&D activities are assumed to be insignificant and no attempt has been done to estimate the potential releases of CNT from R&D in Denmark.

Lower, upper boundary and mean mass fraction of ENM modelled for the allocation to different product applications. Details on such computation from different sources are explained in detail below and exemplary for the CNT and based on a recent study (Sun *et al.*, 2014)

	End use	Percentage of total<br>Lower, modal, higher value<br>(l,m,h)
1	Polymer composites	25 ,84, 1oo
2	Paints	0, 1, 10
3	Textiles	0, 0.02, 0.07
4	Automotive	0, 1, 10
5	Consumer electronics	0, 3, 24
6	Energy	0, 9, 50
7	Sensor	0, 0.4, 3
8	Aerospace	0, 0.6, 5

**Name of parameter**	**Unit**	**Value**	**Remark, data source**
**End-use 1: Polymer composites**			
**Total consumption**	%	84	Percentage (mean value) of the total nano-TiO2 use (Sun *et al.*, 2014).

**Carbon nanotubes (CNT)**			
**Direct release* to air**	%	1	* Unless otherwise noted the release values were reduced/ enlarged on each side by 50% for the modeling of symmetrical triangular distributions around the specified quantities. The symmetry may possibly be by the absolute border values (highest or lowest possible release value, 1 and 0). In cases were more values are given, the mean is taken as modal value for such distributions.
**Disposed of to MSWI**	%	99	
**Transformation during use into other forms**	%		For all uses (and the uses listed below) no dissolution or transformation/elimination during use and production was considered (Gottschalk *et al.*, 2009; Sun *et al.*, 2014).
**End-use 2: Paints**			
**Total consumption**	%	1	Percentage (mean value) of the total nano-TiO2 use (Sun *et al.*, 2014).
**Release to municipal waste water system**	%	1	
**Direct release to surface water**	%	1	
**Direct release to soil**	%	1	
**Direct release to air**	%	1	
**Disposed of to landfill**	%	50	
**Disposed of for recycling (excl. energy recovery)**	%	46	
**End-use 3: Textiles**			
**Total consumption**	%	0.02	Percentage (mean value) of the total nano-TiO2 use (Sun *et al.*, 2014).
**Release to municipal waste water system**	%	2	
**Direct release to air**	%	2	
**Disposed of to MSWI**	%	96	
**End-use 4: Automotive**			
**Total consumption**	%	1	Percentage (mean value) of the total nano-TiO2 use (Sun *et al.*, 2014).
**Direct release to air**	%	1	
**Disposed of to MSWI**	%	39	
**Disposed of for recycling (excl. energy recovery)**	%	40	
**Export**	%	20	
**End-use 5: Consumer electronics**			
**Total consumption**	%	3	Percentage (mean value) of the total nano-TiO2 use (Sun *et al.*, 2014).
**Disposed of to MSWI**	%	5	

**Carbon nanotubes (CNT)**			
**Disposed of for recycling (excl. energy recovery)**	%	75	
**Export**	%	20	
**End-use 6: Energy**			
**Total consumption**	%	9	Percentage (mean value) of the total nano-TiO2 use (Sun *et al.*, 2014).
**Disposed of to MSWI**	%	5	
**Disposed of for recycling (excl. energy recovery)**	%	75	
**Export**	%	20	
**End-use 7: Sensor**			
**Total consumption**	%	0.4	Percentage (mean value) of the total nano-TiO2 use (Sun *et al.*, 2014).
**Disposed of to MSWI**	%	5	
**Disposed of for recycling (excl. energy recovery)**	%	75	
**Export**	%	20	
**End-use 8: Aerospace**			
**Total consumption**	%	0.6	
**Disposed of to MSWI**	%	39	
**Disposed of for recycling (excl. energy recovery)**	%	60	
**Direct release to air**	%	1	
**Export**	%		
**2.5.5 Waste water treatment**			
**Name of parameter**	**Unit**	**Value**	**Remark, data source**
**Transformation during STP treatment into other forms**	%	0	
**Percentage ending up in sludge**	% (l,m,u)	0, 88 , 100	According to some evidence for Fullerenes used in Sun *et al.* (2014) we also base the computations on different Fullerene studies (Kiser *et al.*, 2010; Kiser *et al.*, 2012; Wang *et al.*, 2012).<br>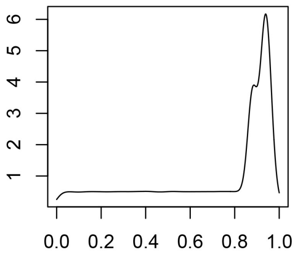<br><br>Figure 8. Removal of Fullerene into sewage treatment sludge.
**Percentage discharges**	% (l,m,u)	0,12,100	See line above.
**2.5.6 Solid waste treatment (incineration and landfill)**			

**Carbon nanotubes (CNT)**			
**Name of parameter**	**Unit**	**Value**	**Remark, data source**
**Transformation during incineration into other forms (average Danish MSWIs)**	% (l,m,u)	75, 98, 100	As suggested by others (Sun *et al.*, 2014) and in accordance to (Mueller *et al.*, 2013).
**Percentage emitted to the air (average Danish MSWIs)**	%	0.05, 0.1, 0.15	See line above.
**Percentage ending up in residues (average Danish MSWIs)**	%	40, 81, 100 (bootom ash)<br><br>0,19,60 (fly ash)	See line above.
**Release from landfills to municipal waste water treatment**	kg/year	0	For landfill, no leachate out is assumed, see also comments on previus cases.
**Direct release from landfills to surface water**	kg/year	0	See line above.
**Transformation during landfilling into other forms**	%	No data	At this point we stopped our modelling. Nanomaterial fate and behaviour during landfilling was not considered. See also general comments on landfilling.
**2.5.7 Recycling**			
**Type of recycling activities**	No relevant recycling activities identified		

2.6 CuCO3

Copper (Cu)

General applications

Copper carbonate - Micronized particles of cobber carbonate are used as wood preservative as alternative to other copper-based wood preservatives.

The Nanodatabase of the Danish Consumer Council (Tænk/Forbrugerrådet) include two products with nano copper, where links still exist:

- MesoCopper® - Nanoparticle Colloidal Copper: a mineral supplement in the form of a copper colloid consisting of nanometer particles of 0.9999 pure copper suspended in pure deionized water.

- DS Laboratories Revita.COR Hair-Growth Conditioner with nano-copper peptides.

Very limited information on the quantites of nanosized copper oxide and elemental copper has been available, and the modelling will consequently focus on the use of copper carbonate for wood treatment because 1) quantitative data are available for estimating the potential consumption in Denmark and 2) the quatities potentially used and releases to the environment will be significant.

2.6.1 Manufacturing and import/export of the substance on its own

Manufacturing processes	**Cobber carbonate** - Micronized particles of cobber carbonate are produced by mechanical grinding of water or oil-insoluble copper compounds with aid of dispersing/wetting agents in a carrier using a commercial grinding mill or by chemical means resulting in 90% or more of the particles being less than 1000 nm size. The commonly used carrier is water, and commonly used dispersing agents are polymeric dispersants, which attach to the surface of particles and keep the particles away from each other. Also, the presence of dispersing/wetting agents improves particle size reduction during milling and stabilizes the particles during storage and treating.
**Manufacturing in Denmark**	Micronized particles of cobber carbonate are not manufactured in Denmark.<br>Nanosized copper oxides are not manufactured in Denmark

**Name of parameter**	**Unit**	**Value**	**Remark, data source**
**Import of the substance on its own uses to Denmark**	kg/year	no data – expected to be zero	According to industry contact it is expected that micronized copper carbonated will be imported as a concentrate
**Re-export**	% of import	no data	

2.6.2 Formulation and industrial uses in Denmark

Identified formulation processes in Denmark	**Copper carbonate** -According to the Danish Pesticide Statistics, 91 tonnes of copper carbonate was used as active substances in wood preservatives in Denmark in 2011 whereas the consumption was 63.5 tonnes in 2009 and 84.5 tonnes in 2010 (DEPA, 2012). According to industry contacts (Osmose, 2013) none of the copper carbonate in 2012 was of the micronized type, but potentially in the future all the used copper carbonate could be of this type – resulting in a consumption of the same magnitude as the present use. In the USA, the market penetration of the new technology is about 75-80% (Osmose 2013).<br><br>It is expected that the formulation of concentrates used by the impregnation companies will take place abroad. By the formulation the micronized copper carbonate is mixed with a solvent. This concentrate is by the impregnation companies mixed with water. The concentrate is typically used in a 2% solution. In Denmark the impregnation of wood currently takes places in automatic closed processes according to BAT. It would be the same if the new technology is introduced.

Copper (Cu)			
	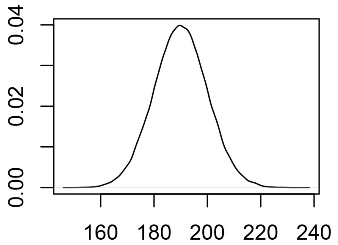<br>Figure 9. Annual use (in average 91 t for own formulation in Denmark and 100 t imported as part of impregnante material) in Denmark of copper carbonate for wood treatment.<br>Modelled normal distribution around a relative precise mean of 191 t/a (according to the Danish Pesticide Statistics) and a default standard deviation of 10.		
**Name of parameter**	**Unit**	**Value**	**Remark, data source**
**Formulation 1: Production of preserved wood (model estimates assuming all copper carbonate is of the micronized type)**			
**Number of companies**	companies	4	Number of companies for pressure impregnation members of Dansk Træbeskyttelse (Danish Wood impregnation)
**Quantities used**	tonnes/year	91	Assuming the consumption is on the 2011 level.
**Ending up in final products**	%	~99	Hansen *et al.*, 1997
**Release* to municipal waste water system**<br>* Unless otherwise noted the release values were reduced/ enlarged on each side by 50% for the modeling of symmetrical triangular distributions around the specified quantities. The symmetry may possibly be by the absolute border values (highest or lowest possible release value, 1 and 0). In cases were more values are given, the mean is taken as modal value for such distributions.	%	~0	According to Hansen *et al.* (1997) in the late 1990'es pressure impregnation took place in closed systems without any losses to waste water. Small releases could be due to leakages in the collection systems or by cleaning of working clothes and cleaning of filters.
**Direct release to surface water (after internal WW treatment)**	%	~0	
**Direct release to soil**	%	~0	
**Direct release to air**	%	~0	
**Disposed of as solid waste for incineration**	%	~0	
**Disposed of for other waste management**	%	~1	Small amounts of sludge disposed of as hazardous waste – not quantified in Hansen *et al.* (1997). Rough estimate.
**Transformation during use into other forms**	%	Data not available	
**Percentage of produced products exported**	% of quantity in final product	Data not available	
**2.6.3 Import/export and end-use in articles and mixtures**			

**Copper (Cu)**			
**Identified uses in articles and mixtures**	The micronised copper carbonate is present in pressure impregnated wood. The majority of the wood is various timber used for out-door applications		
**Name of parameter**	**Unit**	**Value**	**Remark, data source**
**End-use 1: Pressure impregnated wood**			
**Net-import with articles and mixtures**	Tons/year	100	(Osmose, 2013) assuming a potential market penetration of the new technology by 100%
**Total consumption**	Tons/year	190	The amount of impregnated wood in Denmark is about 250.000 m^3, about 50% imported and about 50% produced in Denmark (Osmose, 2013 based on data from the Danish Impregnation branch)
**Trend in consumption**	Unit-less	Stagnating	In the model it is anticipated that the micronized copper carbonate has a market penetration of 100%
**Average service life time**	year	30	Hansen *et al.*, 1997. The authors mentions that actual data are not available, but an average of 30 years is expected.
**Release to municipal waste water system**	%	1	Hansen *et al.*, 1997 estimate that 25% of copper from CCA (copper/chromium/arsenic) treated wood and 30-40% of the copper from CC (copper/chromium) treated wood during the life time. The releases from wood treated with micronized copper may be lower. The releases from wood below ground is higher than from wood above ground.<br>It is roughly assumed that the total is 30% - a minor part of this is released to surfaces with run off to urban sewage treatment plants and rain water run off directly to surface water<br>[Most probably the micronized copper is released as dissolved copper ions]
**Direct release to surface water**	%	1 (via urban rainwater run off)	
**Direct release to soil**	%	28	
**Direct release to air**	%	0	
**Disposed of to MSWI**	%	7%	According to current regulation pressure impregnated wood should be disposed of for landfill. A small part may be disposed of with non-impregnated wood for recycling (manufacture of wood-chip boards) and a part may be disposed of for incineration. For the current model estimates it is assumed that 80% (of the 70% remaining in the wood) is disposed of in accordance with the legislation and 10% to MSWI and recycling.
**Disposed of to landfill**	%	56%	
**Disposed of for recycling (excl. energy recovery)**	%	7%	
**Transformation during use into other forms**	%	0	Low levels of copper were detected at every stage of a leaching test of wood treated with micronized copper quat suggesting micronized copper may be capable of redistributing into cell walls. Copper-containing particles were detected in the lumens of micronized copper quat- and but were not present in untreated samples (Stirling *et al.*, 2008). While confirming the presence of copper in the cell wall was difficult, X-ray analysis indicated that there was a small amount of Cu in the cell walls in both Micronized copper quat- and ACQ-treated samples, and not in the untreated samples.<br>[most probably a major part of the micronized copper is transformed before release]
**2.6.4 Waste water treatment**			
**Name of parameter**	**Unit**	**Value**	**Remark, data source**

**Copper (Cu)**			
**Transformation during STP treatment into other forms**	%		No data
**Percentage ending up in sludge**	%	82	General values for copper in 1995 (Lassen *et al.*, 1996)
**Percentage discharges**	%	18	
**2.6.5 Solid waste treatment (incineration and landfill)**			
**Name of parameter**	**Unit**	**Value**	**Remark, data source**
**Transformation or deposition during incineration into other forms (average Danish MSWIs)**	%	approx. 0.1-52 (deposition, transformation)	Sources of data and all technical details on the processes of waste incineration and landfilling correspond to the ones reported for other metallic nanoparticles (Walser and Gottschalk, 2014). See also comments on the previous nano-Ag case.
**Percentage emitted to the air (average Danish MSWIs)**	%	~0	General values for copper in 1995 (Lassen *et al.*, 1996) were around 0.1 % and have been confirmed in the CeO2 study for metallic nanoparticles (Walser and Gottschalk, 2014). We used the model input values for waste incineration processes as done for other metals in our work.
**Percentage ending up in residues (average Danish MSWIs)**	%	approx. 36-75 (slag)<br>approx. 3-9 (fly ash)	General values around 99% for copper in 1995 (Lassen *et al.*, 1996) and see line above.
**Release from landfills to municipal waste water treatment**	kg/year	0	Lassen *et al.*, 1996 provides general data for releases of copper from landfills. Specific data on releases from pressure impregnated wood are not available. It is assumed that pressure impregnated wood is disposed of to landfill with discharge of percolate to municipal waste water treatment plants. In our model landfills represent final sinks, a further material fate model for such plants is not considered.
**Direct release from landfills to surface water**	kg/year	0	
**2.6.6 Recycling**			
**Type of recycling activities**	Recycling of pressure impregnated wood is not expected to take place. Small amount of pressure impregnated wood may end up in wood recycled for manufacturing of shipboard.		
**2.6.7 Further parameters for a soil exposure scenario**			
**Model considerations**	The releases of copper carbonate from the treated wood is considered a diffuse source and treated as such in a regional model.<br><br>It should be noted that in the context of the Biocidal Products Regulation (Regulation (EU) 528/2012) preserved wood is considered a point source in contrast to the terminology used in this project where such use is considered a diffuse source. Point sources in the present model are sources which on a regional scale can be attributed to a specific geographic location (e.g. an outlet from a specific sewage treatment plant).		

2.7 Cerium dioxide (CeO_2)

**Cerium dioxide (CeO_2)**			
2.7.1 **General description**			
**General applications**			
CeO2 has several applications and due the catalytic ability of CeO2 to adsorb and release oxygen it is used e.g. to coat the inside of self-cleaning ovens and for hydrogen production in fuel cells. The most widespread use of CeO2 is as an additive to diesel. This use may be particularly important from an environmental point of view, since it may lead to direct emissions during the use phase. For this application CeO2-NP is mixed completely with the diesel (concentration: 5-8 ppm; average particle size: 8-10 nm). The advantage of using CeO2-NP as a fuel catalyst is the improved engine combustion efficiency that results in reduced emissions of soot. CO and NOx. Furthermore, the fuel efficiency has been reported to increase by 8-9 %. The production and use of CeO2 nanoparticles (CeO2-NP) is rapidly growing and CeO2 is used as a fuel additive in countries like the Philippines, New Zealand and the UK. However, the amounts produced and used are at present unknown (Mikkelsen *et al.*, 2o11). Other applications involve its ultraviolet (UV) protective properties and its anti-microbial effects.			
2.7.2 **Manufacturing and import/export of the substance on its own**			
**Manufacturing processes**	Industrial bulk cerium is extracted from mined minerals. primarily monazite and bastnasite and CeO2 is formed by thermal treatment processes		
**Manufacturing in Denmark**			
**Name of parameter**	**Unit**	**Value**	**Remark. data source**
**Import of the substance on its own uses to Denmark**	kg/year		None identified
**Re-export**	% of import		
2.7.3 **Formulation in Denmark**			
**Identified formulation processes in Denmark**	**Application of CeO2 as UV filter in paint and lacquers**		
**Formulation 1: Application of CeO2 as UV filter in paint and lacquers**			
**Number of companies**	companies	<4	
**Quantities used**	kg/year	200	The estimated use of cerium oxide for manufacturing of wood oil in DK is in the range of 100-500 kg/y
**Ending up in final products**	%	97.5%	It is assumed that the dispersion of CeO2 is mixed with no major losses into the final product

**Cerium dioxide (CeO_2)**			
**Release* to municipal waste water system**<br>* Unless otherwise noted the release values were reduced/ enlarged on each side by 50% for the modeling of symmetrical triangular distributions around the specified quantities. The symmetry may possibly be by the absolute border values (highest or lowest possible release value, 1 and 0). In cases were more values are given, the mean is taken as modal value for such distributions.	%	<0,5	The emission scenario document (ESD) for the paint industry from the OECD (2009) assume for manufacture of aqueous dispersion coatings that the total fraction of raw materials lost to waste from the manufactring process is 1.5%. This includes 1% lost due to residues in the mixing vessels and 0.5% due to residues in bags, spills and product returns. It is in the ESD assumed that half of the residue material in the mixing vessels will be re-used in the manufacturing process (recycling. For aqueous dispersion coatings the remaining equipment residue is assumed to be removed in water washings and hence to waste water.<br><br>According to information from Danish manufactures waste water originates from cleaning of tanks and other production equipment. A small part (not quantified) of the total used may be released to the waste water for pre-treatment at the manufacturing sites.<br><br>The first step at all sites is a flocculation where the majority of the CeO2 is precipitated and ends up in a filter cake which is disposed of for external incineration or gasification.<br><br>The pretreated waste water is directed to municipal waste water plants.<br><br>As a worst case estimate, the releases to municipal waste water treatment plants are estimated to be <0,5%. The actual release is probably significantly below this value.
**Direct release to surface water (after internal WW treatment)**	%	0	No direct discharge to surface water
**Direct release to soil**	%	0	No direct releases to soil
**Direct release to air**	%	0	The TiO2 is imported as pastes in which the CeO2 is dispersed in water. The generation of dust by handling of the pastes is considered insignificant.
**Disposed of as solid waste for incineration**	%	2	Filtercake/sludge and CeO2 remaining in packaging are disposed of for incineration
**Transformation during use into other forms**	%	0	Not likely
**Percentage of produced products exported**	% of quantity in final product	No data	It is assumed that all of the product is sold in Denmark

**Cerium dioxide (CeO_2)**			
2.7.4 **Import/export and end-use in articles and mixtures**			
**Identified uses in articles and mixtures**	Global estimations of the content (in percent) of CeO2 in consumer products and articles. As no Danish specific information has been possible to obtain, global information have been used for estimations are included in the table. For a range of the possible uses it has not been possible to estimate the percentage.<br><br>Piccinno *et al.* (2011) estimate the total global production of nano CeOx (all cerium oxides) at 55 t/y (25-75 percentile: 5.5-550 t/y). Hendren *et al.* (2011) indicate as summerized elsewhere (Piccinno *et al.*, 2012) for US values for such a volume a range between 35 and 700 t/y.<br>We computed normally distributed values for the Danish use volume by reffering our computations on the global and US data. A minimal value of 0.03 t/y was fixed based on the ranges indicated above and the global estimation had to be scaled down to European and Swiss conditions based on the proportion of the Gross Domestic Product as suggested by (Sun *et al.*, 2014). The sacling EU-US and Switzerland-Denmark reflects population numbers, due to similar consumption capability of these countries.		

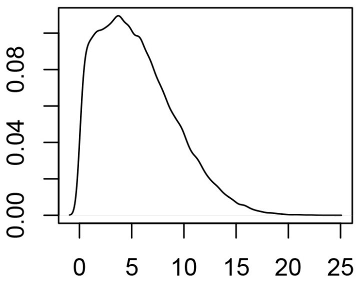

Figure 10. Annual use volumes covering nanomaterial mass of import and own formulation processes Denmark.

	End use	Percentage of total Lower, modal, higher value (l*** ,m, h***)
1	Automotive catalysts converter	16,2**
2	Fuel borne catalyst/fuel additive	1-50*
3	Glass polishing	44,1**
4	Paint and coatings	5-10*
5	NiMH batteries	9,6**
	Medicinal	No data available
	Solar panels and fuel cells (SOFC)	No data available
	Sunscreen and make-up	No data available
	Self-cleaning oven	No data available
	Fluid cracking catalyst	No data available

* Based on a survey covering 239 companies (82% of which were European) (Piccinno, Gottschalk, Seeger, & Nowack, Industrial production quantities and uses of ten engineered nanomaterials in Europe and the world , 2012)
** (Goonan, 2011)
***50% reduction and augmentation

**Name of parameter**	**Unit**	**Value**	**Remark. data source**
**End-use 1: Automotive catalyst converter**			

**Cerium dioxide (CeO_2)**			
**Net-import with articles and mixtures**	kg/year	-	No data available
**Total consumption**	kg/year	780-1,830	The total mass of catalysts in catalyst-containing vehicles registered in Denmark in 2012 is estimated at 342-802 t/y.<br><br>The total consumption of CeO2 is based on the total mass (1st interval) and the percentage of CeO2 mass in the catalysts. (Belcastro, 2012); (Aliexpress, 2013); (Statistics Denmark, 2013)
**Trend in consumption**	Unit-less	Decreasing	Article I. (Wijnhoven, Dekkers, Hagens, & de Jong, 2009)
**Average service life time**	year	12	The average service life of cars in Denmark is 16.3 years (Danmarks Statistik 2010-2011). The service life of car catalysts is expected to be around 75% of the service life of the car. car longevity cannot be used for catalysts longevity
**Release to municipal waste water system**	%	0	The CeO2 is embedded in a solid matrix and a potential release to water can only happen during use (i.e. driving the car or exchange of the catalyst). It is expected to be very unlikely that this will involve a release to the municipal waste water system.
**Direct release to surface water**	%	<1	The CeO2 is embedded in a solid matrix and a potential release to water can only happen during use (i.e. driving the car or exchange of the catalyst). It is expected to be very unlikely that this will involve a direct release to the surface water soil in any significant amounts.
**Direct release to soil**	%	<1	The CeO2 is embedded in a solid matrix and a potential release to water can only happen during use (i.e. driving the car or exchange of the catalyst). It is expected to be very unlikely that this will involve a direct release to soil in any significant amounts.
**Direct release to air**	%	<51 %	(Angelidis & Sklavounos, 1995)
**Disposed of to MSWI**	%	<1	The CeO2 is embedded in a solid matrix and will not be disposed of in the municipal solid waste fraction
**Disposed of to landfill**	%	<1	The CeO2 is embedded in a solid matrix and will not be disposed of in the municipal solid waste fraction
**Disposed of for recycling (excl. energy recovery)**	%	>49%	According to the Danish EPA. all catalyst are recycled; see 60803 Used Catalysts (Miljøstyrelsen, 2009)<br>The reuse process for catalyst materials are not known, but if up to 51% is released to air during use, the remaining 49%will be found in the catalysts for recycling.
**Transformation during use into other forms**	%	-	A transformation of CeAlO3 in the car catalyst has been reported but neither the percentage nor the transformation product were not given (Chen *et al.*, 2011) Ce2(SO4)3 (Zhao *et al.*, 2005)
**End-use 2: Fuel borne catalyst/fuel additive**			
**Total consumption**	kg/year	488	No figures are available to indicate that CeO2 is used as a fuel additive in Denmark. The number listed refers to the calculated amount of CeO2 scaled if it were to be used in Denmark at a level similar to the international use (Mayer, 2008)
**Trend in consumption**	Unit-less	stagnant	
**Average service life time**	year	<0.1	Depending on the refuelling which is expected to be more than once per month.

**Cerium dioxide (CeO_2)**			
**Release to municipal waste water system**	%	0	During use, the loss to the municipal wastewater system is not likely. Spills during refuelling are anticipated to be collected in the collection system at gas stations.
**Direct release to surface water**	%	1	1% release was found by (Johnson & Park, 2012)
**Direct release to soil**	%	<1%	0.06-0.3% release to soil was found by (Johnson & Park, 2012) Futhermore, (Park, et al., 2008) noted that: "No major contamination of the soil would be expected and that soil levels of cerium oxide would be similar of those found naturally."
**Direct release to air**	%	1-5	(Johnson & Park, 2012)
**Disposed of to MSWI**	%	0	Since CeO2 is added to the diesel it is not likely to end up in MSWI
**Disposed of to landfill**	%	0	Since CeO2 is added to the diesel it is not likely to end up in landfills
**Disposed of for recycling (excl. energy recovery)**	%	95-99	In the scrap metal fraction from cars assuming that untransformed CeO2 will be deposited during use in parts of the motor and exhaust system (particle filter as the most likely part).
**Transformation during use into other forms**	%	0	There are no exact figures or estimates for transformation. but some indications are identified in the literature.<br>"Some agglomeration and partial reduction of Ce(IV)."<br>(Jung, Kittelson, & Zachariah, 2005)<br>"n-ceria could impact transformations of other atmospheric species." (Majestic BJ, 2010)<br>"Nanoparticulate cerium dioxide (nano-CeO2). when combusted as an additive to diesel fuel. was transformed from 6 nm to 14 nm sizes into particles near 43 nm. with no obvious change in the unit cell dimensions or crystalline form."<br>(Batley, et al., 2013)
**End-use 3: Glass polishing**			
**Total consumption**	kg/year		It has not been possible to find any specific information in this area about the use in Denmark or how CeO2 is used for this purpose. Therefore, no estimated released has been given below. Release values were taken from glass & ceramics product category of the ZnO case study.
**Release to municipal waste water system**	%	1	
**Direct release to air**	%		
**Disposed of to MSWI**	%	20	
**Disposed of to landfill**	%		
**Disposed of for recycling (excl. energy recovery)**	%	74	
**Transformation during use into other forms**	%	5	
**Export**	%		
**End-use 4: Use of wood oil**			

Cerium dioxide (CeO_2)			
**Total consumption**	kg/year	100-500	Dispersions for preparation of oil containing 0.8 %(w/w) of CeO2 in the formulated product Corresponding to 25,000 liters of formulated product containing 0.8 %(w/w) of CeO2.
**Trend in consumption**	Unit-less	stagnant	
**Average service life time**	year	1	One yearly application can be assumed
**Release to municipal waste water system**	%	<5	The final wood oil is water-based. By application a small part will be discharged to the municipal sewage system by cleaning brushes and other equipment (from less than 1% to a few %).
**Direct release to surface water**	%	<1	Not likely unless a spill occurs.
**Direct release to soil**	%	<5	Related to spill during outdoor application
**Direct release to air**	%	0	After curing of the wood oil the cerium oxide is bound in the polymer matrix. Over time release of larger dust particles and flakes is possible, but the contribution to the overall mass balance is considered to be marginal.
**Disposed of to MSWI**	%	>88	Some 2-10 percent may be disposed of to MSWI with oil left in the container. This kind of wood oil is typically applied by brush and not by air brush.<br>Dust from maintaining the wood is expected to be disposed of the MSWI<br>By the end of its service life the treated wood (not pressure impregnated) is ultimately expected to be disposed of to MSWI
**Disposed of to landfill**	%	0	Not likely for Denmark
**Disposed of for recycling (excl. energy recovery)**	%	0	Not likely for Denmark
**Transformation during use into other forms**	%	0	Not likely under normal use scenarios. Weathering of painted surfaces may cause some release, but transformations are not expected.
**End-use 5: NiMH-batteries**			
**Total consumption**	kg/year	290*	The total mass of NiMH batteries is estimated at 12-98 t/y-<br>The 290 kg/y is an estimate of the amount of CeO 2 in NiMH batteries per / year based on (Miljøstyrelsen, Indsamlingssystemer for batterier, 2003)
**Trend in consumption**	Unit-less	Increasing<br><br>Decreasing	Article II. (Binnemans, et al., 2013)<br>Article III.<br>Article IV. (Miljøstyrelsen, Status for batteriområdet i Danmark, 2005)
**Average service life time**	year	10<br><br>1-1.5	(Binnemans, et al., 2013)<br><br>(forbrug.dk, 2012)
**Release to municipal waste water system**	%	0	CeO2 is incorporated in a solid matrix and release to municipal waste water is not likely during use or disposal.
**Direct release to surface water**	%	0	CeO2 is incorporated in a solid matrix and direct release to surface water is not likely during use or disposal.
**Direct release to soil**	%	5	(Mudgal, et al., 2011)

**Cerium dioxide (CeO_2)**			
**Direct release to air**	%	0	CeO2 is incorporated in a solid matrix and direct release to air is not likely during use or disposal.
**Disposed of to MSWI**	%	22	(Mudgal, et al., 2011)
**Disposed of to landfill**	%	68<br><br>89.3*	Article V. (Mudgal, et al., 2011)<br>Article VI.<br>*Of collected batteries according to (Miljøstyrelsen, Indsamlingssystemer for batterier, 2003)
**Disposed of for recycling (excl. energy recovery)**	%	10<br><br>50<br><br>10.7*	(Mudgal, et al., 2011)<br><br>(Miljøstyrelsen, Status for batteriområdet i Danmark, 2005) In the EPA report it is stated that the current system is the collection rate estimated to be around 50%., which the Environmental Protection Agency deems are not sufficient. From the collected batteries are separated manually, by both private and municipal waste companies; Pb batteries; NiCd batteries; NiMH batteries; and cell batteries for recycling. "<br><br>The 10.7% is an estimate calculated from the 2003 report. i.e. of the collected batteries. (Miljøstyrelsen, Indsamlingssystemer for batterier, 2003)
**Transformation during use into other forms**	%	0	No data available, but transformation during use is not likely
2.7.5 **Waste water treatment**			
**Name of parameter**	**Unit**	**Value**	**Remark. data source**
**Transformation during STP treatment into other forms**	%	-	Unknown percentage, though Westerhof *et al.* (2013) found that interaction with wastewater constituents altered the sorption properties of CeO2. (Westerhoff, Kiser, & Hristovsk, 2013)<br><br>Limbach *et al.* (2008) reported that CeO2 agglomerated strongly in STP
**Percentage ending up in sludge**	% (l. u) uniform distribution	96.6<br><br>94	(Gómez-Rivera, *et al.*, 2012)<br><br>(Limbach, *et al.*, 2008)
**Percentage discharges**	% (l. u) uniform distribution	3.4<br><br>6	(Gómez-Rivera, *et al.*, 2012)<br><br>(Limbach, *et al.*, 2008)
**Processes in surface waters after discharge**	%	98 (out of the emitted)	”CeO2 nanoparticle hetero-aggregate with or deposition onto natural colloids. followed by sedimentation”<br>(Quik. *et al.*. 2012)<br>Modeled based on zwo extreme scenarios on sedimentation 0 and 100%, see comments e.g. for nano-TiO2.
2.7.6 **Solid waste treatment (incineration and landfill)**			
**Name of parameter**	**Unit**	**Value**	**Remark. data source**

**Cerium dioxide (CeO_2)**			
**Transformation or deposition during incineration into other forms (average Danish MSWIs)**	%	approx. 0.1-52 (deposition, transformation)	The CeO2 partition between waste bunker, incinerator, boiler, electrostatic filter, wet scrubber, slag and fly ash was modelled as suggested in (Walser and Gottschalk, 2014).<br>Mass transfer and fate parameters (see please Figure 1) are modelled as shown below in Table 1 that reflects the values derived from computer based simulations combined with real analytic/experimental results. These results show the steady state mass transport/transformation for all relevant WIP paths reached after steady state mode of such plants (infinite time scale). Analytically not detected and not further transported nano-CeO2 has been assigned to the subsequent further transport and/or to the subsequent deposition/transformation by covering at each stage in the WIP process the entire range of transport and fate possibilities. A distinction between material deposition and transformation was not possible due to analytical limitations
**Percentage emitted to the air (average Danish MSWIs)**	%	~0	See line above.
**Percentage ending up in residues (average Danish MSWIs)**	%	approx. 36-75 (slag)<br>approx. 3-9 (fly ash)	See line above.
**Transformation during landfilling into other forms**	%	No data	At this point we stopped our modelling. Nanomaterial fate and behaviour during landfilling was not considered. See also general comments on landfilling.
**Release from landfills to municipal waste water treatment**	kg/year	0	For landfill, no leachate out is assumed, see comments on previous cases.
**Direct release from landfills to surface water**	kg/year	0	See line above.<br><br>Expected to be low due to sorption and straining of CeO2 in the waste matrix. For soils Cornelis *et al.* (2011) found aggregation and sorption to negatively charged soil constituents such as clay (Cornelis, et al., 2011)
2.7.7 **Recycling**			
**Type of recycling activities**	Recycling CeO2 in the batteries		
**Name of parameter**	**Unit**	**Value**	**Remark. data source**
**Transformation during recycling into other forms**	**%**	No data	Not likely during recycling, however recycling of batteries in Denmark (End-use 3) has not shown how the CeO2 in the batteries are recycled.<br><br>At this point we stopped our modelling. Nanomaterial fate and behaviour during recycling was not considered.
**Ending up in recycled products**	**%**	-	No data available
**Release from recycling process**	**% of recycled**	o	See lines above.<br>No data avaiable

2.8 Quantum dots

**Quantum dots**			
2.8.1 **General description**			
**General applications**			
Applications of quantum dots in products and commodities include semiconductors transistors, solar cells, light emitting devices (e.g. LEDs), and diode lasers, medical imaging (and diagnostics/detection) and as possible qubits in quantum computing.			
2.8.2 **Manufacturing and import/export of the substance on its own**			
**Manufacturing processes**	None known, but manufacturing in Denmark cannot be excluded		
**Manufacturing in Denmark**			
**Name of parameter**	**Unit**	**Value**	**Remark. data source**
**Import of the substance on its own uses to Denmark**	kg/year	-	No data available for Denmark.<br>The worldwide production is 600 kg/year<br>(Piccinno, *et al.*, 2012)
**Re-export**	% of import	-	No data available
2.8.3 **Formulation in Denmark**			
**Identified formulation processes in Denmark**	None known. But formulation in Denmark cannot be excluded		
2.8.4 **Import/export and end-use in articles and mixtures**			

Quantum dots

Identified uses in articles and mixtures

The worldwide production is 600 kg/year Piccinno *et al.* (2011) estimates the total global production of quantum dots (QDs) at 0.6 t/y (25-75 percentile: 0.6-5.5 t/y) and the consumption in Europe at the same level. A normal distribution around the European values was modelled with standard deviation 0.1 and by eliminating negative values.

As no Danish specific information has been possible to obtain the possible consumption of quantum dots in final articles is estimated on the basis of European/worldwide consumption figures. When no data is given there is no global estimate available; or the end-use known but not quantified; or in marginal percentages; or placed on a future/merging market where there is not data yet.

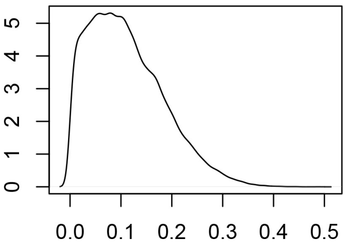

Figure 11. Annual quantum dots use volumes for Denmark.

	End use	Percentage of total<br>Lower. modal. upper value<br>(l**,m,u**)
1	Light conversion for LED/OLED (electronics etc.)	90*
2	Lab use for imaging	10*
	Solar cells	No data
	Biomedical	No data
	Product security and anti-counterfeiting	No data
	Sensors	No data
	Solid-state lighting	No data

* (Piccinno, *et al.*, 2012)

**50% reduction and augmentation up to the absolute limits

**Name of parameter**	**Unit**	**Value**	**Remark. data source**
**End-use 1: Light conversion for LED/OLED**			
**Total consumption**	% of total consump-tion	90	Percentage (mean value) of the total QD use (Piccinno, *et al.*, 2012)
**Trend in consumption**	Unit-less	Increasing	Article VII. (Wijnhoven, Dekkers, Hagens, & de Jong, 2009)
**Average service life time**	year	-	Highly dependent on the type of electronics
**Release* to municipal waste water system**	%	0	QDs are incorporated in a solid matrix in the electronics and are not likely to be released under normal use conditions<br><br>* Unless otherwise noted the release values were reduced/ enlarged on each side by 50% for the modeling of symmetrical triangular distributions around the specified quantities. The symmetry may possibly be by the absolute border values (highest or lowest possible release value, 1 and 0). In cases were more values are given, the mean is taken as modal value for such distributions.

**Quantum dots**			
**Direct release to surface water**	%	0	QDs are incorporated in a solid matrix in the electronics and are not likely to be released under normal use conditions
**Direct release to soil**	%	0	QDs are incorporated in a solid matrix in the electronics and are not likely to be released under normal use conditions
**Direct release to air**	%	0	QDs are incorporated in a solid matrix in the electronics and are not likely to be released under normal use conditions
**Disposed of to MSWI**	%	10	For electronic products nearly 100% will be disposed of for recycling (Miljøstyrelsen, ISAG Udtræksmodul, 2009), however if QDs are used for LED lamps and for these the majority is expected to be disposed of for MSWI (even LED lamps contain electronic compo-nents) will be higher since light sources usually end up in MSWI of which most is incinerated in Denmark.<br>The spilt in QD use between separate light sources (lamps) and LEDs in electronic equipment is not known.
**Disposed of to landfill**	%	0	Electronic will not be landfilled in Denmark
**Disposed of for recycling (excl. energy recovery)**	%	90	The part of the LEDs in electronic equipment is expected nearly 100% to be disposed of for recycling of electronics (Miljøstyrelsen, ISAG Udtræksmodul, 2009)
**Transformation during use into other forms**	%	0	Not likely under normal use scenarios.
**End-use 2: Lab use for imaging**			
**Total consumption**	% of total consump-tion	10	Percentage (mean value) of the total QD use (Piccinno, *et al.*, 2012)
**Trend in consumption**	Unit-less	-	
**Average service life time**	year	-	Highly dependent on the type of electronics
**Release to municipal waste water system**	%	0	QDs are incorporated in a solid matrix in the electronics and are not likely to be released under normal use conditions
**Direct release to surface water**	%	0	QDs are incorporated in a solid matrix in the electronics and are not likely to be released under normal use conditions
**Direct release to soil**	%	0	QDs are incorporated in a solid matrix in the electronics and are not likely to be released under normal use conditions
**Direct release to air**	%	0	QDs are incorporated in a solid matrix in the electronics and are not likely to be released under normal use conditions
**Disposed of to MSWI**	%	0	For electronic products nearly 100% will be disposed of for recycling (Miljøstyrelsen, ISAG Udtræksmodul, 2009)
**Disposed of to landfill**	%	0	Electronic will not be landfilled in Denmark
**Disposed of for recycling (excl. energy recovery)**	%	100	The part of the LEDs in electronic equipment is expected nearly 100% to be disposed of for recycling of electronics (Miljøstyrelsen, ISAG Udtræksmodul, 2009)
**Transformation during use into other forms**	%	0	Not likely under normal use scenarios.
2.8.5 **Waste water treatment**			
**Name of parameter**	**Unit**	**Value**	**Remark. data source**

**Quantum dots**			
**Transformation during STP treatment into other forms**		Possible, but not quantified. We used a zero value for transformation as done also for the nano-TiO2 studies.	Changes to the core/shell structure may occur due to e.g. changes in redox conditions. pH, and light conditions may occur as shown by the following quotes:<br><br>Showed degradation and reduced mobility in soil.<br>(Navarro, Banerjee, Watson, & Aga, 2011)<br><br>"At pH 4.0. the number and the fluorescence of the individual particles decrease significantly. indicating changes in the electronic environment of the ZnS shell and/or dissolution of the QDs."<br>(Slaveykova & Startchev, 2009)<br><br>"quantum dots cause toxicity to bacterial cells by releasing harmful components…"<br>(Jafar & Hamzeh, 2013)<br><br>"slight changes in pH degraded quantum dot coatings. releasing the core metals and killing bacteria."<br>(Mahendra, 2009)<br><br>"most environmental conditions seem to favour QD degradation"<br>(Blickley, 2010)<br><br>"QDs will become hydrophilic when dispersed in bodies of water" The Toxicological Effects of Engineered Nanoparticles. Quantum Dots. in Estuarine Fish.pdf<br>(Blickley, 2010)<br><br>"QDs are also photo - oxidized by visible light."<br>(Blickley, 2010)
**Percentage ending up in sludge**	%	70-80	(Zhang, Chen, Westerhoff, & Crittenden, 2007) reports that the phase distribution of CdTe QD favour the solid phase, hence the majority sediment.
**Percentage discharges**	%	20-30	(Zhang, Chen, Westerhoff, & Crittenden, 2007)
2.8.6 **Solid waste treatment (incineration and landfill)**			
**Name of parameter**	**Unit**	**Value**	**Remark. data source**
**Transformation or deposition during incineration into other forms (average Danish MSWIs)**		approx. 0.1-52 (deposition, transformation)	The incineration model values for metal nanomaterials has been used also for metallic cadmium and zink based quantum dots. See please the details given in the previous CeO_2 case study that is based on Wasler and Gottschalk (2014).
**Percentage emitted to the air (average Danish MSWIs)**		~0	See line above.<br><br>If Cd-QDs in LED lights are incinerated cadmium can be be volatilized, and condense on small particles. (Institute, 1993) The release will depend on the efficiency of the electrostatic filter for cleaning of the flue gas.

**Quantum dots**			
**Percentage ending up in residues (average Danish MSWIs)**	%	approx. 36-75 (slag)<br>approx. 3-9 (fly ash)	See line above
**Release from landfills to municipal waste water treatment**	kg/year	0	Electronics are not expected to be landfilled
**Direct release from landfills to surface water**	kg/year	0	Electronics are not expected to be landfilled
2.8.7 **Recycling**			
**Type of recycling activities**	Not known – but expected to follow the normal procedures for EEE waste.<br>Is it not likely that further environmental release occurs than the QD by the recycling may be melted or dissolved and not further present as a QD as modelled in this study.		
**Name of parameter**	**Unit**	**Value**	**Remark. data source**
**Transformation during recycling into other forms**	**%**	-	No data available
**Ending up in recycled products**	**%**	-	No data available
**Release from recycling process**	**% of recycled**	-	No data available

2.9 Carbon black

Carbon black	
**2.9.1 General description**	
**General applications**	
Worldwide carbon black consumption in 2010 was 9 million t/y and were expected to reach 13 million t/y 2015.<br><br>In the EU the registered production and import of carbon black is in the 1,000,000-10,000,000 t/y tonnage band.<br><br>**Tires and other rubber products -** Globally, approximately 90% of carbon black produced is used in the rubber industry as a reinforcing filler in a variety of products. The 70% is used as a reinforcement in tyres for automobiles and other vehicles, and 20% is used for other rubber products such as hoses, gaskets, mechanical and moulded goods, and footwear (OECD, 2005; Environment Canada, 2013). The production of tires of all sorts consumed over 7.8 million tonnes in 2011 (Ceresana, 2013). In tires and other rubber articles the carbon black is used for reinforcement. Carbon black constitutes approximately 22% of the mass of a tyre (OECD, 2005).<br><br>**Other industrial sectors** - About 9% of the global consumption is used as black pigment in other industrial sectors like plastics, paints, varnishes and printing inks. The remaining 1% is used in hundreds of diverse products, including batteries, high temperature insulating material, and thickeners for certain high temperature petroleum and synthetic greases. In addition, carbon black is used to impart electrical conductivity in rubber and plastics (Environment Canada, 2013)<br><br>According to the U.S. Household Products Database (HPD, 2009 as cited by Environment Canada, 2013), carbon black is used in a variety of household products including paints (liquid and aerosol), primers, stains, paint protectors (i.e., undercoating), rubber gaskets, caulking, concrete repair and sealants, cement colour pigments, fibreglass insulation, pipe seals, shoe polish, laserjet printer toners, inkjet printer cartridges, electronic sealants, and diaper ointment.<br><br>The International Carbon Black Association (ICBA, 2013) indicates the following applications in addition to the applications in rubber mentioned above:<br><br>**Plastics** - Carbon blacks are now widely used for conductive packaging, films, fibres, mouldings, pipes and semi-conductive cable compounds in products such as refuse sacks, industrial bags, photographic containers, agriculture mulch film, stretch wrap, and thermoplastic molding applications for automotive, electrical/electronics, household appliances and blow-moulded containers.<br><br>**Electrostatic Discharge (ESD) Compound**s - Carbon blacks are carefully designed to transform electrical characteristics from insulating to conductive in products such as electronics packaging, safety applications, and automotive parts.<br><br>**High Performance Coatings** - Carbon blacks provide pigmentation, conductivity, and UV protection for a number of coating applications including automotive (primer basecoats and clearcoats), marine, aerospace, decorative, wood, and industrial coatings.<br><br>**Toners and Printing Inks** - Carbon blacks enhance formulations and deliver broad flexibility in meeting specific colour requirements.	
**2.9.2 Manufacturing and import/export of the substance on its own**	
**Manufacturing processes**	The following description is extracted from OECD (2005). The oil furnace black process uses heavy aromatic oils as feedstock. The production furnace is a tightly enclosed reactor used to react the feedstock under carefully controlled conditions and at extremely high temperatures. The feedstock is atomized in a hot gas stream where it vaporizes and then pyrolyzed in the vapour phase to form microscopic carbon particles. In most furnace reactors, the reaction is controlled by steam or water sprays. The carbon black produced is conveyed through the reactor, cooled, and collected in bag filters in a continuous process. Furnace black is available in several grades. They are mainly used in rubber products, inks, paints and plastics.<br>The thermal black process uses natural gas, mainly consisting of methane, as the starting material in a cyclic operation in which the gas is thermally decomposed (cracked). The process uses a pair of furnaces that alternate approximately every five minutes between preheating and carbon production. The methane

**Carbon black**			
	is injected into a hot refractory-lined furnace. In the absence of air, the heat from the refractory material decomposes the methane into carbon black and hydrogen. The aerosol material stream is quenched with water sprays and filtered. The exiting carbon black may be further processed to remove impurities, pelletized, screened, and then packaged for shipment. The process yields relatively coarse particles.<br>Two other processes (the lamp process for production of lampblack and the cracking of acetylene to produce acetylene black) are use for small-volume specialty carbon blacks that constitute less than 1% of the total production. Lampblack is produced by burning liquid hydrocarbons, e.g. kerosene. Lampblack is often oily. It is used for contact brushes in electrical apparatus.		
**Manufacturing in Denmark**	Carbon black is not produced in Denmark		
**Name of parameter**	**Unit**	**Value**	**Remark, data source**
**Import of the substance on its own uses to Denmark**	kg/year	no information	In the trade statistics carbon black is registered together with other forms of carbon not elsewhere specified and specific data on carbon black cannot be extracted
**Re-export**	% of import	no information	
**2.9.3 Formulation in Denmark**			
**Identified formulation processes in Denmark**	According to the SPIN database (SPIN 2013), based on registrations in the Danish Product Registry, the total content of carbon black in mixtures placed on the Danish market for professional purposed in 2010 was as shown in the table below. It is not indicated whether the mixtures placed on the Danish market are produced in Denmark or imported.<br>The Danish Product Register includes substances and mixtures used occupationally and which contain at least one substance classified as dangerous in a concentration of at least 0.1% to 1% (depending on the classification of the substance). Carbon black is not classified and the registration will only occur if the substance is constituent of mixtures which contain other substances classified as dangerous above the indicated limits.<br>The application area "colouring agents" are probably imported agents used in the formulation of lacquers and varnishes, reprographic agents (printing inks), pigments, textiles, cosmetics and adhesives, whereas the other application areas are the final mixtures – either produced in Denmark or imported.<br>Raw materials for manufacture of plastics such as compounds or masterbatches are likely not register in the Product registry.<br>A previous survey on the use of nanomaterials in the Danish industry reports that more than 1 t/y carbon black was used for the manufacture of paints and printing inks, more than 10 t/y was used for textiles. Tønning *et al.* (2014) reports that carbon black is used in the manufacture of cosmetics (mascara, eyelin-er and nail polish).<br>For the current survey a use of more than 1oo t/y for manufactures of paint and varnishes has been confirmed.<br>Based on the available data it is estimated that around (100-400 t/y) carbon black is used for manufacture of various mixtures while 10-50 t/y is used for manufacture of textiles.		

Application area	Content in mixtures placed on the Danish market , tonnes/year *
Colouring agents	201
Reprographic agents (printing inks)	156
Paints, lacquers and varnishes	119
Fillers	30
Construction materials	14
Impregnating materials	6
Adhesives and binding agents	4
Non-agricultural pesticides and preserva-	4

Carbon black

tives**	
Surface treatment	3
Others	5

* Production + import – export
**Assumed to be antifouling paints

For comparison with Danish figures, for the year 2006, the Canadian Chemical Producers' Association (now called Chemistry Industry Association of Canada) emissions inventory reported total carbon black emissions from member companies of 1.1 t/y (CCPA 2006). None of the companies that reported releases were producers of carbon black.

**Name of parameter**	**Unit**	**Value**	**Remark, data source**
**Formulation 1: Production of paint, lacquers, pigments, reprographic agents, adhesives, cosmetics**			
**Number of companies**	companies	10-50	
**Quantities used**	t/year	100-400	Based on the registration of 201 t/y carbon black in colouring agents. The majority is expected to be used for the manufacture of paint and varnishes.
**Ending up in final products**	%	97%	
**Release* to municipal waste water system**<br><br>* Unless otherwise noted the release values were reduced/ enlarged on each side by 50% for the modeling of symmetrical triangular distributions around the specified quantities. The symmetry may possibly be by the absolute border values (highest or lowest possible release value, 1 and 0). In cases were more values are given, the mean is taken as modal value for such distributions.	%	<0,5	The emission scenario document (ESD) for the paint industry from the OECD (2009) assume for manufacture of aqueous dispersion coatings that the total fraction of raw materials lost to waste from the manufacturing process is 1.5%. This includes 1% lost due to residues in the mixing vessels and 0.5% due to residues in bags, spills and product returns. It is in the ESD assumed that half of the residue material in the mixing vessels will be re-used in the manufacturing process (recycling. For aqueous dispersion coatings the remaining equipment residue is assumed to be removed in water washings and hence to waste water.<br><br>According to information from Danish manufactures waste water originates from cleaning of tanks and other production equipment. Approximately 1-2% of the total used may be released to the waste water for pre-treatment/treatment at the manufacturing sites.<br><br>The first step at all sites is a precipitation/flocculation where the majority of the carbon black is precipitated and ends up in a sludge/filter cake which is disposed of for external incineration or gasification.<br><br>The pre-treated waste water is either directed to municipal waste water plants or further treated at the manufacturing sites.<br><br>In the latter case, the waste water is further treated. The waste water is first treated by pre-precipitation tank, then by biological treatment and ultimately by a final polishing.<br><br>As a worst case estimate, the releases to municipal waste water treatment plants or surface water is estimated to be <0,5%. The actual release is probably significantly below this value. The pre-treated waste water is mainly directed to municipal waste water

**Carbon black**			
			treatment plants but a small fraction may be discharged to surface water
**Direct release to surface water (after internal WW treatment)**	%	<0,1	Worst case estimate – the total release is probably significantly below the <0.1%.
**Direct release to soil**	%		The carbon black is imported as pastes in which the TiO2 is dispersed in water. The generation of dust by handling of the pastes is considered insignificant.
**Direct release to air**	%		The carbon black is imported as pastes in which the TiO2 is dispersed in water. The generation of dust by handling of the pastes is considered insignificant
**Disposed of as solid waste for incineration**	%	2	Filtercake/sludge and carbon black remaining in packaging are disposed of for incineration or gasification.
**Disposed of for other waste management**	%		Considered insignificant
**Percentage of produced products exported**	% of quantity in final product	No data	

2.9.4 Import/export and end-use in articles and mixtures

Identified uses in articles and mixtures

The total global carbon black market is approximately 10 million t/y (Ceresana. 2013). No exact data on the consumption in the EU is available (registration toannge band 1-10 million t/y), but most likely it is in the range of 1-4 million t/y.
Assuming the same per capita consumption in Denmark as the EU average, the annual consumption in mixtures and articles would be some 10,000-40,000 t/y (own expert estimate). If 90% is +imported with tyres and other rubber products, the remaining 10% would correspond to approximately 1,000-4,000 t/y. The data from the Product Registry indicates a total consumption of 340 t/y in various mixtures (excluding colouring agents which are expected to be used in production processes). As the carbon black is not assigned a harmonised classification in accordance with the CLP Regulation, the substances would only be registered in the Product Registry if the mixture other classified constituents. For water-based paints, adhesives, XX, etc. the registered tonnage may be significantly underestimated. Furthermore, compounds and masterbatches for plastic manufacture may not be covered by the registration in the Product Registry.

We computed normally distributed values for the Danish use volume by basing our computations on values indiacted above (globally 10 million t/y, 10,000 and 40,000 t/y for Denmark). However, a minimal value of 5’000 t/y was fixed and the global estimation had to be scaled down to European and Swiss conditions based on the proportion of the Gross Domestic Product as suggested elsewhere (Sun *et al.*, 2014). The sacling Denmark Switzerland occurred by using the population volume, since the consumption capability of these two countries is similar.

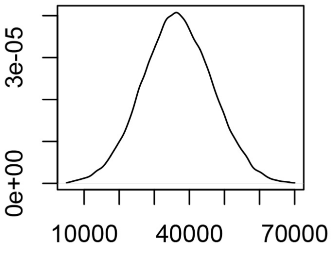

Figure 13. Annual use volumes covering nanomaterial mass of import and own formulation processes Denmark.

Carbon black

	End use	Percentage of total Lower, modal, upper value (l*,m,u*)
1	Tires	35 ,70, 100
2	Other rubber components (various articles)	10, 20, 30
3	Paint and varnishes	1.5, 3, 4.5
4	Antifouling paints	0.05, 0.1, 0.15
5	Inks	1.5, 3, 4.5
6	Plastic components (various articles)	1.5, 3, 4.5
7	Filters	0.1, 0.2, 0.3
8	Other	0.4, 0.7, 1.1
*50% reduction and augmentation		

**Name of parameter**	**Unit**	**Value**	**Remark, data source**
**End-use 1: Tires**			
**Total consumption**	% of total consumption	70	Mean values, based on information on global consumption figures
**Release to municipal waste water system**	%	3	Approximately 22% of a tire is composed of carbon black, and on average a tire loses from 10 to 20% of its weight during use over its service life (OECD, 2006). As it is bound within the elastomer complex, carbon black is unlikely to be released from tires as an unbound particle through wear or abrasion (US EPA 1976; OECD 2006; ChemRisk, Inc. and DIK, Inc. 2008 as cited by Environment Canada, 2013).<br><br>According the Naturstyrelsen (2012) the paved area in areal appox-imately 77.000 hectares, of this 35.500 hectares has common sewerage system and the remaining 41.500 hectares has separate storm water sewerage system.<br>The significant part of dust from the tires will be generated outside areas with sewer systems and here the dust will either be released to soil or to surface water.<br>The 15% lost from the tires are roughly estimated to distributed as follows:<br>4% to soil<br>8% to surface water<br>3% to municipal waste water
**Direct release to surface water**	%	8	Release via separate storm water sewerage system from paved areas and direct loss to surface water from areas without sewer systems
**Direct release to soil**	%	4	Losses to soil around roads in the countryside
**Direct release to air**	%	1	Some of the dust may be considered a release to air but is expected to . Value according to the one for CTNs.
**Disposed of to MSWI**	%	5	Even the majority of tires are disposed of for recycling a small part may end up on MSWI. According to Dækbranchens Miljøfond (2012), 97% of the tires collected in 2011 were recycled. It is not indicated what happened to the remaining 3%. In addition as small

**Carbon black**			
			percentage may be disposed of directly the MSWI without collection
**Disposed of to landfill**	%		Tires are not disposed of to landfills in Denmark
**Disposed of for recycling (excl. energy recovery)**	%	80	[we have not yet identified data showing the collection efficiency of tires in Denmatk]<br>97% of the tires collected in 2011 were recycled (Dækbranchens Miljøfond, 2012)
**Transformation during use into other forms**	%	-	
**End-use 2: Other rubber components (various articles)**			
**Total consumption**	% of total consumption	20	Mean values, based on information on global consumption figures
**Direct release to surface water**	%	<0.1	The releases to the environment and waste water of particles of other rubber products to the environment is considered insignificant but small releases cannot be excluded
**Direct release to soil**	%	<0.1	See above
**Direct release to air**	%	<0.1	See above
**Disposed of to MSWI**	%	95	
**Disposed of to landfill**	%	5	Roughly estimated. Rubber parts of in vehicles (gaskets, hoses, etc.) may be disposed of to landfills in waste from shredder plants
**Disposed of for recycling (excl. energy recovery)**	%	0	The recycling of other rubber products is considered insignificant
**Transformation during use into other forms**	%	-	No data
**End-use 3: Paint and varnishes**			
**Total consumption**	% of total consumption	3	Mean values, based on information on global consumption figures
**Release to municipal waste water system**	%	1	Dust and flakes from maintenance of painted surfaces and from abrasion of painted surfaces
**Direct release to surface water**	%	1	Dust and flakes from maintenance of painted surfaces and from abrasion of painted surfaces
**Direct release to soil**	%	2	Dust and flakes from maintenance of painted surfaces and from abrasion of painted surfaces
**Direct release to air**	%	1	Dust and flakes from maintenance of painted surfaces and from abrasion of painted surfaces
**Disposed of to MSWI**	%	50	Paint remaining in packaging and paint on wood and other combustible materials
**Disposed of to landfill**	%	10	Paint on concrete and other non-combustible building materials
**Disposed of for recycling (excl. energy recovery)**	%	35	Paint on metals
**Transformation during use into other forms**	%		No data

**Carbon black**			
**End-use 4: Antifouling paints**			
**Total consumption**	% of total consumption	0.1	Estimates on the basis of information on carbon black in antifouling paint marketed in Denmark (data from the Product Registry)<br>Antifouling paint is a complicated category as paint applied in Denmark may not necessarily be applied on vessels sailing i Danish waters, and thus not release to Danish waters. At the same time a significant part of the releases to Danish waters is from vessels passing the waters og foreign vessels visiting Danish harbours.<br>A study on the use of organotin compounds for antifouling estimated that the total releases to the Danish waters was less than half of the releases due to organotin applied in Denmark (released in Denmark and elsewhere).<br>In the following for simplicity the releases from antifouling paint used (applied) in Denmark is assumed to be released to Danish waters.
**Release to municipal waste water system**	%		
**Direct release to surface water**	%	67	A study on the use of organotin compounds in antifouling paints in Denmark estimates that 60-70% of the paint is released to the water during use (Lassen *et al.*, 1999). In addition small quantities will be lost by maintenance of boats and vessels.<br>The main part is released to the sea while a very small part is releases to streams and lakes.
**Direct release to soil**	%	0.1	A small percentage may be lost to soil by maintenance of boats
**Disposed of to MSWI**	%	1	Disposed of with dust from maintenance
**Disposed of to landfill**	%		
**Disposed of for recycling (excl. energy recovery)**	%	32	Remaining on the metal ultimately disposed of for recycling
**Transformation during use into other forms**	%		
**End-use 5: Inks**			
**Total consumption**	% of total consumption	3	Mean values, based on information on global consumption figures
**Release to municipal waste water system**	%		
**Direct release to surface water**	%		
**Direct release to soil**	%		
**Direct release to air**	%		
**Disposed of to MSWI**	%	10	In analogy with nanomaterial in paper (nano-TiO2) as estimated elswhere (Sun *et al.*, 2014).
**Disposed of to landfill**	%		
**Disposed of for recycling (excl. energy recovery)**	%	80	See lines above.
**Export**	%	10	See lines above.

**Carbon black**			
**End-use 6: Plastic components (various articles)**			
**Total consumption**	% of total consumption	3	Mean values, based on information on global consumption figures
**Release to municipal waste water system**	%	<0.1	The releases to the environment and waste water of particles of other rubber products to the environment is considered insignificant but small releases cannot be excluded
**Direct release to surface water**	%	<0.1	See above
**Direct release to soil**	%	<0.1	See above
**Disposed of to MSWI**	%	96	The recycling of those plastic parts that contain carbon black (see description in the section on general applications) is considered very small, and the majority is thus disposed of for incineration
**Disposed of to landfill**	%	2	Roughly estimated. Plastic parts in vehicles may be disposed of to landfills in waste from shredder plants
**Disposed of for recycling (excl. energy recovery)**	%	2	The recycling of those plastic parts that contain carbon black (see description in the section on general applications) is considered very small
**Transformation during use into other forms**	%		No data
**End-use 7: Fillers**			
**Total consumption**	% of total consumption	0,2	Mean values, based on information on global consumption figures
**Release to municipal waste water system**	%	25	In analogy to application in filters of nano-TiO2 (Sun *et al.*, 2014)
**Direct release to surface water**	%		
**Direct release to soil**	%		
**Direct release to air**	%	5	See lines above.
**Disposed of to MSWI**	%	70	See lines above.
**Disposed of to landfill**	%		
**Disposed of for recycling (excl. energy recovery)**	%		
**Transformation during use into other forms**	%		
**End-use 8: Other uses – not further considered**			
**2.9.5 Waste water treatment**			
**Name of parameter**	**Unit**	**Value**	**Remark, data source**
**Transformation during STP treatment into other forms**	%		no data – not expected to be significant
**Percentage ending up in sludge**	% (l, m, u)	0, 50, 100	Environment Canada (2013) conservatively estimate the carbon black removal efficiency from influent resulting from the wastewater treatment process at 50% where lagoons or primary treatments exist.
**Percentage discharges**	% (l, m, u)	0, 50, 100	See above.

**Carbon black**			
**2.9.6 Solid waste treatment (incineration and landfill)**			
**Name of parameter**	**Unit**	**Value**	**Remark, data source**
**Transformation during incinera-tion into other forms (average Danish MSWIs)**	%	75, 98, 100	The degradation temperature of carbon black is in principle 3652–3697°C and it is not expected that carbon black will be degraded during incineration under non-oxidative conditions (Environment Canada, 2013).<br>In this work carbon-based materials /CNT and CB) are assumed to almost completely burn under standard oxidative conditions in the furnace (Mueller *et al.*, 2013).<br>Complete combustion is expected for carbon black with low air release and almost no residual ash (ICBA, 2014).
**Percentage emitted to the air (average Danish MSWIs)**	%	<1	Despite the presence of pollution control devices, some dust con-taining carbon black may escape into air. (Environment Canada, 2013). With the filters used in Danish waste incinerators it is expected that less than one percent of the carbon black will be released to the air.
**Percentage ending up in residues (average Danish MSWIs)**	%	~99	It is expected that the majority of the carbon black ends up in the ashes from the flue gas cleaning which as disposed of for land-filling.<br>A minor part is expected to end up in the bottom ashes which to some extent are used for construction works.<br><br>Mass allocation to diferent ashes according to others and as done for CNT: 18 % reaching fly ashes and 81% ending up in bottom ashes (Sun *et al.*, 2014)
**Release from landfills to munici-pal waste water treatment**	kg/year	0	For landfill, no leachate out is assumed.
**Direct release from landfills to surface water**	kg/year	0	See line above.
**Transformation during land-filling into other forms**	%	No data	At this point we stopped our modelling. Nanomaterial fate and behaviour during landfilling was not considered. See also general comments on landfilling.
**2.9.7 Recycling**			
**Type of recycling activities**	Recycling processes include recycling of tires and recycling of printed paper.<br>[to be elaborated]		
**Name of parameter**	**Unit**	**Value**	**Remark, data source**
**Transformation during recycling into other forms**	**%**	No data	No data available. We did not track the material fate and mass flows of the studied nanoparticles during and after the recycling process.
**Ending up in recycled products**	**%**	No data	See lines above.
**Release from recycling process**	**% of recycled**	0	See lines above.

3. References

Al-Salim N, Barraclough E, Burgess E, Clothier B, Deurer M, Green S, Malone L, Weir G. 2011. Quantum dot transport in soil, plants, and insects. Science of the Total Environment; 409: 3237–3248.

Anastasio C, Martin ST. 2001. Atmospheric Nanoparticles, in: Banfield JF, Navrotsky A. (Eds.), Reviews in Mineralogy and Geochemistry, Washington, D.C., pp. 293-349.

ANEC/BEUC, 2010. ANEC/BEUC Inventory of Products Claiming to Contain Nanoparticles Available on the EU Market.

Angelidis T, Sklavounos S. 1995. A SEM-EDS study of new and used automotive catalysts, Thessaloniki, Greece: Aristotle University of Thessaloniki.

AliExpress. [Online] Available at: http://www.aliexpress.com/store/product/Universal-Rounded-shape-TWC-Three-way-catalytic-Converter/119696_835915919.html

Aschberger, K., Micheletti, C., Sokull-Kluettgen, B., Christensen, F.M., 2011. Analysis of currently available data for characterising the risk of engineered nanomaterials to the environment and human health - Lessons learned from four case studies. Environment International 37, 1143-1156.

Azonanano. 2006. Copper Oxide - NanoArc® Copper Oxide Nanomaterials from Nanophase Technologies Corporation. Azonanano.com. Sep 13, 2006.

AZoNano, 2013. http://www.azonano.com/article.aspx?ArticleID=3348.

Batley GE, Halliburton B, Kirby JK, Doolette CL, Navarro D, McLaughlin MJ, Veitch C. 2013. Characterization and ecological risk assessment of nanoparticulate CeO2 as a diesel fuel catalyst. Environ Toxicol Chem;32: 1896-1905.

Belcastro EL. 2012. Life Cycle Analysis of a Ceramic Three-Way Catalytic Converter. Thesis submitted to the faculty of the Virginia Polytechnic Institute and State University

Binnemans K , Jones P T, Blanpain B, Van Gerven T, Yang Y, Walton A, Buchert M. 2013. Recycling of rare earths: a critical review. Journal of Cleaner Production, 51: 1-22.

Blaser SA, Scheringer M, Macleod M, Hungerbühler K. 2008. Estimation of cumulative aquatic exposure and risk due to silver: Contribution of nano-functionalized plastics and textiles. Science of the Total Environment 390, 396-409.

Blickley T M. 2010. The Toxicological Effects of Engineered Nanoparticles, Quantum Dots, in Estuarine Fish, s.l.: Department of Environment Duke University.

Bornhöft NK, Nowack B, Hilty LM. 2013. Material Flow Modelling for Environmental Exposure Assessment – A Critical Review of Four Approaches Using the Comparative Implementation of an Idealized Example. Proceedings of the 27th EnviroInfo 2013 Conference, Hamburg, Germany, September 2–4, 2013.

BUND, 2011. Nanoproduct Database.

Burtscher H, Zürcher M, Kasper A, Brunner M. 2002. Efficiency of flue gas cleaning in waste incineration for submicron particles. In: Mayer A, editor. Proc. Int. ETH Conf. on Nanoparticle Measurement. 52. BUWAL.

Burtscher H, Zürcher M, Kasper A, Brunner M. 2001. Efficiency of flue gas cleaning in waste incineration for submicron particles. Proc. Int. ETH Conf. on Nanoparticle Measurement, Zurich.

Central, D., 2013. Time to Buy a Quantum Dot TV. [Online] Available at: http://www.display-central.com/free-news/display-daily/time-to-buy-a-quantum-dot-tv/

Ceresana. 2013. Market Study: Carbon Black (UC-5605). Ceresana Market Intelligence Consulting. http://www.ceresana.com/en/market-studies/chemicals/carbon-black/

Chen Y-C, Lee KH, Jeng FT. 2011. Aging-induced changes in properties of motorcycle catalytic converterS, Taipei, Taiwan: National Taiwan University.

Civiello M, Wouters P. 2003. Combination of Diesel fuel system architectures and Ceria-based fuel-borne catalysts for improvement and simplification of the Diesel Particulate Filter System in serial applications. Presentation.

Clausen PA, Jacobsen NR, Jensen KA (Eds.) (2012). NANOPLAST Nanoteknologiske materialer og produkter i plastindustrien: Eksponeringsvurdering og toksiske egenskaber. Det Nationale Forskningscenter for Arbejdsmiljø (NFA). (in Danish)

Cornelis G, Ryan B, McLaughlin MJ, Kirby JK, Beak D, Chittleborough D. 2011. Solubility and Batch Retention of CeO2 Nanoparticles in Soils. Environmental Science & Technology 45: 2777-2782.

Dækbranchens Miljøfond. 2012. Nyhedsbrev 12. June 2012.

Danish Nature Agency. 2012. Punktkilder 2011 [Point Sources 2011](in Danish). www.nst.dk.

Danish Nature Agency. 2014. Pers.comm., Anna Gade Holm, Centre for information on point sources, March 2014.

DANVA, 2009b. Vand i tal [Water in figures. Danish Water and Wastewater Association (in Danish).

DANVA. 2009a. Håndtering af spildevandslam. Katalog over metoder til behandling og deponering. [Handling of sewage sludge. Catalogue of methods for treatment and disposal]. Danish Water and Wastewater Association (in Danish)

DEPA. 2009. Affaldsstatistik 2007 and 2008 [Waste Statistics] (in Danish). Danish Environmental Protection Agency.

DEPA. 2010. Deponeringskapacitet I Denmark [Disposal capacity in Denmark] (in Danish). Danish Environmental Protection Agency.

DEPA. 2013. Affaldsstatistik 2011 [Waste Statistics 2011] (in Danish). Danish Environmental Protection Agency.

DEPA. 2012. Bekæmpelsesmiddelstatistik 2011 [Pesticide statistics 2011]. Danish Environmental Protection Agency. http://www2.mst.dk/Udgiv/publikationer/2012/10/978-87-92903-58-7.pdf.

DMU. 2000. Afstrømningsforhold i danske vandløb [catchment and flow conditions of Danish streams] (in Danish). Faglig rapport fra DMU nr 340, 2000.

DuPont. 2010. Titanium dioxide. A brief overview of TiO2 pigments compared with TiO2 nanomaterials, March 2010.

EC21. 2008. Global B2B Marketplace, 2008.

EEA. 2013. Municipal Waste Management in Denmark. European Environment Agency.

Environment Canada. 2013. Screening Assessment for the Challenge. Carbon Black. Chemical Abstracts Service Registry Number 1333-86-4. Environment Canada, Health Canada.

US EPA. 2010. Nanomaterial Case Study: Nanoscale Silver in Disinfectant Spray, External Review Draft. US Environmental Protection Agency.EPA report EPA/600/R-10/081.

EPRUI. 2013. CuO Nanoparticle Features. EPRUI Nanoparticles & Microspheres Co. Ltd. At: http://www.nanoparticles-microspheres.com/

European Commission. 2011. Commission Recommendation of 18 October 2011 on the definition of nanomaterial. http://eur-lex.europa.eu/LexUriServ/LexUriServ.do?uri=CELEX:32011H0696:EN:NOT

Statistics Denmark 2013. Familiernes bilrådighed [the families' access to cars], s.l.: Statistics Denmark.

forbrug.dk. 2012. Typer af batterier[types of batteries][Online] Available at: http://www.forbrug.dk/Artikler/Test-og-raad/Elektronik/Batterier/typerafbatterier?tc=1080018F3D034546B1C4980619759B26 [Senest hentet eller vist den 2013].

Freeman MH, McIntyre CR. 2008. A comprehensive review of copper-based wood preservatives - With a focus on new micronized or dispersed copper systems. Forest Prod Jour, 58 (11): 6-27.

Future Markets. 2011. Future Markets, 2011. The World Market for Carbon Nanotubes, Nanofibers, Fullerenes and POSS. Future Markets, Inc.

Future Markets. 2011a. The world market for nanoparticle titanium dioxide. Future Markets Inc. Abstract available at: http://www.futuremarketsinc.com/index.php?option=com_content&view=article&id=38&Itemid=75

Future Markets. 2011b. The World Market for Carbon Nanotubes, Nanofibers, Fullerenes and POSS. Future Markets, Inc.

Geosyntec Consultants, Inc., and FRx Inc . 2012. Jet injection pilot test summary report.Taastrup, Denmark. Jet Injection Report – 05142012.

Geranio L, Heuberger M, Nowack B. 2009. Behavior of silver nano-textiles during washing. Environ. Sci. Technol. 43: 8113-8118.

GEUS. 2001. Teglværksler i Danmark. GEUS Report no. 2001/92.

Gómez-Rivera F, Field JA, Brown D, Sierra-Alvarez R. 2012. Fate of cerium dioxide (CeO2) nanoparticles in municipal wastewater during activated sludge treatment. Bioresource Technology, 108: 300–304.

Goonan TG. 2011. Rare Earth Elements—End Use and Recyclability, Reston, Virginia: s.n.

Gottschalk F, Scholz RW, Nowack B. 2010a. Probabilistic material flow modeling for assessing the environmental exposure to compounds: Methodology and an application to engineered nano- TiO2 particles. Environmental Modelling & Software; 25: 320-332.

Gottschalk F, Sonderer T, Scholz RW, Nowack B. 2009. Modeled environmental concentrations of engineered nanomaterials (TiO2, ZnO, Ag, CNT, fullerenes) for different regions. Environmental Science and Technololgy; 43: 9216-9222.

Gottschalk F, Sonderer T, Scholz RW, Nowack B. 2010b. Possibilities and Limitations of Modeling Environmental Exposure to Engineered Nanomaterials by Probabilistic Material Flow Analysis. Environ. Toxicol. Chem. 29: 1036-1048.

Gottschalk, F., Ort, C., Scholz, R.W., Nowack, B. 2011. Engineered nanomaterials in rivers – exposure scenarios for Switzerland at high spatial and temporal resolution Environmental Pollution 159: 3439-3445.

Graasbøll S, Toudal JK, Andersen J, Stentsøe S. 2010. Deponeringskapacitet i Danmark – i perioderne 2009 12 og 2013 – 20. Disposal capacity i Denmark - in 2009 -12 and 2013-20. Miljøprojekt 1318. Danish Environment Agency (in Danish).

Guidechem. 2013. http://www.guidechem.com/cas-133/1333-86-4.html.

Gyldendal. 2013. Den Store Danske (Encyklopædi) [The Comprehensive Danish Encyclopedia]. www.denstoredanske.dk.

Hansen OC, Færgemamm H, Møller S, Andersen LK, Poll C. 1997. Træbeskyttelsesmidler og imprægneret træ. Massestrømsanalyse, miljø- og sundhedsvurdering. Working Report from the Danish EPA 57/1997. The Danish Environmental Protection Agency.

Hansen E, Olsen S.2004. Livscyklusvurdering af deponeret affald [Life cycle assessment of landfilled waste]. Environmental Project No. 971. Miljøstyrelsen, 2004. The Danish Environmental Protection Agency.

Healy ML, Dahlben LJ, Isaacs, JA. 2008. Environmental assessment of single-walled carbon nanotube processes. Journal of Industrial Ecology 12: 376-393.

Hefei. 2013. Applications of nano-copper powder. Hefei Quantum Quelle Nano Science & Technology Co.,. At: Ltd. http://www.quantum-nano.com

Hendren CO, Mesnard X, Dröge J, Wiesner MR. 2011. Estimating Production Data for Five Engineered Nanomaterials As a Basis for Exposure Assessment. Environ. Sci. Technol.; 45: 2562-2569.

Hou L, Li K, Ding Y, Li Y, Chen J, Wu X, Li X, 2012. Removal of silver nanoparticles in simulated wastewater treatment processes and its impact on COD and NH4 reduction. Chemosphere 87: 248-252.

Hwang S, Martinez D, Perez P, Rinaldi C. 2011. Effect of surfactant-coated iron oxide nanoparticles on the effluent water quality from a simulated sequencing batch reactor treating domestic wastewater. Environmental Pollution 159: 3411-3415.

ICBA, 2014. Carbon Black User's Guide. Safety, Health, & Environmental Information, International Carbon Black Association. http://www.carbon-black.org/files/carbonblackuserguide.pdf.

ICBA. 2013. Overview of Uses. International Carbon Black Association. http://www.carbon-black.org/index.php/carbon-black-uses

Information, O. N. a., 2013. Sony's new A58 DSLR uses an SVGA OLED viewfinder, maker unknown. [Online] . Available at: http://www.oled-info.com/sonys-new-a58-dslr-uses-svga-oled-viewfinder-maker-unknown. [Senest hentet eller vist den 2013].

Institute MR. 1993. Locating and Estimating Air Emissions From Sources of Cadmium and Cadmium Compounds, North Carolina, USA.

Jafar G, Hamzeh G. 2013. Ecotoxicity of Nanomaterials in Soil. Annals of Biological Research; 4: 86-92.

Johnson AC, Bowes MJ, Crossley A, Jarvie HP, Jurkschat K, Jürgens MD, Lawlor AJ, Park B, Rowland P, Spurgeon D, Svendsen C, Thompson IP, Barnes RJ, Williams RJ, Xu N.., 2011. An assessment of the fate, behaviour and environmental risk associated with sunscreen TiO(2) nanoparticles in UK field scenarios. Science of the Total Environment 409: 2503-2510.

Johnson AC, Park B. 2012. Predicting contamination by the fuel additive cerium oxide engineered nanoparticles within the United Kingdom and the associated risks, Wallingford, Oxfordshire, United Kingdom: Centre for Ecology and Hydrology.

Jung H, Kittelson DB, Zachariah MR. 2005. The influence of a cerium additive on ultrafine diesel particle emissions and kinetics of oxidation, Minneapolis, USA: Departments of Mechanical Engineering and Chemistry, University of Minnesota.

Kaegi R, Sinnet B, Zuleeg S, Hagendorfer H, Mueller E, Vonbank R, Boller M, Burkhardt M. 2010. Release of silver nanoparticles from outdoor facades. Environmental Pollution; 158: 2900-2905.

Kaegi R, Ulrich A, Sinnet B, Vonbank R, Wichser A, Zuleeg S, Simmler H, Brunner S, Vonmont H, Burkhardt M, Boller M. 2008. Synthetic TiO2 nanoparticle emission from exterior facades into the aquatic environment. Environ. Pollut.; 156: 233-239.

Kaegi R, Voegelin A, Sinnet B, Zuleeg S, Hagendorfer H, Burkhardt M, Siegrist H. 2011. Behavior of Metallic Silver Nanoparticles in a Pilot Wastewater Treatment Plant. Environmental Science & Technology; 45: 3902-3908.

Kannan N, White SM, Whelan MJ, 2007. Predicting diffuse-source transfers of surfactants to surface waters using SWAT. *Chemosphere*; 66: 1336-1345.

Kiaune L, Singhasemanon N. 2011. Pesticidal Copper (I) Oxide: Environmental fate and aquatic toxicity. Reviews of Environmental Contamination and Toxicology 213: 1-26.

Kirkeby J, Gabriel S, Christensen TH. 2005. Miljøvurdering af genanvendelse og slutdisponering af spildevandsslam - en livscyklus screening af fire scenarier [Environmental assessment of recycling and disposal of sewage sludge - a lifecycle screening of four scenarios] (in Danish); Institute of Environment & Resources, DTU.

Kiser MA, Ladner DA, Hristovski KD, Westerhoff PK. 2012. Nanomaterial Transformation and Association with Fresh and Freeze-Dried Wastewater Activated Sludge: Implications for Testing Protocol and Environmental Fate. Environmental Science & Technology 46: 7046-7053.

Kiser MA, Ryu H, Jang H, Hristovski K, Westerhoff P. 2010. Biosorption of nanoparticles to heterotrophic wastewater biomass. Water Research 44: 4105-4114.

Kiser MA, Westerhoff P, Benn T, Wang Y, Perez-Rivera J, Hristovski K. 2009. Titanium Nanomaterial Removal and Release from Wastewater Treatment Plants. Environmental Science & Technology; 43: 6757–6763.

KL & Ministry of the Environment (2010). Rapportering fra arbejdsgruppe om spildevandsindsats i forhøring af vandplan-er/indsatsprogrammer [Report from a working group regarding the performance related to sewage treatment as part of the consultations in connection with the planning in the water sector]. Organisation of the Municipalities (KL) & Ministry of the Environment.(in Danish)

Lassen C, Drivsholm T, Hansen E, Rasmussen B, Christensen K. 1996. Miljøprojekt 323. Danish Environmental Protection Agency.

Lassen C, Vaaben S and Hansen E. 1999. Massestrømsanalyse for tin med særligt fokus på organotinforbindelser. Arbejdsrapport fra Miljøstyrelsen nr. 7/1997.

Lem KW, Choudhury A, Lakhani, AA, Kuyate, P, Haw, JR, Lee, DS, Iqbal Z, Brumlik CJ. 2012. Use of Nanosilver in Consumer Products. Recent Patents on Nanotechnology 6: 60-72.

Limbach LK, Bereiter R, Müller E, Krebs R, Gälli R, Stark WJ. 2008. Removal of oxide nanoparticles in a model wastewater treatment plant: Influence of agglomeration and surfactants on clearing efficiency. Environmental Science & Technology 42: 5828-5833.

LLC, B. R., 2011. Quantum Dots: Global Market Growth and Future Commercial Prospects. [Online] Available at: http://www.bccresearch.com/market-research/nanotechnology/quantum-dots-market-prospects-nan027c.html .

Lombi E, Donner E, Tavakkoli E, Turney TW, Naidu R, Miller BW, Scheckel KG. (2012). Fate of Zinc Oxide Nanoparticles during Anaerobic Digestion of Wastewater and Post-Treatment Processing of Sewage Sludge. Environmental Science & Technology; 46: 9089-9096.

Lorenz C, Windler L, von Goetz N, Lehmann RP, Schuppler M, Hungerbühler K, Heuberger M, Nowack B. 2012. Characterization of silver release from commercially available functional (nano)textiles. Chemosphere; 89: 817-824.

Lowry MS, Hubble DR, Wressell AL, Vratsanos MS, Pepe FR, Hegedus CR. 2008. Assessment of UV-permeability in nano-ZnO filled coatings via high throughput experimentation. Journal of Coatings Technology and Research 5, 233-239.

Mahendra DS. 2009. Finding a home in Los Angeles and at UCLA. Civil & Environmental Engineering.

Majestic BJ, Erdakos GB, Lewandowski M, Oliver KD, Willis RD, Kleindienst TE, Bhave PV. 2010. A review of selected engineered nanoparticles in the atmosphere: sources, transformations, and techniques for sampling and analysis. Int J Occup Environ Health; 16:488-507.

Mayer U B E A (ed.). 2008. Particle Filter Retrofit for all Diesel Engines With 319 Illustrations and 40 tables. Expert Verlag.

Mikkelsen S, Hansen E, Baun A, Hansen SF, Binderup M-L. 2011. Survey on basic knowledge about exposure and potential environ-mental and health risks for selected nanomaterials. Environmental Project No. 1370. Danish EPA, Copenhagen.

Miljøstyrelsen, 2003. Indsamlingssystemer for batterier. [Online] Available at: http://www2.mst.dk/common/Udgivramme/Frame.asp?http://www2.mst.dk/udgiv/Publikationer/2003/87-7972-510-4/html/helepubl.htm.

Miljøstyrelsen, 2005. Status for batteriområdet i Danmark. [Online] Available at: http://www2.mst.dk/common/Udgivramme/Frame.asp?http://www2.mst.dk/udgiv/publikationer/2005/87-7614-630-8/html/kap03.htm.

Miljøstyrelsen, 2009. ISAG Udtræksmodul. [Online] . Available at: http://www2.mst.dk/databaser/isag/Default.asp.

Mudgal S, Le Guern Y, Tinetti B, Chanoine A, Pahal S, Witte F. 2011. Comparative Life-Cycle Assessment of nickel-cadmium (NiCd) batteries used in Cordless Power Tools (CPTs) vs. their alternatives nickel-metal hydride (NiMH) and lithium-ion (Li-ion) batteries, Paris, France: BIO Intelligence Service.

Mueller NC, Buha J, Wang J, Ulrich A, Nowack, B. 2013. Modeling the flows of engineered nanomaterials during waste handling. Environmental Science-Processes & Impacts 15: 251-259.

Müller NC, Nowack B. 2010. Nano zero valent iron – THE solution for water and soil remediation? ObservatoryNANO focus report 2010.

NanoIron. (2013). http://www.nanoiron.cz/. Accessed 17 June 2013.

Naturstyrelsen. 2012. Punktkilder 2011.

Navarro DA, Banerjee S, Watson DF, Aga DS, 2011. Differences in Soil Mobility and Degradability between Water-Dispersible CdSe and CdSe/ZnS Quantum Dots. Environmental Science & Technology, 45: 6343-6349.

Nightingale P, Morgan M, Rafols I, van Zwanenberg P, 2008. Nanomaterials Innovation Systems: Their Structure, Dynamics and Regulation. Report for the Royal Commission on Environmental Pollution (RCEP). SPRU, Science and Technology Policy Research Freeman Centre, University of Sussex, UK, Sussex.

O'Brien NJ, Cummins EJ. 2010. A Risk Assessment Framework for Assessing Metallic Nanomaterials of Environmental Concern: Aquatic Exposure and Behavior. *Risk Analysis* 31: 706-726.

OECD. 2006. Carbon black. SIDS initial assessment report for carbon black; CAS No. 1333-86-4. Organisation for Economic Co-operation and Development, Paris.

OECD. 2009. Emission scenario documents on coating industry (Paints, Laquers and Varnishes). OECD series on emission scenario documents. Number 22. Organisation for Economic Co-operation and Development, Paris.

Osmose. 2013. Personal communication with Lars Nyborg, Osmose Danmark A/S, March 2008.

Park B, Donaldson K, Duffin R, Tran L, Kelly F, Mudway I, Morin JP, Guest R, Jenkinson P, Samaras Z, Giannouli M, Kouridis H, Martin P. 2008. Hazard and Risk Assessment of a Nanoparticulate Cerium Oxide-Based Diesel Fuel Additive—A Case Study. Inhal Toxicol;20(6):547-566.

Piccinno F, Gottschalk F, Seeger S, Nowack B. 2012. Industrial Production Quantities and Uses of Ten Engineered Nanomaterials in Europe and the World. Journal of Nanoparticle Research; 14: 1109.

Pira (2010). The future of carbon black to 2015 – global market forecasts. Pira International Ltd, Leatherhead, Surrey,

Praetorius A, Scheringer M, Hungerbühler K. 2012. Development of Environmental Fate Models for Engineered Nanoparticles—A Case Study of TiO2 Nanoparticles in the Rhine River. Environmental Science & Technology; 46: 6705-6713.

Praetorius A, Gottschalk F, Scheringer M, Sani-Kast N, Nowack B, Hungerbuehler K., *in rev.* Modelling the fate of titanium dioxide, silver and zinc oxide nanoparticles in Swiss rivers at high spatial resolution†. *Environmental Science: Processes & Impacts*.

Preston A, Jin L, Nicholas D, Zahora A, Walcheski P, Archer K, Schultz T. 2008. Field Stake Tests with Copper-based Preservatives. Paper prepared for the IRG 39th Annual Meeting, Istanbul, Turkey, 25-29 May 2008.

QDVision, u.d. Product Safety. [Online]. Available at: http://www.qdvision.com/content1583- [Senest hentet eller vist den 2013].

Ray PC, Yu HT, Fu PP. 2009. Toxicity and Environmental Risks of Nanomaterials: Challenges and Future Needs. *Journal of Environmental Science and Health Part C-Environmental Carcinogenesis & Ecotoxicology Reviews* 27: 1-35.

READE, 2013. http://www.reade.com.

Reijnders L. 2005. Disposal, uses and treatments of combustion ashes: a review. Resources Conservation and Recycling 43: 313-336.

Ren G, Hu D, Cheng EW, Vargas-Reus MA, Reip P, Allaker RP. 2009. Characterisation of copper oxide nanoparticles for antimicrobial applications. Int J Antimicrob Agents, 33:587-590.

RenoDjurs I/S. 2013. Redegørelse om miljø, arbejdsmiljø og kvalitet for 2012 [Report on environment, occupational health and quality 2012] (in Danish). RenoDjurs I/S.

Robichaud CO, Uyar AE, Darby MR, Zucker LG, Wiesner MR. 2009. Estimates of Upper Bounds and Trends in Nano-TiO2 Production As a Basis for Exposure Assessment. Environmental Science & Technology; 43: 4227-4233.

Sahasrabudhe N. 2010. Chemical Information Call-in Candidate: Nano Silver Department of Toxic Substances control; San Francisco.

Scheringer M, MacLeod M, Behra R, Sigg L, Hungerbuehler K. 2010. Environmental risks associated with nanoparticulate silver used as biocide. Household & Personal Care Today 1: 34-37.

Schmid K, Riediker M. 2008. Use of nanoparticles in Swiss industry: A targeted survey. Environ. Sci. Technol.; 42: 2253-2260.

Sengül H, Theis TL. 2009. Life Cycle Inventory of Semiconductor Cadmium Selenide Quantum Dots for Environmental Applications. Nanotechnology Applications for Clean Water, p. 561–582.

Siddique SN. 2013. Simulation of mobility and retention of selected engineered nanoparticles beneath landfills, London, Ontario, Canada: The University of Western Ontario.

SIGMA, 2013. http://www.sigmaaldrich.com/catalog/product/aldrich/704121?lang=de&region=CH.

Slaveykova V I, Startchev K. 2009. Effect of natural organic matter and green microalga on carboxyl-polyethylene glycol coated CdSe/ZnS quantum dots stability and transformations under freshwater conditions. Environmental Pollution; 157: 3445–3450.

Sørensen G *et al. In press* 2014. Prevalence and effects of nano-sized anatase titanium dioxide. Environmental Projects , Danish Environmental Protection Agency.

Statestik D, 2012. Danmark i tal 2012, København: Rosendahls-Schultz.

Statistics Denmark (2013). [Danmarks Statistik]. www.statistikbanken.dk

Steinfeldt M, Gottschalk F, Wigger H, von Gleicha A. 2013. Environmental exposure to engineered nanomaterial from four applications: nanoTiO2 in paint, nanoZnO in glass coatings, MWCNT in epoxy plates, and nanocellulose as a paper additive, Project report of the work package 4 of the NanoSustain project. Bremen.

Stirling R, Drummond J, Zhang Z, Ziobro RJ. 2008. Micro-Distribution of Micronized Copper in Southern Pine. Paper prepared for the IRG 39th Annual Meeting, Istanbul, Turkey, 25-29 May 2008.

Sun TY, Gottschalk F, Hungerbühler K, Nowack B. 2014. Comprehensive probabilistic modelling of environmental emissions of engineered nanomaterials. Environmental Pollution 185: 69-76.

Tardif F, Gaultier V, Shuster F. 2012. International conference on safe production and use of nanomaterials, Minatec, Grenoble, France: www.nanosafe.org.

Tiede K, Boxall ABA, Wang X, Gore D, Tiede D, Baxter M, David H, Tear SP, Lewis J. 2010. Application of hydrodynamic chromatography-ICP-MS to investigate the fate of silver nanoparticles in activated sludge. Journal of Analytical Atomic Spectrometry 25: 1149-1154.

Tønning K, Poulsen M. 2007. Nanotechnology in the Danish industry – Survey on production and application. Environmental Project No. 1206 2007. Danish Environmental Protection Agency.

Tønning K, Sørensen G, Fischer CH, Kristensen HV, Sørensen G. 2014. Supplementary survey of products on the Danish market containing nanomaterials. Environmental Projects 1581, Danish Environmental Protection Agency.

U.S. Research Nanomaterials, 2013. Copper (Cu) Nanopowder / Nanoparticles (Cu, 99.9%, 70nm, metal basis. U.S. Research Nanomaterials, Inc., 2013. Web site: http://www.us-nano.com/inc/sdetail/160

US EPA 2009. Nano-Enabled Environmental Applications for Radionuclides. Office of Radiation and Indoor Air Radiation Protection Division (6608J). EPA 402-R-09-002.

US EPA. 2010a. Nanomaterial Case Studies: Nanoscale Titanium Dioxide in Water Treatment and in Topical Sunscreen.Federal Register.US Environmental Protection Agency.Report EPA/600/R-09/057F., pp. 38188-38190.

US EPA. 2010b. Nanomaterial Case Study: Nanoscale Silver in Disinfectant Spray, External Review Draft. US Environmental Protection Agency. US EPA report EPA/600/R-10/081.

US EPA. 2012. Nanomaterial Case Studies: Nanoscale Titanium Dioxide in Water Treatment and in Topical Sunscreen. US Environmental Protection Agency.

Virksomhedernes Miljøguide (Environmental guide for entreprises) (2013): Slagger fra affaldsforbrændingsanlæg (Bottom ash from waste incinerators).

Walser T, Limbach LK, Brogioli R, Erismann E, Flamigni L, Hattendorf B, Juchli M, Krumeich F, Ludwig C, Prikopsky K, Rossier M, Saner D, Sigg A, Hellweg S, Günther D, Stark WJ. 2012a. Persistence of engineered nanoparticles in a municipal solid-waste incineration plant. Nat Nano; 7: 520-524.

Walser T, Limbach LK, Brogioli R, Erismann E, Flamigni L, Hattendorf B, Juchli M, Krumeich F, Ludwig C, Prikopsky K, Rossier M, Saner D, Sigg A, Hellweg S, Guenther D, Stark WJ. 2012. Persistence of engineered nanoparticles in a municipal solid-waste incineration plant. Nature Nanotechnology 7: 520-524.

Walser, T., Gottschalk, F., 2014. Stochastic fate analyis of engineered nanoparticles in incineration plants. Journal of Cleaner Production. 80, 241-251.

Wang Y, Westerhoff P, Hristovski KD. 2012. Fate and biological effects of silver, titanium dioxide, and C-60 (fullerene) nanomaterials during simulated wastewater treatment processes. Journal of Hazardous Materials 201: 16-22.

Wang ZL. 2004. Zinc oxide nanostructures: growth, properties and applications. Journal of Physics-Condensed Matter 16, R829-R858.

Weir A, Westerhoff P, Fabricius L, Hristovski K, von Goetz N. 2012. Titanium dioxide nanoparticles in food and personal care products. Environ Sci Technol; 46(4):2242-2250.

Westerhoff P, Song GX, Hristovski K, Kiser MA. 2011. Occurrence and removal of titanium at full scale wastewater treatment plants: implications for TiO(2) nanomaterials. Journal of Environmental Monitoring; 13: 1195-1203.

Westerhoff PK, Kiser MA, Hristovsk K. 2013. Nanomaterial Removal and Transformation During Biological Wastewater Treatment. Environmental Engineering Science; 30(3): 109-117.

Wijnhoven S, Dekkers S, Hagens WI, de Jong WH. 2009. Exposure to nanomaterials in consumer products, Bilthoven: National Institute for Public Health and the Environment.

Wikimedia, 2013. http://upload.wikimedia.org/wikipedia/commons/1/13/Zinc_oxide.jpg.

Wikipedia, 2013. http://en.wikipedia.org/wiki/File:Mutr-nanotubes1.jpg.

Windler L, Height M, Nowack, B. 2013. Comparative evaluation of antimicrobials for textile applications. Environment International 53: 62-73.

Woodrow Wilson Institute. 2008. An inventory of nanotechnology-based consumer products currently on the market.

Woodrow Wilson Institute. 2012. The Project on Emerging Nanotechnology Inventory.

WWI, 2012. The Project on Emerging Nanotechnology Inventory.

Zhang Y, Chen YS, Westerhoff P, Hristovski K, Crittenden JC. 2008. Stability of commercial metal oxide nanoparticles in water. Water Research 42: 2204-2212.

Zhang, S., Saebfar, H., 2010. Chemical Information Call-in Candidate: Nano Zinc Oxide, Department of Toxic Substances Control, California, San Francisco.

Zhao D, Chan A, Ljungström E. 2005. Performance study of 48 road-aged commercial threeway catalytic converters, Göteborg, Sweden: Department of Chemistry, Göteborg University.
